# Multi-modality data-driven analysis of diagnosis and treatment of psoriatic arthritis

**DOI:** 10.1038/s41746-023-00757-3

**Published:** 2023-02-02

**Authors:** Jing Xu, Jiarui Ou, Chen Li, Zheng Zhu, Jian Li, Hailun Zhang, Junchen Chen, Bin Yi, Wu Zhu, Weiru Zhang, Guanxiong Zhang, Qian Gao, Yehong Kuang, Jiangning Song, Xiang Chen, Hong Liu

**Affiliations:** 1 Department of Dermatology, Xiangya Hospital, Central South University, Changsha, Hunan China; 2 National Engineering Research Center of Personalized Diagnostic and Therapeutic Technology, Changsha, Hunan China; 3 Monash Biomedicine Discovery Institute and Department of Biochemistry and Molecular Biology, Monash University, Melbourne, VIC Australia; 4 Monash Data Futures Institute and Department of Biochemistry and Molecular Biology, Monash University, Melbourne, VIC Australia; 5 Hunan Key Laboratory of Skin Cancer and Psoriasis, Changsha, Hunan China; 6 Hunan Engineering Research Center of Skin Health and Disease, Changsha, Hunan China; 7 Department of Medicine, Brigham and Women’s Hospital, Harvard Medical School, Boston, MA USA; 8 Monash Biomedicine Discovery Institute and Department of Microbiology, Monash University, Melbourne, VIC Australia; 9 Department of Research and Development, Beijing GAP Biotechnology Co., Ltd, Beijing, China; 10 Department of Clinical Laboratory, Xiangya Hospital, Central South University, Changsha, Hunan China; 11 Department of Rheumatology and Immunology, Xiangya Hospital, Central South University, Changsha, Hunan China; 12 Department of General Medicine, Xiangya Hospital, Central South University, Changsha, Hunan China

**Keywords:** Risk factors, Diagnosis

## Abstract

Psoriatic arthritis (PsA) is associated with psoriasis, featured by its irreversible joint symptoms. Despite the significant impact on the healthcare system, it is still challenging to leverage machine learning or statistical models to predict PsA and its progression, or analyze drug efficacy. With 3961 patients’ clinical records, we developed a machine learning model for PsA diagnosis and analysis of PsA progression risk, respectively. Furthermore, general additive models (GAMs) and the Kaplan–Meier (KM) method were applied to analyze the efficacy of various drugs on psoriasis treatment and inhibiting PsA progression. The independent experiment on the PsA prediction model demonstrates outstanding prediction performance with an AUC score of 0.87 and an AUPR score of 0.89, and the Jackknife validation test on the PsA progression prediction model also suggests the superior performance with an AUC score of 0.80 and an AUPR score of 0.83, respectively. We also identified that interleukin-17 inhibitors were the more effective drug for severe psoriasis compared to other drugs, and methotrexate had a lower effect in inhibiting PsA progression. The results demonstrate that machine learning and statistical approaches enable accurate early prediction of PsA and its progression, and analysis of drug efficacy.

## Introduction

Psoriasis is a chronic and incurable inflammatory disease^1^ with different clinical subtypes, such as psoriasis vulgaris, erythrodermic psoriasis, and pustular psoriasis^2^. Around 25.0% of psoriasis patients will develop psoriatic arthritis (PsA), which is a chronic inflammatory joint disease associated with psoriasis and is featured by its irreversible joint symptoms in addition to skin lesions^1,3–5^. However, psoriasis can develop many other musculoskeletal symptoms that are unrelated to PsA, including osteoporosis, gout, fibromyalgia, soft tissue rheumatism, osteoarthritis, *etc*., leading to inevitable misdiagnosis or missed diagnosis^6^. It is estimated that up to 24.6% of PsA patients suffer from moderate to severe symptoms^7^. The clinical manifestations of PsA are diverse and can invade peripheral joints of the limbs, the trunk joints, and the attachment points of bones, tendons, and even muscles. Such invasions often lead to different degrees of swelling, pain, and discomfort in the axial joint or limb joint^6^, bringing great pain and posing a heavy disease burden to the patient’s life. While the early damage to the joints often appears as joint synovitis and progressive cartilage^8^, the damage may progress to irreversible bone destruction in the middle and late stages, eventually leading to disability^4^. In addition to the similar skin lesions as psoriasis causes, PsA patients are also at greater risk of cardiovascular disease^9^, cardiometabolic syndrome^10^, type 2 diabetes^11^, and liver disease^12^ than healthy individuals. The chronic painful muscle and joint stimulation can also lead to increased anxiety and even depression in PsA patients^13^.

Early diagnosis and control of PsA are therefore critically important^2^. At Xiangya Hospital in China, categorical diagnosis of PsA is usually completed by a multi-disciplinary treatment clinician team, assisted by the ClASsification criteria for Psoriatic ARthritis (CASPAR) standard released in 2006. Patients who meet three or more criteria in the standard can be categorized as PsA patients, with a specificity of 98.7% and a sensitivity of 91.4%, respectively^14^. Predicting the risk of PsA and diagnosing PsA prior to joint symptoms will significantly benefit PsA control. A current challenge in diagnosing PsA lies in the lack of simple, rapid, and specific indicators, for example, (i) the changes in finger (toe) nails are often misdiagnosed as onychomycosis, while early symptoms of joint swelling and pain are often misdiagnosed as rheumatoid arthritis (RA) or osteoarthritis^15^; (ii) irreversible bone destruction may have already been caused in PsA patients when the joint symptoms appear, causing a delay in treatment^5^; and (iii) most psoriatic skin lesions of PsA appear earlier than joint lesions with various manifestations, which can be any type, such as vulgar, pustular, or erythematous^6^. To date, no study has been conducted to clearly show the exact mechanism and timing of the occurrence of PsA, which is one of the bottlenecks in the early diagnosis and treatment of PsA^15^.

It is evident that the incidence of PsA is associated with certain clinical features, such as age and body mass index (BMI)^7,16,17^. It is therefore of great importance to systematically predict and analyze PsA using clinical features based on machine learning techniques. To this end, here we collected the full clinical records of 3961 patients who were diagnosed with psoriasis and treated at Xiangya Hospital of Central South University, China, from January 2017 to August 2021. Based on the clinical records of these patients, we constructed a set of machine learning models and statistical analyses for (i) distinguishing PsA patients from other types of psoriasis (i.e., non-PsA) patients; (ii) predicting the risk of progression to PsA in three years based on patients’ follow-up data; and (iii) statistically analyzing the drug efficacy in the treatment of psoriasis. More importantly, we performed feature analysis to identify the characteristic signatures that discriminate between PsA and non-PsA psoriasis patients and that affect the pathogenesis and progression of PsA. We anticipate that the proposed machine learning models and statistical analysis can serve as a steppingstone for the early diagnosis and treatment of PsA and other types of psoriasis.

## Results

### Overview of the patient cohort and study design

**Fig. 1:**
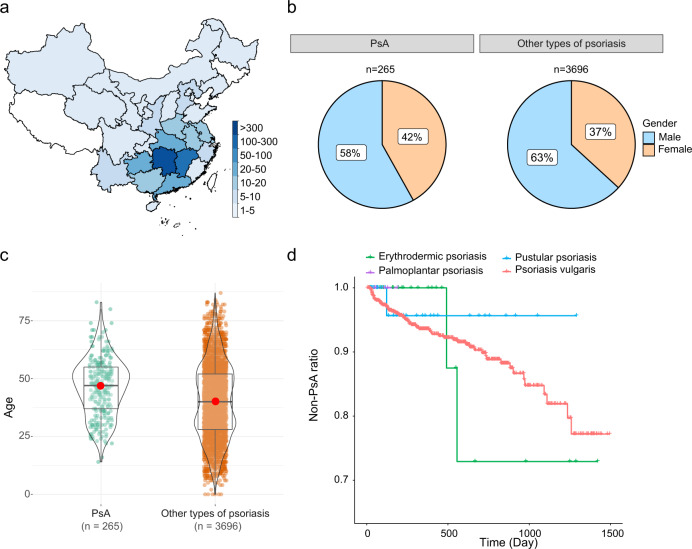
An overview of the psoriasis dataset collected from Xiangya Hospital for this study. **a** The geographical distribution of patients with psoriasis. **b** A comparison of gender distributions between patients with PsA and non-PsA. **c** A comparison of age distributions between patients with PsA and non-PsA. The central line marks the median age value, and the bounds of the box mark the first and third quartiles. **d** Development from other types of psoriasis to PsA within the timeframe.

**Fig. 2:**
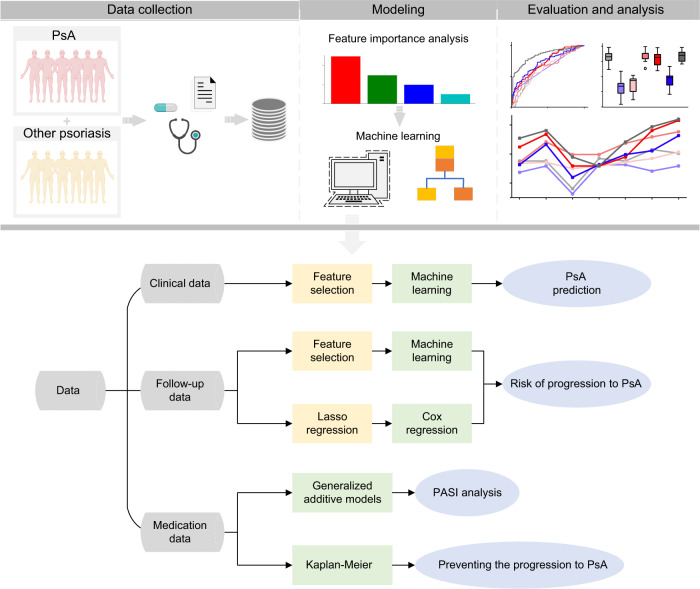
A graphical illustration of this study. The patients’ clinical data collected from Xiangya Hospital were utilized for three different tasks: PsA diagnosis, PsA progression prediction, and drug efficacy analysis of psoriasis treatment.

We constructed and assembled a large-scale cohort dataset with 104 clinical features of 3961 patients admitted to Xiangya Hospital, Changsha, China. These clinical features were clustered into four categories, including physical examination (PE) results, indices for severity and extent of psoriasis (ISEP), laboratory test (LT), and patients’ consultation time and corresponding drug (Supplementary Table 1). The corresponding full terms and descriptions are provided in Supplementary Table 2. The distributions of the features among patients are shown in Supplementary Tables 3–4. Of these 3961 patients, 3241 (81.8%) are from Hunan Province, China, and 267 (6.7%) are from Jiangxi Province. The numbers of patients from other provinces are all less than 50. A detailed illustration of the geographical distributions of the patients is demonstrated in Fig. 1a and Supplementary Table 5. The sex distribution in PsA and non-PsA is slightly different, with approximately 58.1% (154 out of 265 PsA patients) of male patients with PsA and 63.4% (2344 out of 3696 patients) with non-PsA (Fig. 1b and Supplementary Table 4). In addition, our data suggest that patients aged between 30 and 60 are most prone to suffer from PsA. Among the PsA patients, 19.3%, 26.0%, and 31.3% are aged 30–39, 40–49, and 50–59, respectively (average age is 46.2), while patients aged between 20 and 60 are more susceptible to other types of psoriasis. Specifically, out of the 3696 patients with non-PsA, 18.7%, 21.4%, and 19.8% are adults aged 20–29, 30–39, and 40–49, respectively (average age is 40.3) (Fig. 1c and Supplementary Table 6). We also plotted the progression of different types of psoriasis to PsA (Fig. 1d). It is clear that an average of 22.8% of patients with psoriasis vulgaris progressed into PsA within approximately 1260 days. Despite the progression rates of the other three types of psoriasis along with time can be roughly calculated, they should be refined with more data available. With the clinical data collected, we designed three major steps to construct our prediction and analysis pipelines for PsA diagnosis, PsA risk assessment, and drug efficacy against psoriasis (Fig. 2). There are three major sections in our datasets, including patients’ clinical data, follow-up data, and treatment data. Three pipelines have been designed and implemented based on the three data sections (Fig. 2). Using the clinical data, we generated and assessed machine learning models for predicting patients with PsA and other types of psoriasis (i.e., non-PsA). Using the follow-up data, we developed a classification model based on machine learning methods for predicting the risk of progression from non-PsA to PsA within three years and analyzed and interpreted important features for the proposed risk model. In addition, we applied the Cox regression model^18^ to provide a global analysis of the risk tendency from other types of psoriasis to PsA and the key risk factors that affect PsA progression. Finally, with the medication data, we applied generalized additive models (GAMs)^19^ to investigate the drug efficacy in terms of psoriasis area and severity index (PASI)^20^ values. We also utilized the Kaplan-Meier (KM)^21^ model and the Cox regression models to analyze the efficacy difference in inhibiting PsA progression between methotrexate (MTX) and other types of drugs (i.e., non-MTX).

### Model construction and analysis for PsA prediction

Based on one randomly selected dataset from the 40 sub-training datasets, we made a preliminary analysis of features, including their distributions, abilities in differentiating PsA and other types of psoriasis, and correlations. The distributions of the clinical features are illustrated in Supplementary Figs. 1a, 2–8. We further plotted the receiver operating characteristic (ROC) curves and computed the area under ROC (AUC) values of each continuous numerical feature in our dataset (Supplementary Figs. 1b, 9–10 and Supplementary Table 7). It is shown that some features demonstrate strong abilities in differentiating PsA and non-PsA samples, such as variables related to PASI and body surface area (BSA)^22^, including Upper body (U)-PASI- Area (AUC: 0.634; 95% CI: 0.600–0.668), Torso (T)-PASI-Area (AUC: 0.620; 95% CI: 0.585–0.654), Lower body (L)-PASI-Area (AUC: 0.619; 95% CI: 0.585–0.653), BSA-double upper arms (AUC: 0.606; 95% CI: 0.575–0.637), BSA (AUC: 0.613; 95% CI: 0.579–0.648), and PASI (AUC: 0.612; 95% CI: 0.579–0.645). Age is another important potential diagnostic factor (AUC: 0.619; 95% CI: 0.587–0.651). The best feature for differentiating PsA from non-PsA is PASI-3-A, and its ROC curve and value distribution are described in Supplementary Fig. 1c. To examine the correlation between continuous numerical features in our dataset, we computed the Spearman correlation coefficients^23^ and visualized them in Supplementary Fig. 1d. It can be seen that Head&Neck (H)-PASI-A (Area) strongly correlates with some other PASI indexes and BSA indexes, such as BSA-double upper arms and BSA-double forearms. This preliminary analysis indicates that it is promising to develop a machine-learning model for discriminating between PsA and non-PsA using clinical features.

**Fig. 3:**
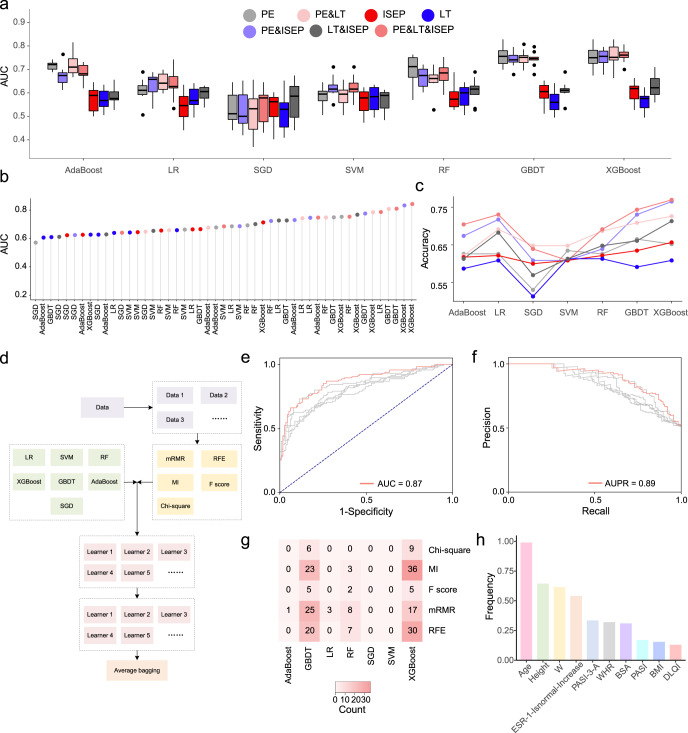
Performances of different models for predicting PsA. **a** The AUC values of different models trained using different categories of features and their combinations via the 10-fold cross-validation test based on the training datasets. The central line marks the median AUC value, and the bounds of the box mark the first and third quartiles. **b** The AUC values of models trained with different categories of features and their combinations using the test dataset. **c** The model accuracy values based on different categories of features and their combinations evaluated on the test dataset. **d** A flowchart of the proposed PsA prediction model. Comparison of ROC curves **e** and PR curves **f** on the test dataset. The red curve represents the prediction performance of the proposed PsA model. The grey curves represent the prediction performances of the five best-performing base classifiers. **g** A summary of the most significant feature strategies and machine learning methods of the proposed PsA diagnosis model. ‘Count’ demonstrates the times that the combination of the machine learning method and the feature selection technique has been selected in the final ensemble model across 40 datasets. **h** A summary of the most important features of the proposed PsA diagnosis model. *PE* physical examination. *LT* laboratory test. *ISEP* the indices for severity and extent of psoriasis. *W* waistline. *WHR* waist-hip ratio. *DLQI* dermatology life quality index. *ESR* erythrocyte sedimentation rate.

Based on the randomly selected sub-training dataset, we constructed several machine learning models and compared the prediction differences among different categories of features preliminarily. We first trained on different categories of features and compared the differences among these categories at first, aiming to explore the contribution of each feature category to the prediction performance. We calculated ROC curves and AUC and accuracy values on training and testing datasets (Fig. 3a–c). These results indicate the contributions of each category to the prediction performance. The best AUC values using PE features and ISEP features are 0.75 and 0.71 by extreme gradient boosting (XGBoost)^24^, and the best AUC using LT features is 0.66 by random forest (RF)^25^. While XGBoost achieved the highest AUC of 0.84 when combining all three categories of features. We therefore combined the features from three categories and applied feature selection strategies to further optimize the prediction performance.

For all 40 sub-training datasets, we compared the seven alternative classifiers based on five feature selection algorithms via a 10-fold cross-validation test (Supplementary Figs. 11–15). The ROC curves of the combinations of different machine learning models and feature selection approaches evaluated by the test dataset are demonstrated in Supplementary Figs. 16–20. It can be concluded that the best-performing approach is the XGBoost algorithm with the max-relevance and min-redundancy (mRMR)^26^ feature selection strategy, which achieved a higher AUC of 0.85 on the test dataset. To further improve the prediction performance, we ensembled all these base classifiers using the bagging strategy for the final prediction results (Fig. 3d and ‘Methods’). The ROC and precision-recall (PR) curves and corresponding AUC and area under PR (AUPR) values are demonstrated in Fig. 3e, f. The results based on the bagging strategy achieved an AUC of 0.87 and an AUPR value of 0.89, respectively, both of which are higher than those of any individual classifier of each sub-training dataset. Among the base classifiers, XGBoost and gradient boosting decision tree (GBDT)^27^ are the two most frequently selected classifiers because of their better prediction performances (Fig. 3g). We also found that mutual information (MI)^28^, recursive feature selection based on random forest (RFE-RF)^29^, and mRMR are the most effective feature selection strategies for these base classifiers (Fig. 3g). To explore the feature importance, we first computed the permutation importance (PI)^30^ for the ten most important features of each selected base classifier (Supplementary Figs. 21–28). Then we counted the frequency of these important features in all selected base classifiers and identified several most important features for PsA diagnosis, including age, height, waist-hip ratio (WHR), erythrocyte sedimentation rate, waist (W), PASI, BSA, BMI, and dermatology life quality index (DLQI) (Fig. 3h).

To further analyze the significant difference between groups of features, we performed a non-parametric Kruskal-Wallis H test (one-way non-parametric ANOVA) to test if the distributions of different feature values are the same. If different, we then applied a *post hoc* test to identify the distribution difference of different feature values. The results of the Kruskal-Wallis H test and *post hoc* test indicate that the attributes of some features have significant differences between PsA and non-PsA patients, including sex, age, and some laboratory tests (Supplementary Tables 8–9, and Supplementary Figs. 29–32).

### Prediction and analysis of the risk of PsA progression

**Fig. 4:**
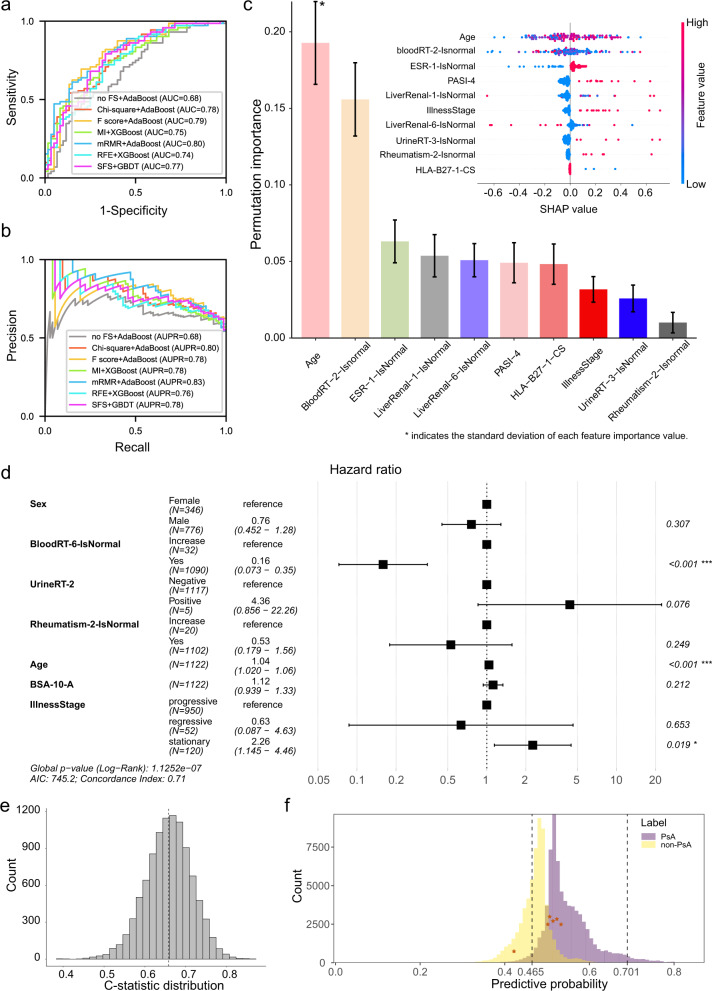
Performances of machine learning models and a Cox regression model for risk analysis of PsA progression. The ROC **a**, and PR **b** curves of the various machine-learning methods under different feature selection strategies using the Jackknife (i.e., leave-one-out) validation. The feature importance analysis **c** of the constructed model. Each bar indicates the average feature importance value, while the black line represents the standard deviation of each feature importance value. The forest plot **d** and C-statistic distribution **e** of the best multivariate Cox regression model. The *p*-value is obtained by the Log-Rank Test. **f** The predictive probability distribution based on bootstrapping validation test and the predictive results of the new PsA patients’ data. Each star point represents a PsA patient, and its corresponding abscissa value means its predictive probability. *BloodRT* blood routine test. *UrineRT* urine routine test. *WHR* waist-hip ratio.

To examine and compare the prediction performance of different models, we utilized the Jackknife (i.e., leave-one-out)^31^ validation test and plotted ROC and PR curves for the best-performing models with different feature selection strategies (Fig. 4a, b and Supplementary Fig. 33). We observed that adaptive boosting (AdaBoost)^32^ with mRMR achieved higher AUC with 20 to 30 features (Supplementary Fig. 33). When selecting 27 features using mRMR, the AUC and AUPR values outperformed other combinations (AUC of 0.80 and AUPR of 0.83). In this model, age, neutrophils (%) in the blood test, erythrocyte sedimentation rate, albumin (g/L), and alkaline phosphatase (ALP; U/L) in the liver and kidney function test are the five most informative features (Fig. 4c and Supplementary Table 2). Although previous studies have not indicated the predictive effect of albumin and ALP on PsA, we conducted a literature search and found a few studies demonstrating the correlation between albumin^33^/ALP^34,35^ and PsA. We also explored the risk factors of PsA progression using a Cox regression model^18^ based on all follow-up clinical data. The univariate Cox hazard analysis of different features is shown in Supplementary Table 10. We used Lasso regression^36^ with the “Cox” function based on 10-fold cross-validation to select the initial features for the Cox regression model and computed the corresponding hazard ratio (HR) for each feature (Supplementary Fig. 34). Based on the initially selected risk factors by Lasso regression, we further manually adjusted the selected features to obtain a better-performing model based on bootstrap sampling (Supplementary Table 11). We observed that the Cox regression model based on seven features (i.e., sex, platelet counts in the blood test, glucose (±) in urine test, C-reactive protein, illness stage, age, and BSA (hips)) achieved the highest C-statistic of 0.658 (CI: 0.542–0.767), indicating that the seven features have certain associations with PsA progression. The HRs of factors and C-statistic distribution are provided in Fig. 4d, e. Overall, the model developed based on machine learning (i.e., AdaBoost with mRMR) has a satisfactory performance in predicting psoriasis progression risk at the three-year time point, which is potential as a model for PsA risk prediction, and the Cox regression model provides a global analysis of the risk tendency from other types of psoriasis to PsA and the key factors related to PsA progression.

To further assess the proposed machine learning model, we conducted a prospective study based on six new PsA patients’ clinical data we collected. The six patients all developed from other types of psoriasis to PsA in a short period. We first obtained the predictive probability distribution using the 132 patients’ clinical data based on the bootstrapping validation strategy. The 95% CI of the predictive probability distribution of the positive samples is 0.465–0.701. Then, we tested the six PsA patients’ clinical data on the proposed predictive model and found that five of six (83.3%) predictive probabilities are in 0.465–0.701 (Fig. 4f), suggesting that the proposed model has the potential to perform an accurate risk prediction.

### Drug efficacy analysis of psoriasis

**Fig. 5:**
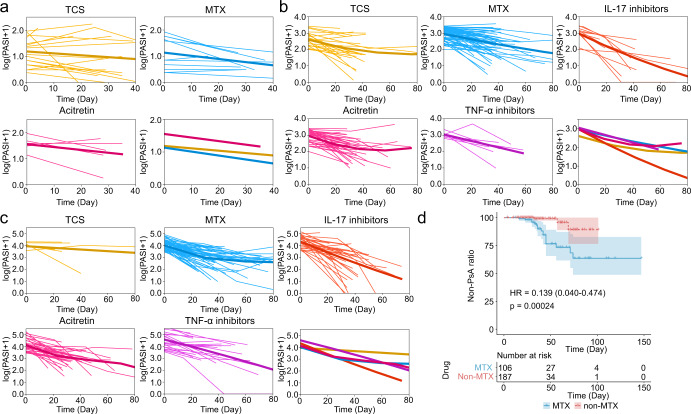
Efficacy comparison and analysis of different drugs for decreasing PASI values and inhibiting PsA progression. The logarithm of (PASI + 1) *vs*. time fitting curves of different drugs for those patients with mild psoriasis (PASI < 3) **a**, moderate psoriasis (3 ≤ PASI < 10) **b**, and severe psoriasis (PASI ≥ 10) **c**. The last subplots of **a**–**c** show the comparison of fitting curves of different drugs. **d** The Kaplan–Meier (KM) curve of the non-MTX drug group and MTX drug group. The *p*-value is obtained by the Log-Rank Test. *TCS* topical corticosteroid. *MTX* methotrexate. *IL-17 inhibitors* interleukin-17 inhibitors. *TNF-α inhibitors* tumor necrosis factor alpha inhibitors.

We first applied statistical approaches to explore the drug effects on psoriasis treatment in terms of PASI. Six types of drugs are covered in our dataset for psoriasis treatment, including MTX, topical corticosteroid (TCS), acitretin, tumor necrosis factor alpha (TNF-α) inhibitors, interleukin-17 (IL-17) inhibitors, and interleukin-23 (IL-23) inhibitors. The time-dependent log(PASI + 1) for each drug is described in Supplementary Fig. 35. Verifying these time-dependent PASI changes by a Mann-Whitney U test^37^, we found that there was no significant difference in PASI changes between the patients with PsA and non-PsA, regardless of the drug categories (TCS: *p*-value = 0.66, IL-17 inhibitors: *p*-value = 0.43, MTX: *p*-value = 0.72, TNF-α inhibitors: *p*-value = 0.62). The statistical *p*-values of acitretin and IL-23 inhibitors were not calculated due to the lack of PASI data from PsA patients. We therefore focused on the effects of different drug types on the PASI changes within all patients, including PsA and non-PsA. We further split our data into three subgroups, including mild (PASI < 3), moderate (3 ≤ PASI < 10), and severe (PASI ≥ 10) (‘Methods’) according to the PASI values at the patients’ first visits and fitted the GAMs for the time-dependent log(PASI + 1) for each subgroup (Fig. 5a–c). We noticed that acitretin, MTX, and TCS have similar effects on the changes of PASI given their similar gradients of GAM curves; IL-17 inhibitors have a better effect on reducing PASI values compared with other types of drugs in the moderate and severe subgroups, meaning that IL-17 inhibitors are more effective in the treatment of more severe psoriasis. Then, we collected the medication information from 17 new patients (Supplementary Fig. 36), also indicating that the IL-17 inhibitor is a more effective drug than the others tested.

In addition, we split the patients into two groups (MTX and non-MTX) to further analyze MTX’s effect on PsA progression (Methods). We utilized the KM method to draw the curves of PsA progression for the two groups (HR: 0.139, 95% CI: 0.040–0.474, Log-Rank test *p*-value = 0.00024) (Fig. 5d) and further made a subgroup analysis stratified by sex using Cox regression (male, HR: 0.129, 95% CI: 0.028–0.581, Log-Rank test *p*-value = 0.0016; female, HR: 0.174, 95% CI: 0.021–1.464, Log-Rank test *p*-value = 0.0701), suggesting that there is statistical significance in PsA progression between MTX drugs and non-MTX drugs and that MTX drugs have a weaker ability to prevent this progression compared with other drugs, especially for males. Note that the drug efficacy analysis was based on the currently available data from the hospital. Prospective studies and more clinical data are therefore required to further validate these models and findings.

## Discussion

In this study, we first developed a machine learning-based approach to predict PsA and demonstrated that several clinical factors have the potential to be contributing factors to the prediction model, as indicated by the AUC values, and revealed the relevance between different factors. Then we built and assessed a variety of powerful machine learning models and feature selection strategies to construct and select the best-performing PsA diagnosis model and analyzed the contributions of the important features to the final PsA prediction model. Several most important features for PsA diagnosis were identified, including age, height, waist-hip ratio (WHR), erythrocyte sedimentation rate, waist (W), PASI, BSA, BMI, and dermatology life quality index (DLQI). We also proposed a model based on AdaBoost and mRMR for predicting the probability of PsA progression within three years using the follow-up clinical data and analyzed the important factors for this model. In addition, we further conducted a statistical analysis of the key factors that affect PsA progression using the Cox regression model. Furthermore, we conducted a comprehensive analysis of drug efficacy in psoriasis treatment. Based on the data collected for this study, we identified that compared to other types of drugs in our dataset, IL-17 inhibitors achieve better efficacy in patients with more severe psoriasis (Fig. 5b, c), while MTX drugs have a lower ability in preventing the progression from non-PsA to PsA (Fig. 5d), providing with clinicians with useful insights in drug selection and prescription.

There are also some limitations in our analysis. First, as most of the psoriasis patients were not required for hospitalization, some factors cannot be monitored. For example, patients have different lifestyles and dietary habits, and some patients cannot take the medicine prescribed by their doctors or revisit doctors on time. Therefore, some of the clinical data may be noisy, leading to less accurate prediction performance. Second, the current data availability may limit our statistical analysis of drug efficacy in psoriasis treatment. For example, very few patients used IL-23 inhibitors in our dataset, so we cannot compare its efficacy in PASI changes with other drugs, even though IL-23 inhibitors have been reported as an effective drug for psoriasis treatment^38^. We also cannot analyze the effects of IL-17 inhibitors and TNF-α inhibitors on PASI changes in the mild subgroup with very limited data, as these drugs are more expensive and are therefore rarely prescribed by clinicians when the patients’ symptoms are mild. In addition, we identified that MTX has a weaker ability to prevent the progression from non-PsA to PsA compared with other drugs based on our current dataset with an HR of 0.139 and a 95% CI of 0.040–0.474 (Log-Rank test *p*-value = 0.00024). However, more data and experimental validations are therefore required to explore the efficacy of MTX drugs in PsA progression. Last, this study did not consider the ethnic and geographical factors in PsA and its progression risk prediction, or drug efficacy analysis, which should be further explored with available data from other ethnic groups in the future.

In our future work, we will collect more patients’ clinical data and imaging data, such as X-ray, ultrasonography, or magnetic resonance imaging (MRI) examination, and add imaging information into models to further improve the accuracy of PsA diagnosis. In addition, we will consider incorporating the biological information and develop a more appropriate downsampling strategy to enhance sample selection and improve the model performance. Recently, several studies explored the severe disease^39^, high-impact disease (PsAID ≥4)^40^, and Minimal disease activity (MDA)^41^ on patients with recent-onset PsA and identified several associated characteristics utilizing machine learning-based methods, providing more in-depth and prospective analysis of PsA patients with recent-onset PsA. In the further, we will endeavor to follow up on psoriasis and PsA patients, and record the scores and symptoms of relevant joints in detail, such as Ankylosing Spondylitis Disease Activity Score (ASDAS), the 28 joint disease activity scores (DAS28), Disease Activity index for Psoriatic Arthritis (DAPSA), high-impact disease (PsAID ≥4)^40^, and Minimal Disease Activity (MDA)^41^, *etc*., in order to extensively explore the possibility of developing a more comprehensive machine-learning model for PsA severity analysis and accurate stratification of the types of PsA patients in combination with such scores. In conclusion, our machine learning-based PsA and progression risk prediction models can assist clinicians in estimating whether a patient will have a probability of PsA progression in the near future and thereby can facilitate doctors to prescribe more reasonable medicine to manage the PsA risk. In addition, the contributing factors identified to the PsA diagnosis, PsA regression prediction, and drug efficacy can be exploited as useful guidance for clinicians and further validated in follow-up studies. We anticipate that the models developed in this study can facilitate the early diagnosis, prevention, and control of PsA, and assist clinicians with more effective drug prescriptions in line with the patient’s psoriasis condition.

## Methods

### Dataset preprocessing for PsA diagnosis model

The dataset, including clinical information of 3961 psoriasis patients, contained 10644 samples in total (written informed consent provided by all participants and ethics form approved by the Medical Ethics Committee of Xiangya Hospital Central South University; Ethics Number: 202005120). All psoriasis patients were diagnosed and confirmed by the dermatologists and rheumatologists at Xiangya Hospital. The clinical information documented in this dataset includes the general examination, blood test, urine test, PASI, BSA, liver and renal functions tests, DLQI, psoriasis type, therapeutic drugs, and lifestyle habits. To eliminate the missing information from the initial dataset, features with >20% missing values were removed, and the discrete features were transferred to the numerical presentation via the one-hot encoding scheme^42^. Abnormal values caused by the improper operation of the system were removed, and duplicate samples were deleted. Finally, we obtained a dataset including 578 PsA samples and 7459 non-PsA samples. First, we randomly selected 230 (115 PsA, 20% *vs*. 115 non-PsA) samples from the dataset as an independent test dataset and the remaining samples as the training dataset. To generate balanced training datasets, we used the remaining 463 PsA samples as the positive training dataset and randomly selected the same number of non-PsA samples as negative training datasets using the downsampling strategy. We repeated this random selection procedure 40 times and generated 40 sub-training datasets.

### Development of the PsA diagnosis model

We have designed several steps to construct, optimize and select the best machine-learning model for distinguishing PsA. First, we randomly selected a sub-training dataset to preliminarily analyze the feasibility of using machine learning methods to distinguish PsA. Using the selected sub-training dataset, we analyzed the distribution of each feature and the correlations between the continuous numerical features by calculating the Spearman correlation coefficients. We further analyzed the predictability of different features for PsA. Second, we clustered the clinical features into three categories, namely PE (Physical Examination), ISEP (Indices for Severity and Extend of Psoriasis), and LT (Laboratory Test) (Supplementary Table 1). To illustrate the contribution of each feature category to the prediction performance, we initially trained a series of machine-learning models using each feature category and all their possible combinations, respectively. In this study, we employed and compared seven widely applied classifiers and strategies, including logistic regression (LR)^43^, support vector machine (SVM)^44^ with radial basis function (RBF) kernel, stochastic gradient descent (SGD)^45^, AdaBoost^46^ based on decision tree (DT)^47^, RF^25^, XGBoost^24^, and gradient boosting decision tree (GBDT)^27^ as the base classifier. All the predictive models were constructed using the “*scikit-learn*” package (version 1.0.2) in Python (v3.7.6)^48^. To select optimal hyperparameters for these models initially, we conducted 10-fold cross-validation by iterating the regularization parameters. As a result, the LR (C = 1.0), SVM (C = 1.0), AdaBoost (n_estimators = 1000), RF (n_estimators = 100), XGBoost (n_estimators = 1000), and GBDT (n_estimators = 1000) were selected. To assess the prediction performance, we plotted the ROC^49^ curves and computed the AUC^49^ values in both 10-fold cross-validation and independent tests, using the “*sklearn.metrics*” function in Python. Given that the machine learning models trained based on the combinations of all three feature categories achieved the highest AUC values, we employed five feature selection algorithms, including Chi-squared statistics^50^, MI^28^, ANOVA F-statistic (F)^51^, mRMR^26^, and RFE-RF^29^, to further improve the prediction performance. These feature selection methods were also implemented using “*scikit-learn*” package. We selected different numbers of features (ranging from 5 to 100) and trained the models based on the 10-fold cross-validation test.

The whole analysis based on one sub-training dataset indicated it is possible to predict PsA using machine-learning methods (Fig. 3a, b). Therefore, we trained these machine learning algorithms with feature selection methods for all 40 sub-training datasets. For each sub-training dataset, we selected the five best models performed via a 10-fold cross-validation test as base classifiers. And we combined these based classifiers using the bagging strategy^52^ to construct the final ensemble model to further improve prediction performance. The feature importance was explored by the PI^30^ analysis using the “*permutation_importance*” function from “*sklearn.inspection*” package. Here, we permuted each feature ten times and computed the corresponding mean and standard deviation values to present the importance of the feature.

### Dataset preprocessing for the PsA risk prediction model

Our dataset includes 1122 patients’ follow-up data, and we chose three years as the timeframe for the prediction of psoriasis progression, given the data availability of different timeframes. The selected positive samples are those patients diagnosed with other types of psoriasis at their initial clinic visit and progressed to PsA within three years, and the negative samples are those patients with other types of psoriasis at their first clinic visit and who have not progressed to PsA to date within three years. As a result, we collected 132 patients, including 72 positive and 60 negative samples.

### Development of the PsA risk prediction model

With the 132 samples, seven machine-learning methods were applied, including SVM with RBF kernel, LR, SGD, AdaBoost, RF, XGBoost, and GBDT with six feature selection algorithms including Chi-Squared statistic, MI, ANOVA F-statistic, mRMR, forward feature selection^53^ based on RF, and RFE based on RF. As the size of the dataset is small, we applied the Jackknife (i.e., leave-one-out) validation test to evaluate these models. We analyzed the feature importance of the selected prediction model using PI^30^ by the “permutation_importance” function from the “*sklearn.inspection*” package. We also conducted the SHapley Additive exPlanation (SHAP)^54^ analysis using the “*shap*” package (version 0.39.0).

Furthermore, to use all follow-up clinical data as possible, we also utilized a Cox regression model^55^ to conduct an analysis of PsA progression and the related risk features using the “*survival*” (version 3.2.13) and “*survminer*” (version 0.4.9) libraries in R (version 3.6.3). We used Lasso regression^36^ with the “Cox” function based on the 10-fold cross-validation to select initial features for the Cox regression model and computed the corresponding HR for each feature. Based on the initially selected risk factors by Lasso regression, we further adjusted the selected features to obtain a better-performing model based on bootstrap resampling.

### Drug efficacy analysis of psoriasis

For analyzing the drug efficacy, we removed those patients who were prescribed different drugs during the treatment period in case the cross-use of different drugs affected our analysis. In addition, we selected those patients who followed up within 45 days after every visit, considering that patients usually need to have a recurring visit approximately once a month. Finally, we obtained the drug efficacy data from 347 patients.

In total, six main types of drugs for psoriasis treatment, including MTX, TCS, acitretin, TNF-α inhibitors, IL-17 inhibitors, and IL-23 inhibitors, were documented in our dataset. We therefore grouped the patients into six drug groups. We then split each drug group into two sets in terms of their psoriasis types: PsA and non-PsA, to determine whether there existed statistical significance in time-dependence PASI between PsA and non-PsA under the same drug treatment. Seeing that there is no significant difference in PASI changes between the patients with PsA and non-PsA by Mann-Whitney U test (TCS: *p*-value = 0.66, IL-17 inhibitors: *p*-value = 0.43, MTX: *p*-value = 0.72, TNF-α inhibitors: *p*-value = 0.62), we did not split patients into PsA patients and non-PsA patients to discuss the efficacy of different drugs on PASI. It is known that clinicians usually prescribe different drugs in terms of psoriasis severity. For example, suppose a patient does not have serious psoriasis. In that case, clinicians will prescribe common drugs in most cases, such as MTX and TCS, since these drugs are relatively cheap and enough to meet the patients’ needs; if a patient has serious psoriasis, more expensive biologic drugs will be prescribed, such as TNF-α or IL-17 inhibitors. Therefore, we further split each drug group into three subgroups according to the PASI values of patients at their initial visit: mild (PASI < 3), moderate (3 ≤ PASI < 10), and severe (PASI ≥ 10). We then fitted a GAM for each subgroup using the “*LinearGAM*” function from the “*pygam*” package (version 0.8.0) in Python, where the time points were presented on the X-axis and the logarithm values of (PASI + 1) were presented on the Y-axis, to compare and analyze the effects of different drugs on PASI.

In addition, we discovered that there are more MTX-treated patients developing from other types of psoriasis to PsA compared to those patients treated with other drugs (Supplementary Fig. 35). Therefore, we further compared the efficacy in inhibiting PsA progression between MTX and non-MTX. We first eliminated those patients who were diagnosed with PsA on their first visit to the hospital and then split the remaining patients into two groups: MTX (107 patients; the control group) and non-MTX (187 patients; the experimental group). Here, we utilized the KM^21^ method to visualize the differences and computed the HR. To further explore the differences in these findings between males and females, we performed a subgroup analysis stratified by sex using Cox regression.

### Statistical analysis

All multivariate PsA risk analyses were constructed using Cox regression methods, and the *p*-values from survival analyses were calculated using the Log-Rank test^56^. The performance of these risk models was assessed using Harrell’s C-statistics^57–59^. For 95% confidence intervals (CIs), we calculated from 2.5*th* and 97.5*th* quantiles of the bootstrap distribution by repeating 10000 times of bootstrap resampling^60^. The non-parametric Kruskal-Wallis H test (one-way non-parametric ANOVA) was applied to assess the significant difference among feature groups using the “*scikit-posthocs*” package (version 0.7.0). The statistical significance between PsA and non-PsA within the same drug treatment group was assessed by performing the Mann-Whitney U test. The *p*-value indicating the statistical significance between the MTX group and the non-MTX group was calculated using the Log-Rank test. All statistical tests were performed in R (version 3.6.3). We calculated the Spearman correlation coefficients^23^ to identify the correlations between the numerical variables in our dataset. The psoriasis progression event rate from non-PsA to PsA was calculated and plotted using the KM method. For each numerical variable, we plotted the ROC curve, calculated the confidence interval of sensitivity, extracted the AUC value, and annotated the threshold, using the “*pROC*”^61^ library (version 1.18.0) in R (version 3.6.3). We plotted both ROC curves and PR curves to compare the prediction performances between our proposed models with other machine learning models.

### Reporting summary

Further information on research design is available in the Nature Research Reporting Summary linked to this article.

## Supplementary information

Reporting Summary

Supplementary Materials

## Data Availability

The patients’ clinical data is available from the authors upon reasonable request.

## Code availability

All source code used to generate data in this manuscript is available on GitHub (https://github.com/joy50706/PSA-analysis).
