# Supplementary material for: Multi-modality data-driven analysis of diagnosis and treatment of psoriatic arthritis
Source: NPJ Digit Med. 2023 Feb 2;6:13. doi: 10.1038/s41746-023-00757-3 (PMC9895430; doi:10.1038/s41746-023-00757-3)
Supplement: Supplementary file 1 — Reporting Summary [file 41746_2023_757_MOESM1_ESM.pdf]

nature portfolio

Corresponding author(s): Hong Liu, Xiang Chen, Jiangning Song, Yehong Kuang and Qian Gao

Last updated by author(s): Jan 16, 2023

Reporting Summary

Nature Portfolio wishes to improve the reproducibility of the work that we publish. This form provides structure for consistency and transparency in reporting. For further information on Nature Portfolio policies, see our Editorial Policies and the Editorial Policy Checklist.

Statistics

For all statistical analyses, confirm that the following items are present in the figure legend, table legend, main text, or Methods section.

n/a	Confirmed	
☐	☒	The exact sample size (*n*) for each experimental group/condition, given as a discrete number and unit of measurement
☐	☒	A statement on whether measurements were taken from distinct samples or whether the same sample was measured repeatedly
☐	☒	The statistical test(s) used AND whether they are one- or two-sided<br>*Only common tests should be described solely by name; describe more complex techniques in the Methods section.*
☒	☐	A description of all covariates tested
☒	☐	A description of any assumptions or corrections, such as tests of normality and adjustment for multiple comparisons
☐	☒	A full description of the statistical parameters including central tendency (e.g. means) or other basic estimates (e.g. regression coefficient) AND variation (e.g. standard deviation) or associated estimates of uncertainty (e.g. confidence intervals)
☐	☒	For null hypothesis testing, the test statistic (e.g. *F*, *t*, *r*) with confidence intervals, effect sizes, degrees of freedom and *P* value noted<br>*Give P values as exact values whenever suitable.*
☒	☐	For Bayesian analysis, information on the choice of priors and Markov chain Monte Carlo settings
☒	☐	For hierarchical and complex designs, identification of the appropriate level for tests and full reporting of outcomes
☒	☐	Estimates of effect sizes (e.g. Cohen's *d*, Pearson's *r*), indicating how they were calculated

Our web collection on statistics for biologists contains articles on many of the points above.

Software and code

Policy information about availability of computer code

Data collection	The clinical records of psoriasis patients were all obtained from the Haitai Electronic Medical Record System V4.0 (Nanjing Haitai Medical Information System Co., Ltd) in Xiangya Hospital, China. All data had been granted permission from "Medical Ethics Committee of Xiangya Hospital Central South University" (Ethical number: 202005120), and were then used to construct the predictive models.
Data analysis	The predictive models were constructed using scikit-learn (v1.0.2) in Python (v3.7.6). The plots were drawn by R (v 3.6.3) and matplotlib (v 3.1.1). The Kaplan-Meier (KM) method, Cox regression models, and all statistical tests were performed in R (version 3.6.3). All source code used to generate data in this manuscript is available on GitHub (https://github.com/joy50706/PSA-analysis). The non-parametric Kruskal-Wallis H test (one-way non-parametric ANOVA) was applied to assess the significant difference among feature groups using the scikit-posthocs package (version 0.7.0). . For each numerical variable, we plotted the ROC curve, calculated the confidence interval of sensitivity, extracted AUC values, and annotated the threshold, using the pROC62 library in R (version 3.6.3).

For manuscripts utilizing custom algorithms or software that are central to the research but not yet described in published literature, software must be made available to editors and reviewers. We strongly encourage code deposition in a community repository (e.g. GitHub). See the Nature Portfolio guidelines for submitting code & software for further information.

Data

Policy information about availability of data

All manuscripts must include a data availability statement. This statement should provide the following information, where applicable:

- Accession codes, unique identifiers, or web links for publicly available datasets
- A description of any restrictions on data availability
- For clinical datasets or third party data, please ensure that the statement adheres to our policy

The patients' clinical data are available from the authors upon reasonable request.

Human research participants

Policy information about studies involving human research participants and Sex and Gender in Research.

Reporting on sex and gender	Of these 3961 patients, approximately 58.1% (154 out of 265 PsA patients) are male with PsA and 63.4% (2344 out of 3696 patients) are with non-PsA.
Population characteristics	Of these 3961 patients, 3241 (81.8%) are from Hunan Province, China, and 267 (6.7%) are from Jiangxi Province. The numbers of patients from other provinces are all less than 50. Among the PsA patients, 19.3%, 26.0%, and 31.3% are aged 30-39, 40-49, and 50-59, respectively (average age is 46.2), while patients aged between 20 and 60 are more susceptible to other types of psoriasis. Specifically, out of the 3696 patients with non-PsA, 18.7%, 21.4%, and 19.8% are adults aged 20-29, 30-39, and 40-49, respectively (the average age is 40.3)
Recruitment	3961 patients who were diagnosed with psoriasis and treated at Xiangya Hospital of Central South University, China, from January 2017 to August 2021, were collected. All clinical records of patients from Haitai Electronic Medical Record System V4.0 (Nanjing Haitai Medical Information System Co., Ltd) in Xiangya Hospital, are genuine. Patients with a huge amount of missing data were excluded, so self-selection bias or other biases regarding the selected patient's information didn't exist.
Ethics oversight	Medical Ethics Committee of Xiangya Hospital Central South University (Ethical number: 202005120).

Note that full information on the approval of the study protocol must also be provided in the manuscript.

Field-specific reporting

Please select the one below that is the best fit for your research. If you are not sure, read the appropriate sections before making your selection.

☒ Life sciences ☐ Behavioural & social sciences ☐ Ecological, evolutionary & environmental sciences

For a reference copy of the document with all sections, see nature.com/documents/nr-reporting-summary-flat.pdf

Life sciences study design

All studies must disclose on these points even when the disclosure is negative.

Sample size	This study selected full clinical record data of a large patient cohort, including 3961 patients who were diagnosed with psoriasis and treated at Xiangya Hospital of Central South University, China, from January 2017 to August 2021. The clinical information documented in this dataset includes general examination, blood test, urine test, PASI, BSA, liver and renal functions tests, DLQI, psoriasis type, therapeutic drugs, and their lifestyle habits. In this dataset, 1122 patients' follow-up data were included.
Data exclusions	To eliminate the missing information from the initial dataset, features with >20% missing values were removed. Abnormal values caused by the improper operation of the system were removed, and duplicate samples were deleted. For analyzing the specific drug efficacy, those patients who were prescribed different drugs during the treatment period were removed in case the cross-use of different drugs affected our analysis.
Replication	Seven widely applied classifiers and strategies, including logistic regression (LR), support vector machine (SVM) with radial basis function (RBF) kernel, stochastic gradient descent (SGD), adaptive boosting (AdaBoost) based on decision tree (DT), random forest (RF), extreme gradient boosting (XGBoost), and gradient boosting decision tree (GBDT) were employed and compared. For the PsA prediction model construction, to assess the prediction performance, the 10-fold cross-validation test was applied. The average AUC values and average AUPR values were computed to evaluate the predictive performance. For the PsA progression prediction model, the leave-one-out cross-validation test was applied and the corresponding AUC value and AUPR value were computed.
Randomization	For PsA prediction model, a dataset including 578 PsA samples and 7459 non-PsA samples was obtained. 230 (115 PsA, 20% vs. 115 non-PsA) samples were randomly selected from the dataset as an independent test dataset, and the remaining samples were used as the training dataset.
Blinding	The conventional blinding was not relevant to this study because this study didn't include any interventions.

Reporting for specific materials, systems and methods

We require information from authors about some types of materials, experimental systems and methods used in many studies. Here, indicate whether each material, system or method listed is relevant to your study. If you are not sure if a list item applies to your research, read the appropriate section before selecting a response.

Materials & experimental systems

n/a	Involved in the study
☒	☐ Antibodies
☒	☐ Eukaryotic cell lines
☒	☐ Palaeontology and archaeology
☒	☐ Animals and other organisms
☒	☐ Clinical data
☒	☐ Dual use research of concern

Methods

n/a	Involved in the study
☒	☐ ChIP-seq
☒	☐ Flow cytometry
☒	☐ MRI-based neuroimaging
