# Supplementary material for: Multi-modality data-driven analysis of diagnosis and treatment of psoriatic arthritis
Source: NPJ Digit Med. 2023 Feb 2;6:13. doi: 10.1038/s41746-023-00757-3 (PMC9895430; doi:10.1038/s41746-023-00757-3)

Supplementary Information
Supplement to: Multi-modality data-driven analysis of diagnosis and treatment of psoriatic arthritis: a retrospective study

Jing Xu[1,2,3,4,†], Jiarui Ou[1,2,5,6,†], Chen Li[3,†], Zheng Zhu[7], Jian Li[8], Hailun Zhang[9], Junchen Chen[1,2,5,6], Bin Yi[10], Wu Zhu[1,2,5,6], Weiru Zhang[11,12], Guanxiong Zhang[1,2,5,6], Qian Gao[10,1,2,5,6,*], Yehong Kuang[1,2,5,6,*], Jiangning Song[3,4,*], Xiang Chen[1,2,5,6,*], and Hong Liu[1,2,5,6,*]

[1]Department of Dermatology, Xiangya Hospital, Central South University, Changsha, Hunan 410008, China.
[2]National Engineering Research Center of Personalized Diagnostic and Therapeutic Technology, Changsha, Hunan 410008, China.
[3]Monash Biomedicine Discovery Institute and Department of Biochemistry and Molecular Biology, Monash University, Melbourne, VIC 3800, Australia.
[4]Monash Data Futures Institute and Department of Biochemistry and Molecular Biology, Monash University, Melbourne, VIC 3800, Australia.
[5]Hunan Key Laboratory of Skin Cancer and Psoriasis, Changsha, Hunan 410008, China.
[6]Hunan Engineering Research Center of Skin Health and Disease, Changsha, Hunan 410008, China.
[7]Department of Medicine, Brigham and Women's Hospital, Harvard Medical School, Boston, MA 02115, USA.
[8]Monash Biomedicine Discovery Institute and Department of Microbiology, Monash University, Melbourne, VIC 3800, Australia.
[9]Department of Research and Development, Beijing GAP Biotechnology Co., Ltd, Beijing, 102600, China.
[10]Department of Clinical Laboratory, Xiangya Hospital, Central South University, Changsha, Hunan 410008, China.
[11]Department of Rheumatology and Immunology, Xiangya Hospital, Central South University, Changsha, Hunan 410008, China.
[12]Department of General Medicine, Xiangya Hospital, Central South University, Changsha, Hunan 410008, China.

[†]These authors contributed equally to this work.
[*]To whom correspondence should be addressed. Hong Liu: hongliu1014@csu.edu.cn; Xiang Chen: chenxiangck@126.com; Jiangning Song: Jiangning.Song@monash.edu; Yehong Kuang: yh_927@126.com; and Qian Gao: gaoqian@csu.edu.cn.

Supplementary Tables

Supplementary Table 1. **A detailed list of clinical features of psoriasis patients in the dataset.**

Category		Factors
physical examination (PE)		Sex, Age, Height, Weight, W, H, WHR, BMI, SBP, DBP, Onset Seasons
Indices for severity and extent of psoriasis (ISEP)		PASI, BSA, PASI-1-A, PASI-2-A, PASI-3-A, PASI-4-A, PASI-4-E, PASI-4-D, PASI-4-I, BSA-1-A, BSA-2-A, BSA-3-A, BSA-4-A, BSA-5-A, BSA-6-A, BSA-7-A, BSA-8-A, BSA-9-A, BSA-10-A, BSA-11-A, BSA-12-A, BSA-13-A, Illness Stage, DLQI
Laboratory test (LT)	bloodRT	bloodRT-1-IsNormal, bloodRT-1-ClinicalSignificant, bloodRT-2-IsNormal, bloodRT-2-ClinicalSignificant, bloodRT-3-IsNormal, bloodRT-3-ClinicalSignificant, bloodRT-4-IsNormal, bloodRT-4-ClinicalSignificant, bloodRT-5-IsNormal, bloodRT-5-ClinicalSignificant, bloodRT-6-IsNormal, bloodRT-6-ClinicalSignificant
	UrineRT	UrineRT-1, UrineRT-1-IsNormal, UrineRT-1-ClinicalSignificant, UrineRT-2, UrineRT-2-IsNormal, UrineRT-2-ClinicalSignificant, UrineRT-3, UrineRT-3-IsNormal, UrineRT-3-ClinicalSignificant, UrineRT-4, UrineRT-4-IsNormal, UrineRT-4-ClinicalSignificant
	LiverRenal	LiverRenal-1-IsNormal, LiverRenal-1-ClinicalSignificant, LiverRenal-2-IsNormal, LiverRenal-2-ClinicalSignificant, LiverRenal-3-IsNormal, LiverRenal-3-ClinicalSignificant, LiverRenal-4-IsNormal, LiverRenal-4-ClinicalSignificant, LiverRenal-5-IsNormal, LiverRenal-5-ClinicalSignificant, LiverRenal-6-IsNormal, LiverRenal-7-IsNormal, LiverRenal-8-IsNormal, LiverRenal-8-ClinicalSignificant, LiverRenal-9-IsNormal, LiverRenal-9-ClinicalSignificant
	BloodLipid	BloodLipid-1-IsNormal, BloodLipid-1-ClinicalSignificant, BloodLipid-2-IsNormal, BloodLipid-2-ClinicalSignificant, BloodLipid-3-IsNormal, BloodLipid-3-ClinicalSignificant, BloodLipid-4-IsNormal, BloodLipid-4-ClinicalSignificant
	BloodElectrolyte	BloodElectrolyte-1-IsNormal, BloodElectrolyte-1-ClinicalSignificant, BloodElectrolyte-2-IsNormal, BloodElectrolyte-2-ClinicalSignificant, BloodElectrolyte-3-IsNormal, BloodElectrolyte-3-ClinicalSignificant
	Others	25-OH-D3-1-IsNormal, 25-OH-D3-1-ClinicalSignificant, ESR-1-IsNormal, ESR-1-ClinicalSignificant, Rheumatism-1-IsNormal, Rheumatism-2-IsNormal, Rheumatism-2-ClinicalSignificant, Rheumatism-3-IsNormal, Rheumatism-3-ClinicalSignificant, HLA-B27-1, HLA-B27-1-IsNormal, HLA-B27-1-ClinicalSignificant
Drug		Acitretin, MTX, corticosteroids, IL-17 inhibitors, IL-23 inhibitors, TNF-α inhibitors

Supplementary Table 2. Features documented in the dataset and their full terms.

Feature abbreviation	Feature description
W	Waistline
H	Hipline
WHR	Waist-Hip Ratio
BMI	Body Mass Index
SBP	Systolic Blood Pressure
DBP	Diastolic Blood Pressure
PASI-1-A	Head & Neck (H) - PASI - A (Area)
PASI-1-E	Head & Neck (H) - PASI - E (Erythema)
PASI-1-D	Head & Neck (H) - PASI - D (Desquamation)
PASI-1-I	Head & Neck (H) - PASI - I (Infiltration)
PASI-2-A	Torso (T) - PASI - A (Area)
PASI-2-E	Torso (T) - PASI - E (Erythema)
PASI-2-D	Torso (T) - PASI - D (Desquamation)
PASI-2-I	Torso (T) - PASI - I (Infiltration)
PASI-3-A	Upper body (U) - PASI - A (Area)
PASI-3-E	Upper body (U) - PASI - E (Erythema)
PASI-3-D	Upper body (U) - PASI - D (Desquamation)
PASI-3-I	Upper body (U) - I (Infiltration)
PASI-4-A	Lower body (L) - PASI - A (Area)
PASI-4-E	Lower body (L) - PASI - E (Erythema)
PASI-4-D	Lower body (L) - PASI - D (Desquamation)
PASI-4-I	Lower body (L) - I (Infiltration)
BSA-1	BSA (head)
BSA-2	BSA (face)
BSA-3	BSA (neck)
BSA-4	BSA (double upper arms)
BSA-5	BSA (double forearms)
BSA-6	BSA (double hands)
BSA-7	BSA (front torso)
BSA-8	BSA (back torso)
BSA-9	BSA (perineum)
BSA-10	BSA (hips)
BSA-11	BSA (double thighs)
BSA-12	BSA (double calves)
BSA-13	BSA (double feet)
bloodRT-1	Full blood count (FBC) - White cell count (10^9/L)
bloodRT-2	FBC - Neutrophils (%)
bloodRT-3	FBC - Lymphocytes (%)
bloodRT-4	FBC - Red cell count (10^12/L)
bloodRT-5	FBC - Haemoglobin (g/L)
bloodRT-6	FBC - Platelet count (10^9/L)

UrineRT-1	Urine test (UT) - Protein (-/+)
UrineRT-2	UT - Glucose (-/+)
UrineRT-3	UT - White blood cells (-/+)
UrineRT-4	UT - Red blood cells (-/+)
LiverRenal-1	Liver and kidney function (LKF) - Albumin (g/L)
LiverRenal-2	LKF - Total bilirubin (TBil; μmol/L)
LiverRenal-3	LKF - Direct bilirubin (Dbil; μmol/L)
LiverRenal-4	LKF - Alanine aminotransferase (ALT; u/L)
LiverRenal-5	LKF - Aspartate aminotransferase (AST; u/L)
LiverRenal-6	LKF - Alkaline phosphatase (ALP; U/L)
LiverRenal-7	LKF - γ-glutamyl transpeptidase (GGT; U/L)
LiverRenal-8	LKF - Blood urea nitrogen (BUN; mmol/L)
LiverRenal-9	LKF - Creatinine (CREA; μmol/L)
LiverRenal-10	LKF - Uric Acid (UA; μmol/L)
BloodLipid-1	cholesterol
BloodLipid-2	Triglycerides
BloodLipid-3	High density lipoprotein
BloodLipid-4	Low-density lipoprotein
BloodElectrolyte-1	Electrolyte - Sodium
BloodElectrolyte-2	Electrolyte-Calcium
BloodElectrolyte-3	Electrolyte - Potassium
25-OH-D3	25-hydroxy vitamin D3
ESR	erythrocyte sedimentation Rate
Rheumatism-1	Anti "0" experiment
Rheumatism-2	C-reactive protein, CRP
Rheumatism-3	Rheumatoid factor
HLA-B27	HLA-B27 allotype

Supplementary Table 3. Distributions of continuous numerical features between patients with PsA and non-PsA.

Item/Feature	Overall	PsA	Non-PsA	Kruskal–Wallis test *p*-value
Number of patients	3961	265	3696	
Age (mean (SD))	40.65 (15.95)	46.23 (12.46)	40.26 (16.10)	<0.001
PASI (mean (SD))	8.61 (8.09)	9.16 (8.92)	8.57 (8.02)	0.276
BSA (mean (SD))	12.93 (15.93)	14.88 (18.54)	12.79 (15.72)	0.059
Height (mean (SD))	164.50 (9.74)	163.76 (7.58)	164.54 (9.84)	0.6
Weight (mean (SD))	64.05 (14.58)	64.27 (12.81)	64.04 (14.66)	0.832
W (mean (SD))	83.58 (11.60)	84.89 (10.18)	83.51 (11.66)	0.66
H (mean (SD))	92.95 (8.92)	93.29 (7.15)	92.94 (9.00)	0.532
WHR (mean (SD))	0.90 (0.08)	0.91 (0.07)	0.89 (0.08)	0.181
BMI (mean (SD))	23.46 (4.21)	23.88 (4.09)	23.44 (4.21)	0.736
SBP (mean (SD))	123.53 (13.51)	125.52 (13.37)	123.43 (13.51)	0.074
DBP (mean (SD))	75.90 (9.15)	76.98 (8.19)	75.84 (9.20)	0.022
PASI-1-A (mean (SD))	10.72 (12.13)	11.33 (12.14)	10.67 (12.13)	0.422
PASI-2-A (mean (SD))	15.80 (19.91)	16.22 (20.81)	15.77 (19.84)	0.741
PASI-3-A (mean (SD))	10.29 (13.95)	10.86 (16.30)	10.25 (13.76)	0.527
PASI-4-A (mean (SD))	18.38 (19.93)	19.59 (22.86)	18.29 (19.69)	0.337
PASI-4-E (mean (SD))	2.10 (0.89)	2.25 (0.92)	2.09 (0.89)	0.01
PASI-4-D (mean (SD))	1.78 (0.97)	1.82 (0.96)	1.77 (0.97)	0.464
PASI-4-I (mean (SD))	1.70 (0.89)	1.78 (0.91)	1.69 (0.89)	0.143
BSA-1-A (mean (SD))	0.70 (0.84)	0.82 (0.83)	0.69 (0.84)	0.018
BSA-2-A (mean (SD))	0.12 (0.34)	0.12 (0.37)	0.12 (0.34)	0.847
BSA-3-A (mean (SD))	0.08 (0.30)	0.09 (0.37)	0.08 (0.30)	0.478
BSA-4-A (mean (SD))	0.69 (1.17)	0.81 (1.43)	0.68 (1.15)	0.085
BSA-5-A (mean (SD))	0.74 (1.10)	0.83 (1.29)	0.73 (1.08)	0.143
BSA-6-A (mean (SD))	0.22 (0.63)	0.32 (0.81)	0.21 (0.61)	0.004
BSA-7-A (mean (SD))	1.25 (2.30)	1.31 (2.47)	1.24 (2.29)	0.623
BSA-8-A (mean (SD))	1.93 (2.93)	2.21 (3.20)	1.91 (2.91)	0.115
BSA-9-A (mean (SD))	0.02 (0.11)	0.02 (0.11)	0.02 (0.11)	0.783
BSA-10-A (mean (SD))	0.71 (1.16)	0.96 (1.37)	0.69 (1.14)	<0.001
BSA-11-A (mean (SD))	2.30 (4.31)	2.74 (5.02)	2.26 (4.26)	0.087
BSA-12-A (mean (SD))	2.47 (3.19)	2.72 (3.54)	2.45 (3.16)	0.195
BSA-13-A (mean (SD))	0.19 (0.73)	0.24 (0.79)	0.18 (0.72)	0.245
DLQI (mean (SD))	6.29 (4.76)	7.15 (5.17)	6.23 (4.72)	0.003

Supplementary Table 4. Distributions of categorical features between patients with PsA and non-PsA.

Item/Feature	Level	Overall	PsA	Non-PsA	Fisher's exact test *p*-value
Number of patients		3961	265	3696	
Sex (%)	Female	1463 (36.9)	111 (41.9)	1352 (36.6)	0.096
	Male	2498 (63.1)	154 (58.1)	2344 (63.4)	
bloodRT-1-IsNormal (%)	Decrease	7 (0.2)	0 (0.0)	7 (0.2)	0.004
	Increase	158 (4.1)	21 (8.0)	137 (3.8)	
	Yes	3655 (95.7)	240 (92.0)	3415 (96.0)	
bloodRT-1-ClinicalSignificant (%)	No	3812 (99.8)	259 (99.2)	3553 (99.8)	0.181
	Yes	8 (0.2)	2 (0.8)	6 (0.2)	
bloodRT-2-IsNormal (%)	Decrease	13 (0.3)	1 (0.4)	12 (0.3)	0.001
	Increase	82 (2.1)	14 (5.4)	68 (1.9)	
	Yes	3725 (97.5)	246 (94.3)	3479 (97.8)	
bloodRT-2-ClinicalSignificant (%)	No	3813 (99.8)	261 (100.0)	3552 (99.8)	1
	Yes	7 (0.2)	0 (0.0)	7 (0.2)	
bloodRT-3-IsNormal (%)	Decrease	156 (4.1)	21 (8.0)	135 (3.8)	0.002
	Increase	13 (0.3)	2 (0.8)	11 (0.3)	
	Yes	3651 (95.6)	238 (91.2)	3413 (95.9)	
bloodRT-3-ClinicalSignificant (%)	No	3814 (99.8)	261 (100.0)	3553 (99.8)	1
	Yes	6 (0.2)	0 (0.0)	6 (0.2)	
bloodRT-4-IsNormal (%)	Decrease	53 (1.4)	3 (1.1)	50 (1.4)	0.653
	Increase	70 (1.8)	3 (1.1)	67 (1.9)	
	Yes	3697 (96.8)	255 (97.7)	3442 (96.7)	
bloodRT-4-ClinicalSignificant (%)	No	3812 (99.8)	259 (99.2)	3553 (99.8)	0.181
	Yes	8 (0.2)	2 (0.8)	6 (0.2)	
bloodRT-5-IsNormal (%)	Decrease	73 (1.9)	8 (3.1)	65 (1.8)	0.286
	Increase	42 (1.1)	4 (1.5)	38 (1.1)	
	Yes	3705 (97.0)	249 (95.4)	3456 (97.1)	
bloodRT-5-ClinicalSignificant (%)	No	3808 (99.7)	260 (99.6)	3548 (99.7)	1
	Yes	12 (0.3)	1 (0.4)	11 (0.3)	
bloodRT-6-IsNormal (%)	Decrease	22 (0.6)	0 (0.0)	22 (0.6)	<0.001
	Increase	89 (2.3)	21 (8.0)	68 (1.9)	
	Yes	3709 (97.1)	240 (92.0)	3469 (97.5)	
bloodRT-6-ClinicalSignificant (%)	No	3818 (99.9)	261 (100.0)	3557 (99.9)	1
	Yes	2 (0.1)	0 (0.0)	2 (0.1)	
UrineRT-1 (%)	Negative	3802 (99.5)	260 (99.6)	3542 (99.5)	1
	Positive	18 (0.5)	1 (0.4)	17 (0.5)	
UrineRT-1-IsNormal (%)	No	9 (0.2)	0 (0.0)	9 (0.3)	0.879
	Yes	3811 (99.8)	261 (100.0)	3550 (99.7)	
UrineRT-1-ClinicalSignificant (%)	No	3817 (99.9)	261 (100.0)	3556 (99.9)	1
	Yes	3 (0.1)	0 (0.0)	3 (0.1)	
UrineRT-2 (%)	Negative	3809 (99.7)	257 (98.5)	3552 (99.8)	0.001
	Positive	11 (0.3)	4 (1.5)	7 (0.2)	

UrineRT-2-IsNormal (%)	No	6 (0.2)	2 (0.8)	4 (0.1)	0.078
	Yes	3814 (99.8)	259 (99.2)	3555 (99.9)	
UrineRT-2-ClinicalSignificant (%)	No	3818 (99.9)	260 (99.6)	3558 (100.0)	0.308
	Yes	2 (0.1)	1 (0.4)	1 (0.0)	
UrineRT-3 (%)	Negative	3655 (95.7)	242 (92.7)	3413 (95.9)	0.023
	Positive	165 (4.3)	19 (7.3)	146 (4.1)	
UrineRT-3-IsNormal (%)	No	70 (1.8)	8 (3.1)	62 (1.7)	0.194
	Yes	3750 (98.2)	253 (96.9)	3497 (98.3)	
UrineRT-3-ClinicalSignificant (%)	No	3817 (99.9)	261 (100.0)	3556 (99.9)	1
	Yes	3 (0.1)	0 (0.0)	3 (0.1)	
UrineRT-4 (%)	Negative	3751 (98.2)	257 (98.5)	3494 (98.2)	0.918
	Positive	69 (1.8)	4 (1.5)	65 (1.8)	
UrineRT-4-IsNormal (%)	No	20 (0.5)	2 (0.8)	18 (0.5)	0.906
	Yes	3800 (99.5)	259 (99.2)	3541 (99.5)	
UrineRT-4-ClinicalSignificant (%)	No	3818 (99.9)	260 (99.6)	3558 (100.0)	0.308
	Yes	2 (0.1)	1 (0.4)	1 (0.0)	
LiverRenal-1-IsNormal (%)	Decrease	78 (2.1)	13 (5.0)	65 (1.8)	<0.001
	Increase	9 (0.2)	4 (1.5)	5 (0.1)	
	Yes	3688 (97.7)	243 (93.5)	3445 (98.0)	
LiverRenal-1-ClinicalSignificant (%)	No	3756 (99.5)	258 (99.2)	3498 (99.5)	0.862
	Yes	19 (0.5)	2 (0.8)	17 (0.5)	
LiverRenal-2-IsNormal (%)	Decrease	1 (0.0)	1 (0.4)	0 (0.0)	0.001
	Increase	116 (3.0)	10 (3.8)	106 (3.0)	
	Yes	3703 (96.9)	250 (95.8)	3453 (97.0)	
LiverRenal-2-ClinicalSignificant (%)	No	3817 (99.9)	260 (99.6)	3557 (99.9)	0.499
	Yes	3 (0.1)	1 (0.4)	2 (0.1)	
LiverRenal-3-IsNormal (%)	Decrease	1 (0.0)	0 (0.0)	1 (0.0)	0.904
	Increase	158 (4.2)	12 (4.6)	146 (4.2)	
	Yes	3616 (95.8)	248 (95.4)	3368 (95.8)	
LiverRenal-3-ClinicalSignificant (%)	No	3771 (99.9)	260 (100.0)	3511 (99.9)	1
	Yes	4 (0.1)	0 (0.0)	4 (0.1)	
LiverRenal-4-IsNormal (%)	Decrease	18 (0.5)	1 (0.4)	17 (0.5)	0.348
	Increase	167 (4.4)	16 (6.1)	151 (4.2)	
	Yes	3635 (95.2)	244 (93.5)	3391 (95.3)	
LiverRenal-4-ClinicalSignificant (%)	No	3815 (99.9)	260 (99.6)	3555 (99.9)	0.779
	Yes	5 (0.1)	1 (0.4)	4 (0.1)	
LiverRenal-5-IsNormal (%)	Decrease	20 (0.5)	5 (1.9)	15 (0.4)	0.002
	Increase	119 (3.1)	12 (4.6)	107 (3.0)	
	Yes	3681 (96.4)	244 (93.5)	3437 (96.6)	
LiverRenal-5-ClinicalSignificant (%)	No	3814 (99.8)	260 (99.6)	3554 (99.9)	0.884
	Yes	6 (0.2)	1 (0.4)	5 (0.1)	
LiverRenal-6-IsNormal (%)	Increase	52 (1.4)	3 (1.2)	49 (1.4)	0.964
	Yes	3723 (98.6)	257 (98.8)	3466 (98.6)	

LiverRenal-7-IsNormal (%)	Increase	24 (0.6)	1 (0.4)	23 (0.7)	0.902
	Yes	3751 (99.4)	259 (99.6)	3492 (99.3)	
LiverRenal-8-IsNormal (%)	Decrease	94 (2.5)	8 (3.1)	86 (2.4)	0.42
	Increase	18 (0.5)	0 (0.0)	18 (0.5)	
	Yes	3708 (97.1)	253 (96.9)	3455 (97.1)	
LiverRenal-8-ClinicalSignificant (%)	No	3819 (100.0)	261 (100.0)	3558 (100.0)	1
	Yes	1 (0.0)	0 (0.0)	1 (0.0)	
LiverRenal-9-IsNormal (%)	Decrease	5 (0.1)	0 (0.0)	5 (0.1)	0.338
	Increase	31 (0.8)	4 (1.5)	27 (0.8)	
	Yes	3784 (99.1)	257 (98.5)	3527 (99.1)	
LiverRenal-9-ClinicalSignificant (%)	No	3819 (100.0)	260 (99.6)	3559 (100.0)	0.087
	Yes	1 (0.0)	1 (0.4)	0 (0.0)	
LiverRenal-10-IsNormal (%)	Decrease	17 (0.4)	4 (1.5)	13 (0.4)	0.012
	Increase	283 (7.4)	24 (9.2)	259 (7.3)	
	Yes	3520 (92.1)	233 (89.3)	3287 (92.4)	
LiverRenal-10-ClinicalSignificant (%)	No	3812 (99.8)	261 (100.0)	3551 (99.8)	0.948
	Yes	8 (0.2)	0 (0.0)	8 (0.2)	
BloodLipid-1-IsNormal (%)	Decrease	3 (0.1)	0 (0.0)	3 (0.1)	0.128
	Increase	467 (12.2)	42 (16.1)	425 (11.9)	
	Yes	3350 (87.7)	219 (83.9)	3131 (88.0)	
BloodLipid-1-ClinicalSignificant (%)	No	3814 (99.8)	261 (100.0)	3553 (99.8)	1
	Yes	6 (0.2)	0 (0.0)	6 (0.2)	
BloodLipid-2-IsNormal (%)	Decrease	1 (0.0)	1 (0.4)	0 (0.0)	<0.001
	Increase	461 (12.1)	43 (16.5)	418 (11.7)	
	Yes	3358 (87.9)	217 (83.1)	3141 (88.3)	
BloodLipid-2-ClinicalSignificant (%)	No	3814 (99.8)	260 (99.6)	3554 (99.9)	0.884
	Yes	6 (0.2)	1 (0.4)	5 (0.1)	
BloodLipid-3-IsNormal (%)	Decrease	295 (7.7)	27 (10.3)	268 (7.5)	0.258
	Increase	155 (4.1)	10 (3.8)	145 (4.1)	
	Yes	3370 (88.2)	224 (85.8)	3146 (88.4)	
BloodLipid-3-ClinicalSignificant (%)	No	3809 (99.7)	260 (99.6)	3549 (99.7)	1
	Yes	11 (0.3)	1 (0.4)	10 (0.3)	
BloodLipid-4-IsNormal (%)	Decrease	14 (0.4)	1 (0.4)	13 (0.4)	0.031
	Increase	512 (13.4)	49 (18.8)	463 (13.0)	
	Yes	3294 (86.2)	211 (80.8)	3083 (86.6)	
BloodLipid-4-ClinicalSignificant (%)	No	3812 (99.8)	261 (100.0)	3551 (99.8)	0.948
	Yes	8 (0.2)	0 (0.0)	8 (0.2)	
BloodElectrolyte-1-IsNormal (%)	Decrease	9 (0.2)	0 (0.0)	9 (0.3)	0.879
	Yes	3811 (99.8)	261 (100.0)	3550 (99.7)	
BloodElectrolyte-1-ClinicalSignificant (%)	No	3818 (99.9)	261 (100.0)	3557 (99.9)	1
	Yes	2 (0.1)	0 (0.0)	2 (0.1)	
BloodElectrolyte-2-IsNormal (%)	Decrease	6 (0.2)	0 (0.0)	6 (0.2)	0.643
	Increase	6 (0.2)	0 (0.0)	6 (0.2)	
	Yes	3808	261 (100.0)	3547 (99.7)	

		(99.7)			
BloodElectrolyte-2-ClinicalSignificant (%)	No	3817 (99.9)	261 (100.0)	3556 (99.9)	1
	Yes	3 (0.1)	0 (0.0)	3 (0.1)	
BloodElectrolyte-3-IsNormal (%)	Decrease	15 (0.4)	1 (0.4)	14 (0.4)	0.929
	Increase	2 (0.1)	0 (0.0)	2 (0.1)	
	Yes	3803 (99.6)	260 (99.6)	3543 (99.6)	
BloodElectrolyte-3-ClinicalSignificant (%)	No	3816 (99.9)	261 (100.0)	3555 (99.9)	1
	Yes	4 (0.1)	0 (0.0)	4 (0.1)	
25-OH-D3-1-IsNormal (%)	Decrease	91 (2.4)	8 (3.1)	83 (2.3)	0.735
	Increase	1 (0.0)	0 (0.0)	1 (0.0)	
	Yes	3711 (97.6)	253 (96.9)	3458 (97.6)	
25-OH-D3-1-ClinicalSignificant (%)	No	3802 (100.0)	261 (100.0)	3541 (100.0)	1
	Yes	1 (0.0)	0 (0.0)	1 (0.0)	
ESR-1-IsNormal (%)	Increase	318 (8.7)	67 (26.5)	251 (7.4)	<0.001
	Yes	3327 (91.3)	186 (73.5)	3141 (92.6)	
ESR-1-ClinicalSignificant (%)	No	3629 (99.6)	252 (99.6)	3377 (99.6)	1
	Yes	16 (0.4)	1 (0.4)	15 (0.4)	
Rheumatism-1-IsNormal (%)	Increase	15 (0.4)	2 (0.8)	13 (0.4)	0.64
	Yes	3630 (99.6)	251 (99.2)	3379 (99.6)	
Rheumatism-2-IsNormal (%)	Increase	76 (2.1)	28 (11.1)	48 (1.4)	<0.001
	Yes	3569 (97.9)	225 (88.9)	3344 (98.6)	
Rheumatism-2-ClinicalSignificant (%)	No	3634 (99.7)	251 (99.2)	3383 (99.7)	0.382
	Yes	11 (0.3)	2 (0.8)	9 (0.3)	
Rheumatism-3-IsNormal (%)	Increase	5 (0.1)	1 (0.4)	4 (0.1)	0.788
	Yes	3640 (99.9)	252 (99.6)	3388 (99.9)	
Rheumatism-3-ClinicalSignificant (%)	No	3644 (100.0)	253 (100.0)	3391 (100.0)	1
	Yes	1 (0.0)	0 (0.0)	1 (0.0)	
HLA-B27-1 (%)	Negative	3642 (99.9)	252 (99.6)	3390 (99.9)	0.507
	Positive	3 (0.1)	1 (0.4)	2 (0.1)	
HLA-B27-1-IsNormal (%)	No	2 (0.1)	0 (0.0)	2 (0.1)	1
	Yes	3643 (99.9)	253 (100.0)	3390 (99.9)	
HLA-B27-1-ClinicalSignificant (%)	No	3644 (100.0)	253 (100.0)	3391 (100.0)	1
	Yes	1 (0.0)	0 (0.0)	1 (0.0)	
the stage of your illness (nearly 3 months) (%)	progressive	2994 (79.4)	202 (78.0)	2792 (79.5)	0.627
	regressive	253 (6.7)	16 (6.2)	237 (6.7)	
	stationary	524 (13.9)	41 (15.8)	483 (13.8)	
onset seasons (%)	Autumn	459 (11.6)	31 (11.7)	428 (11.6)	0.204
	CannotRemember	1675 (42.3)	125 (47.2)	1550 (41.9)	
	Spring	494 (12.5)	32 (12.1)	462 (12.5)	
	Summer	535 (13.5)	38 (14.3)	497 (13.4)	
	Winter	798 (20.1)	39 (14.7)	759 (20.5)	

Supplementary Table 5. **Geographical distributions of patients with psoriasis included in our dataset.**

Province/Municipality	Number of patients
Inner Mongolia	1
Beijing	1
Ningxia	1
Xinjiang	2
Hainan	2
Gansu	2
Tianjin	2
Heilongjiang	2
Chongqing	2
Qinghai	3
Fujian	3
Liaoning	3
Sichuan	4
Shanxi	4
Shandong	4
Hebei	6
Shaanxi	7
Yunnan	7
Zhejiang	8
Shanghai	11
Jiangsu	13
Anhui	14
Guangxi	15
Henan	18
Hubei	32
Guizhou	39
Guangdong	46
Jiangxi	267
Hunan	3241
Unknown	201

Supplementary Table 6. Age distributions (%) of patients with PsA and non-PsA psoriasis.

Age group	PsA	Non-PsA psoriasis
<20	0.75%	9.75%
20-29	9.81%	18.68%
30-39	19.25%	21.42%
40-49	26.04%	19.84%
50-59	31.32%	19.16%
60-69	9.81%	8.14%
>=70	3.02%	3.02%

Supplementary Table 7. AUC values and their corresponding 95% CIs of univariate continuous numerical factors.

Feature	Lower AUC	AUC	Upper AUC
Age	0.5866	0.6188	0.6510
BMI	0.5238	0.5604	0.5971
BSA-1-A	0.4862	0.5190	0.5518
BSA-10-A	0.5158	0.5473	0.5787
BSA-11-A	0.5651	0.5967	0.6282
BSA-12-A	0.5462	0.5789	0.6115
BSA-13-A	0.4762	0.4950	0.5138
BSA-2-A	0.5079	0.5307	0.5535
BSA-3-A	0.5163	0.5348	0.5533
BSA-4-A	0.5750	0.6062	0.6374
BSA-5-A	0.5657	0.5975	0.6293
BSA-6-A	0.4612	0.4859	0.5107
BSA-7-A	0.5441	0.5758	0.6074
BSA-8-A	0.5286	0.5609	0.5932
BSA-9-A	0.4922	0.5002	0.5081
BSA	0.5788	0.6133	0.6477
DBP	0.5533	0.5861	0.6188
DLQI	0.5040	0.5374	0.5708
H	0.4770	0.5105	0.5439
Height	0.5518	0.5848	0.6179
PASI-1-A	0.5269	0.5615	0.5962
PASI-2-A	0.5852	0.6195	0.6538
PASI-3-A	0.6002	0.6340	0.6678
PASI-4-A	0.5853	0.6192	0.6532
PASI-4-D	0.4996	0.5335	0.5674
PASI-4-E	0.5046	0.5384	0.5723
PASI-4-I	0.5213	0.5547	0.5882
PASI	0.5786	0.6119	0.6451
SBP	0.5459	0.5787	0.6115
W	0.5364	0.5696	0.6029
Weight	0.4800	0.5135	0.5470
WHR	0.5189	0.5540	0.5891

Supplementary Table 8. Results of Kruskal-Wallis H test for testing if samples came from the same distribution, grouped by features.

Feature	Kruskal-Wallis H test *p*-value
Sex	0.000654
BloodRT-1-IsNormal	0.088992
BloodRT-1-CS	2.34E-05
BloodRT-2-IsNormal	5.02E-05
BloodRT-2-CS	0.792095
BloodRT-3-IsNormal	2.54E-05
BloodRT-3-CS	0.792095
BloodRT-4-IsNormal	0.886686
BloodRT-4-CS	0.000148
BloodRT-5-IsNormal	0.059098
BloodRT-5-CS	2.34E-05
BloodRT-6-IsNormal	0.000881
BloodRT-6-CS	0.000148
UrineRT-1	0.094291
UrineRT-1-IsNormal	0.205249
UrineRT-1-CS	0.55544
UrineRT-2	0.921757
UrineRT-2-IsNormal	0.574053
UrineRT-2-CS	0.70929
UrineRT-3	0.70354
UrineRT-3-IsNormal	0.432707
UrineRT-4	0.12791
UrineRT-4-IsNormal	0.75986
LiverRenal-1-IsNormal	0.000456
LiverRenal-1-CS	0.00236
LiverRenal-2-IsNormal	0.778826
LiverRenal-2-CS	0.792095
LiverRenal-3-IsNormal	0.965773
LiverRenal-3-CS	0.70929
LiverRenal-4-IsNormal	0.121028
LiverRenal-4-CS	0.48531
LiverRenal-5-IsNormal	0.055062
LiverRenal-5-CS	0.55544
LiverRenal-6-IsNormal	0.864562
LiverRenal-7-IsNormal	0.205249
LiverRenal-8-IsNormal	0.893268
LiverRenal-9-IsNormal	0.018191
LiverRenal-9-CS	0.000148
LiverRenal-10-IsNormal	0.053268
LiverRenal-10-CS	0.012545
BloodLipid-1-IsNormal	0.725645
BloodLipid-1-CS	0.55544
BloodLipid-2-IsNormal	0.000667
BloodLipid-2-CS	0.220369
BloodLipid-3-IsNormal	0.317247
BloodLipid-3-CS	0.55544
BloodLipid-4-IsNormal	0.613583
BloodLipid-4-CS	0.518302

BloodElectrolyte-1-IsNormal	0.518302
BloodElectrolyte-2-IsNormal	0.840439
BloodElectrolyte-3-IsNormal	0.743281
BloodElectrolyte-3-CS	0.792095
25-OH-D3-1-IsNormal	0.965359
25-OH-D3-1-CS	0.792095
ESR-1-IsNormal	2.93E-22
ESR-1-CS	0.647931
Rheumatism-1-IsNormal	0.079528
Rheumatism-2-IsNormal	3.63E-30
Rheumatism-2-CS	2.19E-09
Rheumatism-3-IsNormal	0.220369
HLA-B27--1	0.012545
HLA-B27-1-IsNormal	0.012545
HLA-B27-1-CS	0.000148
StageofIllness	1.83E-06
Season	3.26E-05

Supplementary Table 9. Results of the Kruskal-Wallis H test for testing if samples came from the same distribution, grouped by PsA labels.

Feature	Kruskal-Wallis H test *p*-value
Sex	0.000654
Age	1.48E-13
PASI	0.028298
BSA	0.000196
Height	5.29E-07
Weight	0.13758
W	0.034752
H	0.479134
WHR	0.006652
BMI	0.447088
SBP	1.70E-07
DBP	8.13E-08
PASI-1-A	0.393705
PASI-2-A	0.341248
PASI-3-A	0.053061
PASI-4-A	0.829827
PASI-4-E	0.27422
PASI-4-D	0.208167
PASI-4-I	0.027027
BSA-1	0.438875
BSA-2	0.076106
BSA-3	0.011661
BSA-4	1.16E-06
BSA-5	8.88E-06
BSA-6	0.162713
BSA-7	0.000526
BSA-8	0.015576
BSA-9	0.171404
BSA-10	0.849834
BSA-11	1.70E-05
BSA-12	0.001594
BSA-13	0.649367
BloodRT-1-IsNormal	0.076572
BloodRT-1-CS	2.34E-05
BloodRT-2-IsNormal	4.89E-05
BloodRT-2-CS	0.792095
BloodRT-3-IsNormal	2.04E-05
BloodRT-3-CS	0.792095
BloodRT-4-IsNormal	0.925184
BloodRT-4-CS	0.000148
BloodRT-5-IsNormal	0.191982
BloodRT-5-CS	2.34E-05
BloodRT-6-IsNormal	0.000344
BloodRT-6-CS	0.000148
UrineRT-1	0.094291
UrineRT-1-IsNormal	0.205249
UrineRT-1-CS	0.55544
UrineRT-2	0.921757

UrineRT-2-IsNormal	0.574053
UrineRT-2-CS	0.70929
UrineRT-3	0.70354
UrineRT-3-IsNormal	0.432707
UrineRT-4	0.12791
UrineRT-4-IsNormal	0.75986
LiverRenal-1-IsNormal	0.000496
LiverRenal-1-CS	0.00236
LiverRenal-2-IsNormal	0.778826
LiverRenal-2-CS	0.792095
LiverRenal-3-IsNormal	0.97587
LiverRenal-3-CS	0.70929
LiverRenal-4-IsNormal	0.300898
LiverRenal-4-CS	0.48531
LiverRenal-5-IsNormal	0.07973
LiverRenal-5-CS	0.55544
LiverRenal-6-IsNormal	0.864562
LiverRenal-7-IsNormal	0.205249
LiverRenal-8-IsNormal	0.968459
LiverRenal-9-IsNormal	0.019228
LiverRenal-9-CS	0.000148
LiverRenal-10-IsNormal	0.112424
LiverRenal-10-CS	0.012545
BloodLipid-1-IsNormal	0.752433
BloodLipid-1-CS	0.55544
BloodLipid-2-IsNormal	0.513057
BloodLipid-2-CS	0.220369
BloodLipid-3-IsNormal	0.356035
BloodLipid-3-CS	0.55544
BloodLipid-4-IsNormal	0.681287
BloodLipid-4-CS	0.518302
BloodElectrolyte-1-IsNormal	0.518302
BloodElectrolyte-2-IsNormal	0.55544
BloodElectrolyte-3-IsNormal	0.860336
BloodElectrolyte-3-CS	0.792095
25-OH-D3-1-IsNormal	0.944743
25-OH-D3-1-CS	0.792095
ESR-1-IsNormal	2.93E-22
ESR-1-CS	0.647931
Rheumatism-1-IsNormal	0.079528
Rheumatism-2-IsNormal	3.63E-30
Rheumatism-2-CS	2.19E-09
Rheumatism-3-IsNormal	0.220369
HLA-B27--1	0.012545
HLA-B27-1-IsNormal	0.012545
HLA-B27-1-CS	0.000148
DLQI	0.293608
StageofIllness	0.000783
Season	0.000106

Supplementary Table 10. The univariate HRs of cox regression models.

Feature	HR	Low HR	High HR	Wald test *p*-value
Age	1.03	1.013	1.048	0.0004075
PASI	1.016	0.9904	1.043	0.222
BSA	1.006	0.9937	1.019	0.3258
Height	0.9936	0.9696	1.018	0.6075
Weight	0.9928	0.9761	1.01	0.405
W	1.013	1.001	1.025	0.03602
H	1.005	0.9804	1.03	0.6958
WHR	0.9842	0.972	0.9967	0.01315
BMI	1.019	0.9698	1.072	0.4505
SBP	1.015	0.9988	1.032	0.06879
DBP	1.022	0.9942	1.051	0.1218
PASI-1-A	0.9961	0.9764	1.016	0.7036
PASI-2-A	1.003	0.9921	1.014	0.6121
PASI-3-A	1.004	0.9892	1.018	0.6225
PASI-4-A	1.006	0.9957	1.017	0.2496
PASI-4-E	1.186	0.8777	1.602	0.2669
PASI-4-D	1.074	0.8228	1.402	0.5994
PASI-4-I	1.173	0.8874	1.551	0.2624
BSA-1-A	1.093	0.8309	1.438	0.5247
BSA-2-A	0.7586	0.3768	1.527	0.4389
BSA-3-A	1.013	0.4844	2.118	0.9726
BSA-4-A	1.11	0.956	1.29	0.1705
BSA-5-A	1.105	0.9355	1.306	0.2395
BSA-6-A	1.073	0.8048	1.43	0.6323
BSA-7-A	1.017	0.9339	1.108	0.6926
BSA-8-A	1.031	0.966	1.1	0.3576
BSA-9-A	0.5679	0.07022	4.593	0.5958
BSA-10-A	1.192	1.017	1.396	0.02987
BSA-11-A	1.03	0.9878	1.073	0.1674
BSA-12-A	1.018	0.9542	1.085	0.5946
BSA-13-A	0.9652	0.716	1.301	0.8162
DLQI	1.014	0.9686	1.062	0.5485
Sex				0.1657
Male	1	1	1	
Female	1.406	0.8685	2.276	
bloodRT-1-IsNormal				0.06024
Yes	1	1	1	
Increase	2.241	0.9658	5.2	
bloodRT-1-ClinicalSignificant				0.1725
No	1	1	1	
Yes	3.969	0.5478	28.77	
bloodRT-2-IsNormal				0.02711
Yes	1	1	1	
Increase	2.802	1.124	6.987	
bloodRT-3-IsNormal				0.001552436
Yes	1	1	1	
Decrease	3.6861	1.3585	10	
Increase	0.9319	0.4156	2.09	
bloodRT-4-ClinicalSignificant				0.1745
No	1	1	1	
Yes	3.943	0.5443	28.57	
bloodRT-5-IsNormal				0.5061
Yes	1	1	1	
Decrease	1.612	0.3947	6.58	
bloodRT-6-IsNormal				0.000007477
Yes	1	1	1	

Increase	5.417	2.587	11.34	
UrineRT-1				0.6877
Negative	1	1	1	
Positive	1.5	0.2079	10.82	
UrineRT-2				0.0007957
Negative	1	1	1	
Positive	11.27	2.737	46.43	
UrineRT-2-IsNormal				0.02849
Yes	1	1	1	
No	9.144	1.262	66.23	
UrineRT-2-ClinicalSignificant				0.01729
No	1	1	1	
Yes	11.12	1.53	80.78	
UrineRT-3				0.1769
Negative	1	1	1	
Positive	1.781	0.7706	4.116	
UrineRT-3-IsNormal				0.2488
Yes	1	1	1	
No	1.977	0.6207	6.3	
UrineRT-4				0.08849
Negative	1	1	1	
Positive	2.739	0.8592	8.733	
UrineRT-4-IsNormal				0.5983
Yes	1	1	1	
No	1.701	0.2356	12.28	
LiverRenal-1-IsNormal				0.1632
Yes	1	1	1	
Decrease	3.505	0.8622	14.247	
Increase	1.301	0.3236	5.229	
LiverRenal-1-ClinicalSignificant				0.2001
No	1	1	1	
Yes	3.646	0.504	26.37	
LiverRenal-2-IsNormal				0.7102
Yes	1	1	1	
Increase	1.211	0.4406	3.33	
LiverRenal-3-IsNormal				0.853
Yes	1	1	1	
Increase	1.1	0.4007	3.02	
LiverRenal-7-IsNormal				0.8188
Yes	1	1	1	
Increase	1.261	0.1737	9.149	
LiverRenal-8-IsNormal				0.519
Yes	1	1	1	
Decrease	1.395	0.5073	3.835	
LiverRenal-10-IsNormal				0.5096
Yes	1	1	1	
Increase	1.282	0.6131	2.679	
BloodLipid-1-IsNormal				0.8568
Yes	1	1	1	
Increase	0.9402	0.4813	1.837	
BloodLipid-2-IsNormal				0.07242
Yes	1	1	1	
Increase	1.65	0.9554	2.851	
BloodLipid-2-ClinicalSignificant				0.3695
No	1	1	1	
Yes	2.477	0.3417	17.95	
BloodLipid-3-IsNormal				0.04906
Yes	1	1	1	
Decrease	0.9795	0.4304	2.229	

Increase	0.5225	0.2689	1.015	
BloodLipid-3-ClinicalSignificant				0.07789
No	1	1	1	
Yes	5.944	0.8195	43.11	
BloodLipid-4-IsNormal				0.1876
Yes	1	1	1	
Decrease	1.11	0.72406	1.701	
Increase	0.242	0.04776	1.227	
ESR-1-IsNormal				0.0008417
Yes	1	1	1	
Increase	2.734	1.515	4.933	
ESR-1-ClinicalSignificant				0.5749
No	1	1	1	
Yes	1.762	0.2434	12.75	
Rheumatism-2-IsNormal				1.80E-05
Yes	1	1	1	
Increase	6.343	2.727	14.75	
Rheumatism-2-ClinicalSignificant				2.92E-02
No	1	1	1	
Yes	9.054	1.25	65.58	
The stage of your illness				0.3081
Stationary	1	1	1	
Regressive	0.7326	0.4773	1.125	
Progressive	1.4723	0.5654	3.833	
Onset seasons				0.7465
Spring	1	1	1	
Summer	0.8284	0.472	1.454	
Autumn	1.0018	0.5485	1.83	
Winter	1.428	0.7771	2.624	
Cannot Remember	1.2474	0.6457	2.41	

Supplement Table 11. The C-statistics, corresponding 95%CIs, and changes in C-statistics for the Cox regression model adjustment.

Feature	C- statistic (95% CI)	Change in C-statistic (95% CI)
Lasso selected factors (BloodRT-2-IsNormal, BloodRT-3-IsNormal, BloodRT-6-IsNormal, UrineRT-2, UrineRT-4, LiverRenal-1-IsNormal, Rheumatism-2-IsNormal, illness stage, age, BSA-10-A, ESR-1-IsNormal)	0.6117 (0.4840-0.7325)	
BloodRT-3-IsNormal, BloodRT-6-IsNormal, UrineRT-2, UrineRT-4, LiverRenal-1-IsNormal, Rheumatism-2-IsNormal, illness stage, age, BSA-10-A, ESR-1-IsNormal	0.6212 (0.4922-0.7395)	- BloodRT-2-IsNormal<br>0.0100 (-0.0050-0.0622)
BloodRT-3-IsNormal, BloodRT-6-IsNormal, UrineRT-2, UrineRT-4, Rheumatism-2-IsNormal, illness stage, age, BSA-10-A, ESR-1-IsNormal	0.6360 (0.5057-0.7518)	- BloodRT-2-IsNormal<br>- LiverRenal-1-IsNormal<br>0.0253 (-0.0088-0.0876)
BloodRT-6-IsNormal, UrineRT-2, UrineRT-4, Rheumatism-2-IsNormal, illness stage, age, BSA-10-A, ESR-1-IsNormal	0.6424 (0.5218-0.7541)	- BloodRT-2-IsNormal<br>- LiverRenal-1-IsNormal<br>- BloodRT-3-IsNormal<br>0.0320 (-0.0220-0.1141)
BloodRT-6-IsNormal, UrineRT-2, Rheumatism-2-IsNormal, illness stage, age, BSA-10-A	0.6571 (0.5408-0.7650)	- BloodRT-2-IsNormal<br>- LiverRenal-1-IsNormal<br>- BloodRT-3-IsNormal<br>- ESR-1-IsNormal<br>0.0379 (-0.0209- 0.1255)
BloodRT-6-IsNormal, UrineRT-2, Rheumatism-2-IsNormal, illness stage, age, BSA-10-A, sex	0.6582 (0.5424-0.7671)	- BloodRT-2-IsNormal<br>- LiverRenal-1-IsNormal<br>- BloodRT-3-IsNormal<br>- ESR-1-IsNormal<br>+ sex<br>0.0371(-0.0369-0.1292)

Supplementary Figures

Supplementary Figure 1. Feature analysis for PsA diagnosis. (A) A visualization of the feature distributions of psoriasis patients. Samples are arranged in descending order of PASI. We demonstrated the feature distributions based on different percentages of samples according to individual PASI values. The BSA value has a similar distribution tendency to PASI. (B) The univariate ROC curve analysis of numeric variables. The blue dots indicate that the feature belongs to the category of physical examination (PE), while the purple dots are the features belonging to the category of the indices for severity and extent of psoriasis (ISEP). (C) The ROC curve and violin plot of the best-performing variable (i.e., PASI-3-A) in the univariate ROC curve analysis. (D) A correlation plot depicting the Spearman correlation coefficients between continuous numeric variables.

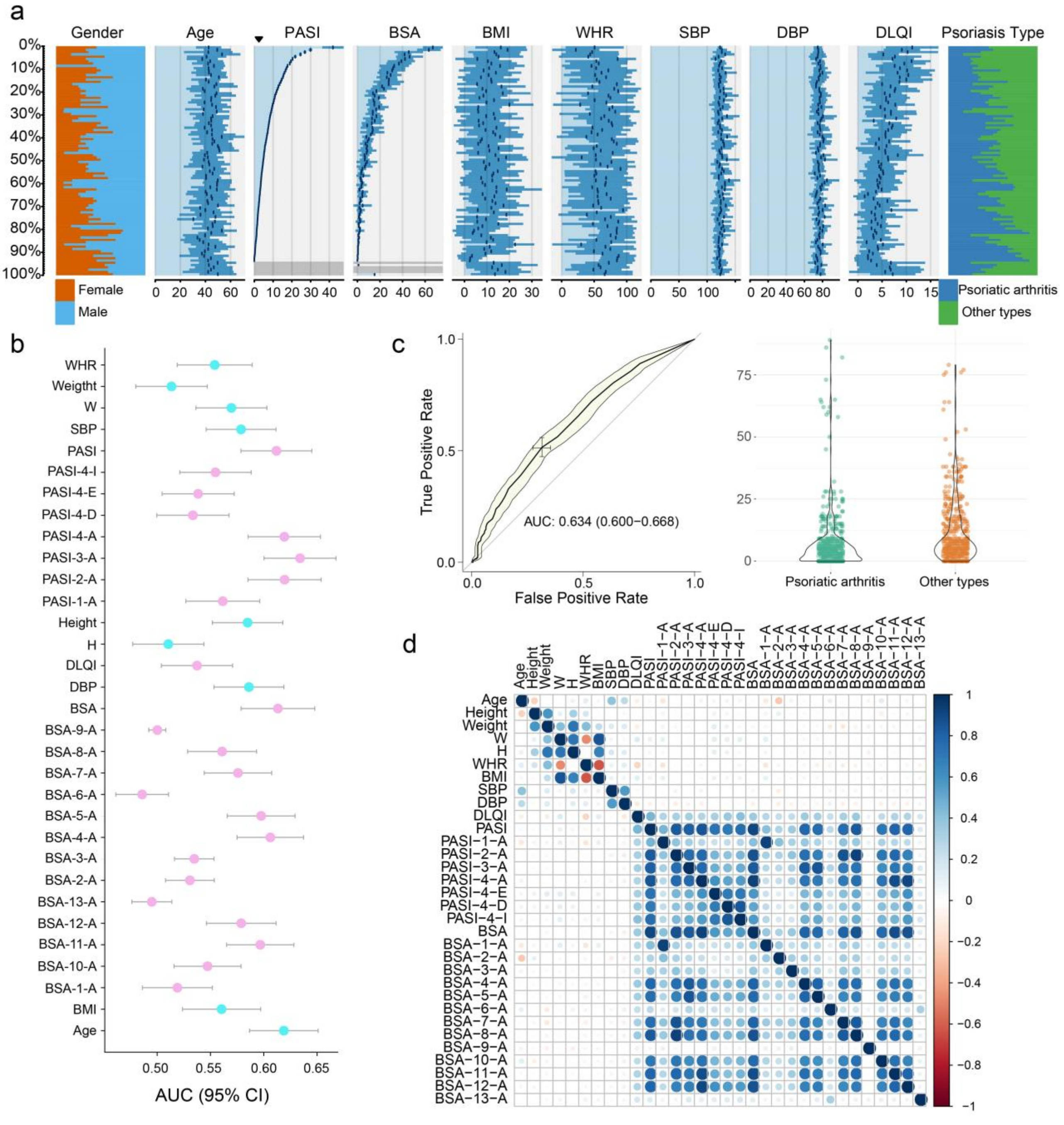

Supplementary Figure 2. Visualization of physical examination (PE) and PASI feature distributions of psoriasis patients. Samples are arranged in descending order of PASI. Features with large distribution ranges were preprocessed logarithmically. We demonstrated the feature distributions based on different percentages of samples according to individual PASI values. W=waistline. H=hipline.

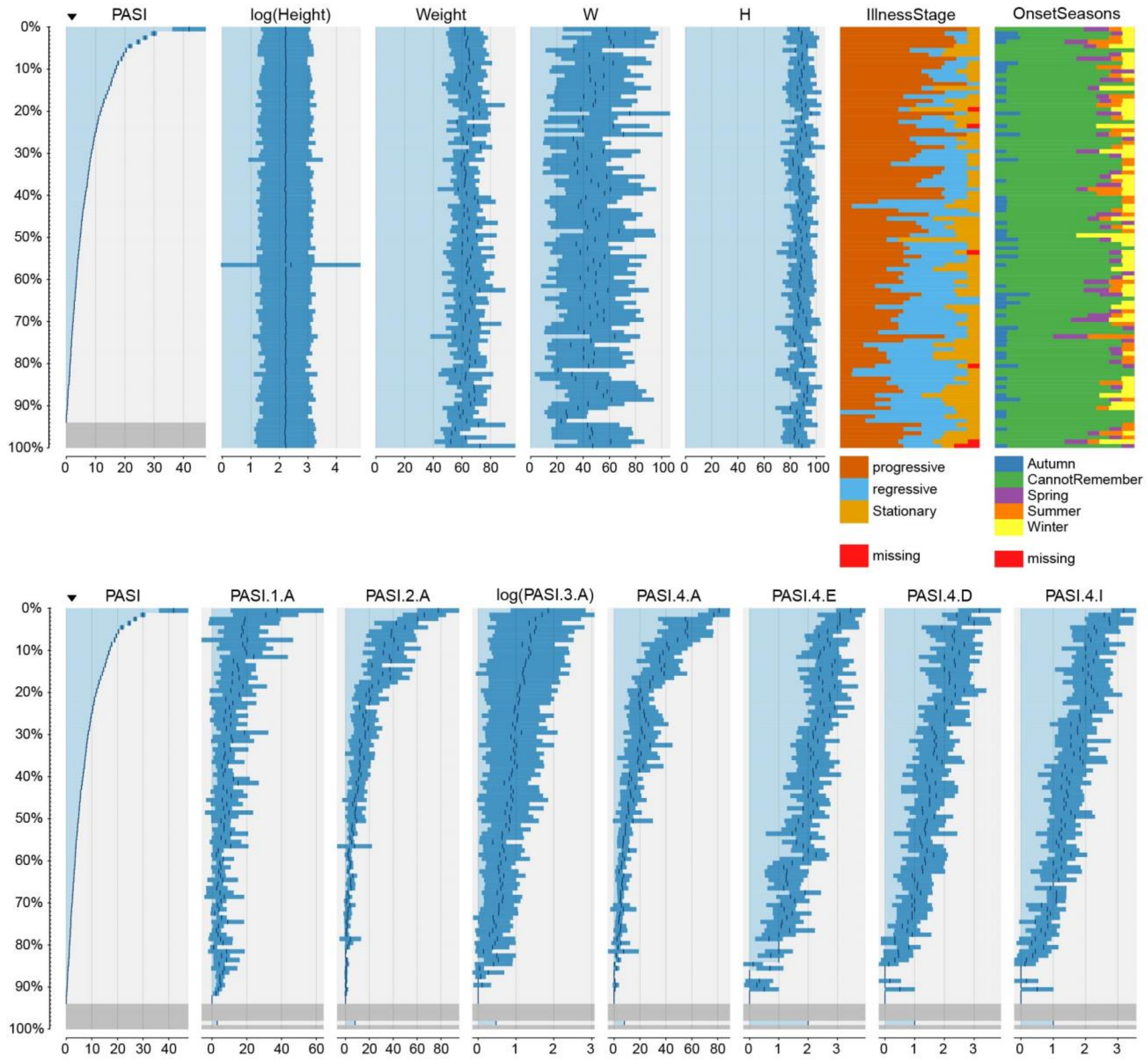

Supplementary Figure 3. Visualization of BSA feature distributions of psoriasis patients. Samples are arranged in descending order of PASI. Features with large distribution ranges were preprocessed logarithmically. We demonstrated the feature distributions based on different percentages of samples according to individual PASI values.

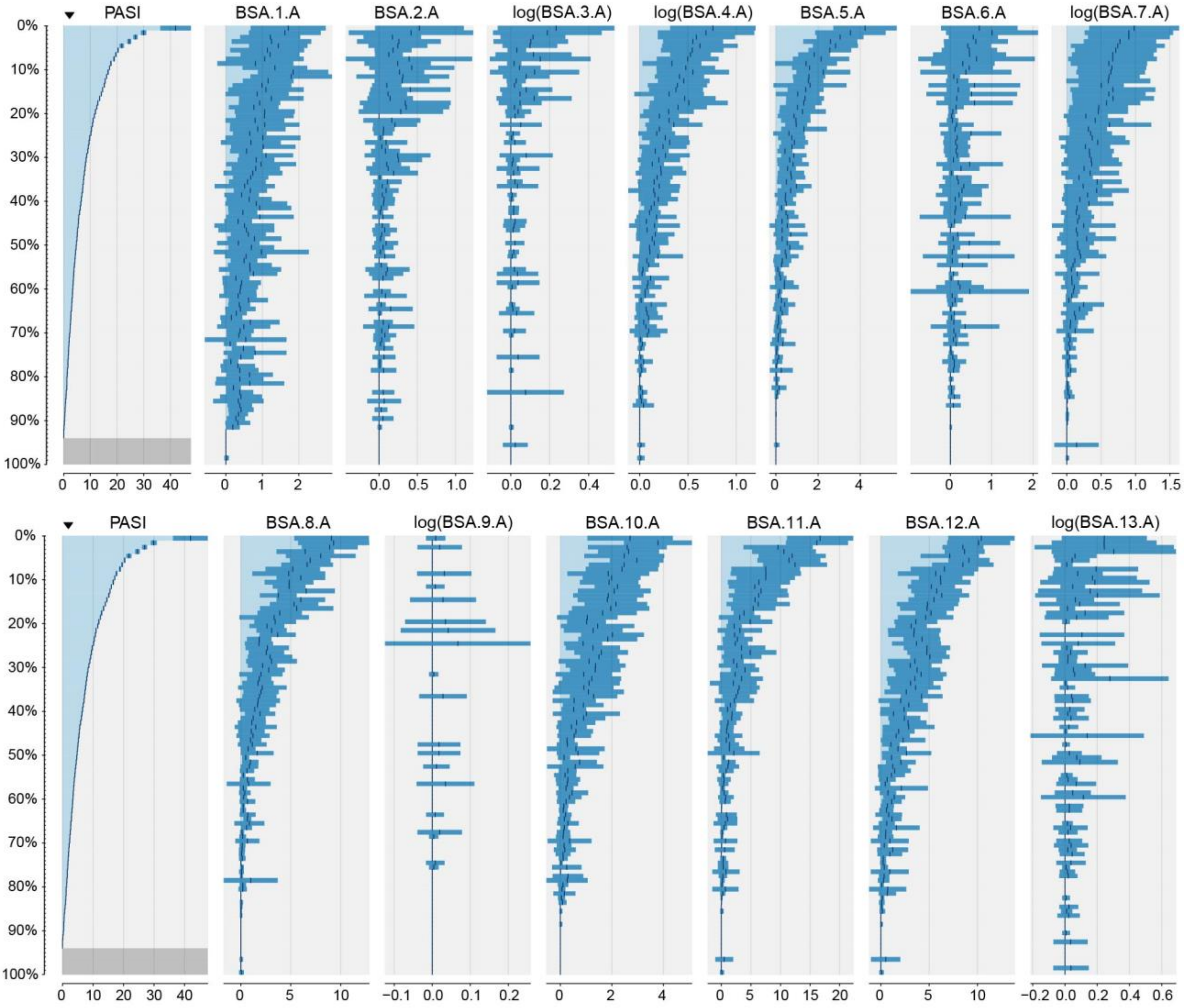

Supplementary Figure 4. Visualization of blood routine test feature distributions of psoriasis patients. Samples are arranged in descending order of PASI. Features with large distribution ranges were preprocessed logarithmically. We demonstrated the feature distributions based on different percentages of samples according to individual PASI values. BloodRT=blood routine test. CS=clinically significant.

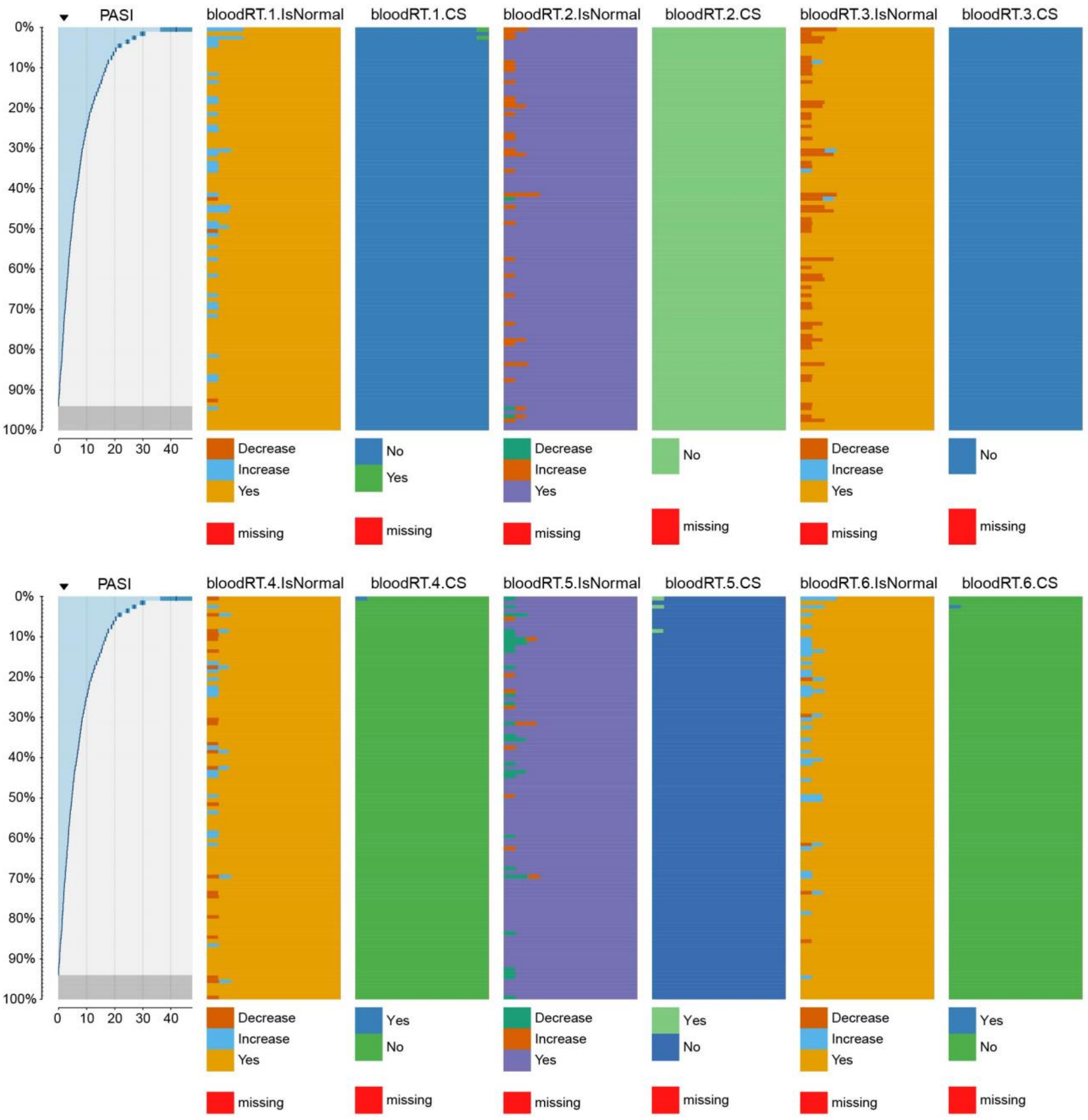

Supplementary Figure 5. Visualization of urine routine test feature distributions of psoriasis patients. Samples are arranged in descending order of PASI. Features with large distribution ranges were preprocessed logarithmically. We demonstrated the feature distributions based on different percentages of samples according to individual PASI values. UrineRT=urine routine test. CS=clinically significant.

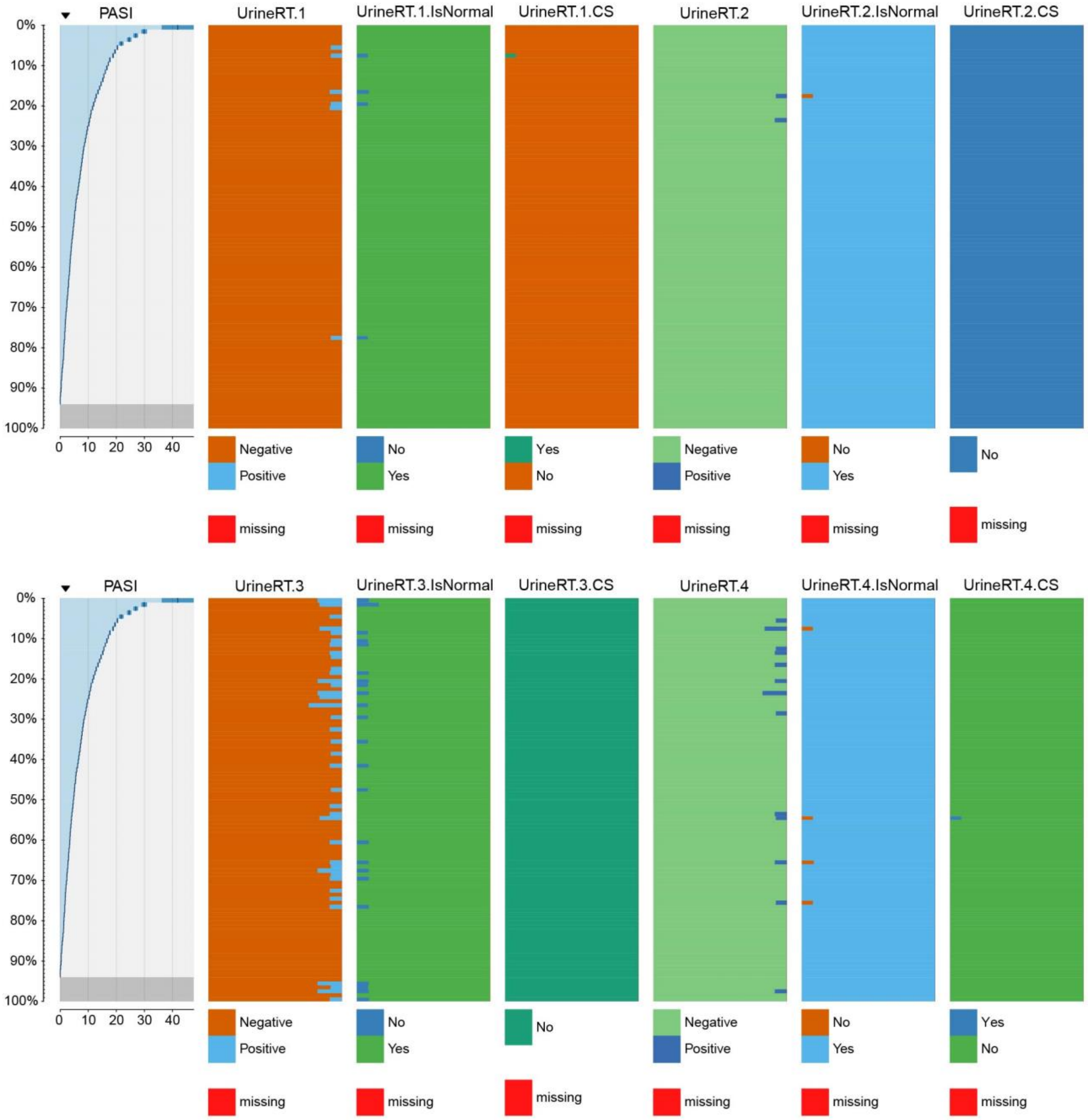

Supplementary Figure 6. Visualization of liver and kidney function (LKF) feature distributions of psoriasis patients. Samples are arranged in descending order of PASI. Features with large distribution ranges were preprocessed logarithmically. We demonstrated the feature distributions based on different percentages of samples according to individual PASI values. CS=clinically significant.

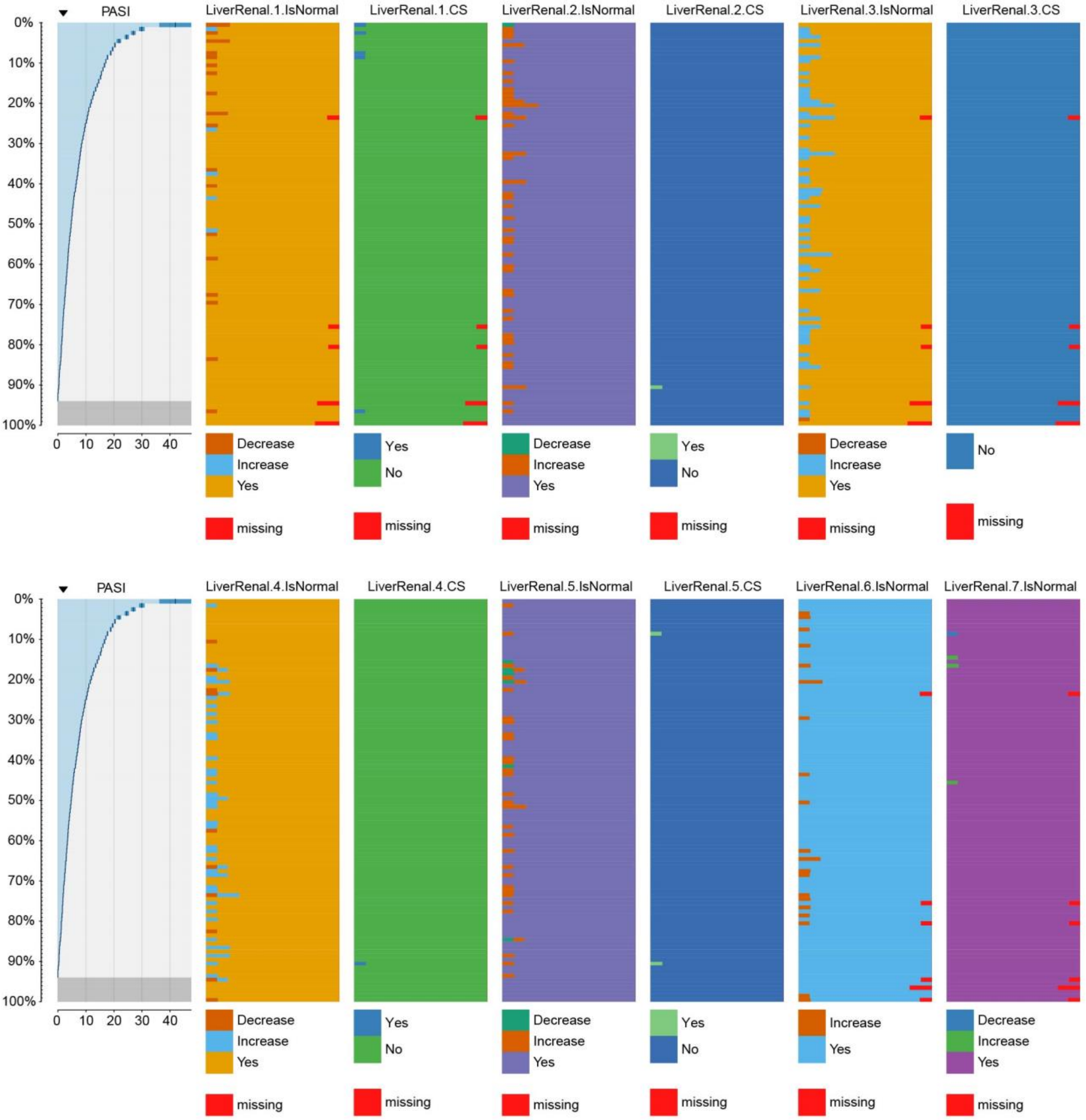

Supplementary Figure 7. Visualization of liver and kidney function (LKF) and blood lipid feature distributions of psoriasis patients. Samples are arranged in descending order of PASI. Features with large distribution ranges were preprocessed logarithmically. We demonstrated the feature distributions based on different percentages of samples according to individual PASI values. BloodRT=blood routine test. CS=clinically significant.

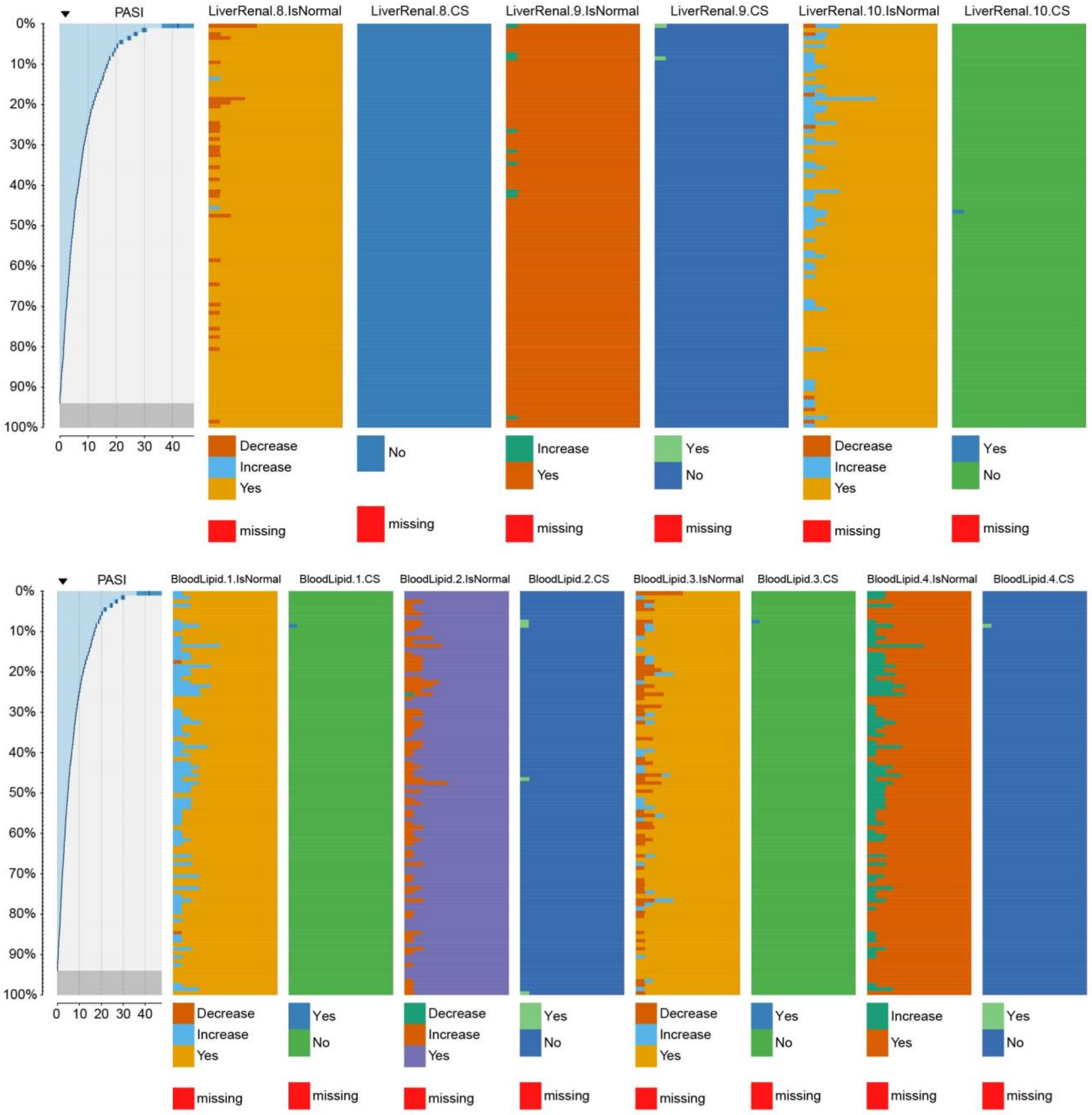

Supplementary Figure 8. Visualization of blood electrolyte, erythrocyte sedimentation rate and rheumatism factor feature distributions of psoriasis patients. Samples are arranged in descending order of PASI. Features with large distribution ranges were preprocessed logarithmically. We demonstrated the feature distributions based on different percentages of samples according to individual PASI values. ESR=erythrocyte sedimentation rate. CS=clinically significant.

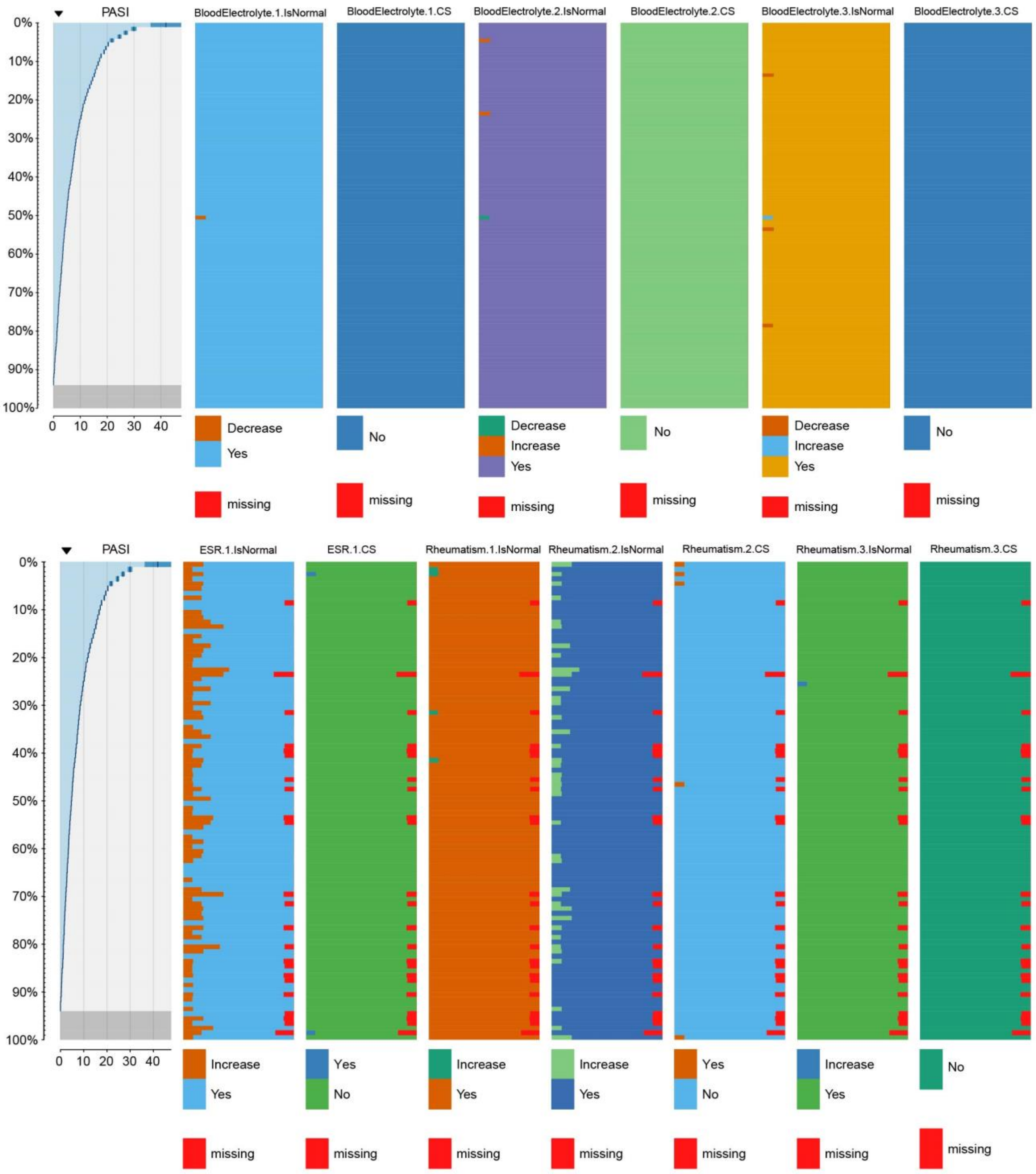

Supplementary Figure 9. The univariate ROC curves for PsA prediction. The AUC values and CIs are presented. W=waistline. H=hipline. WHR=waist-hip ratio. SBP=systolic blood pressure. DBP=diastolic blood pressure. DLQI=dermatology life quality index.

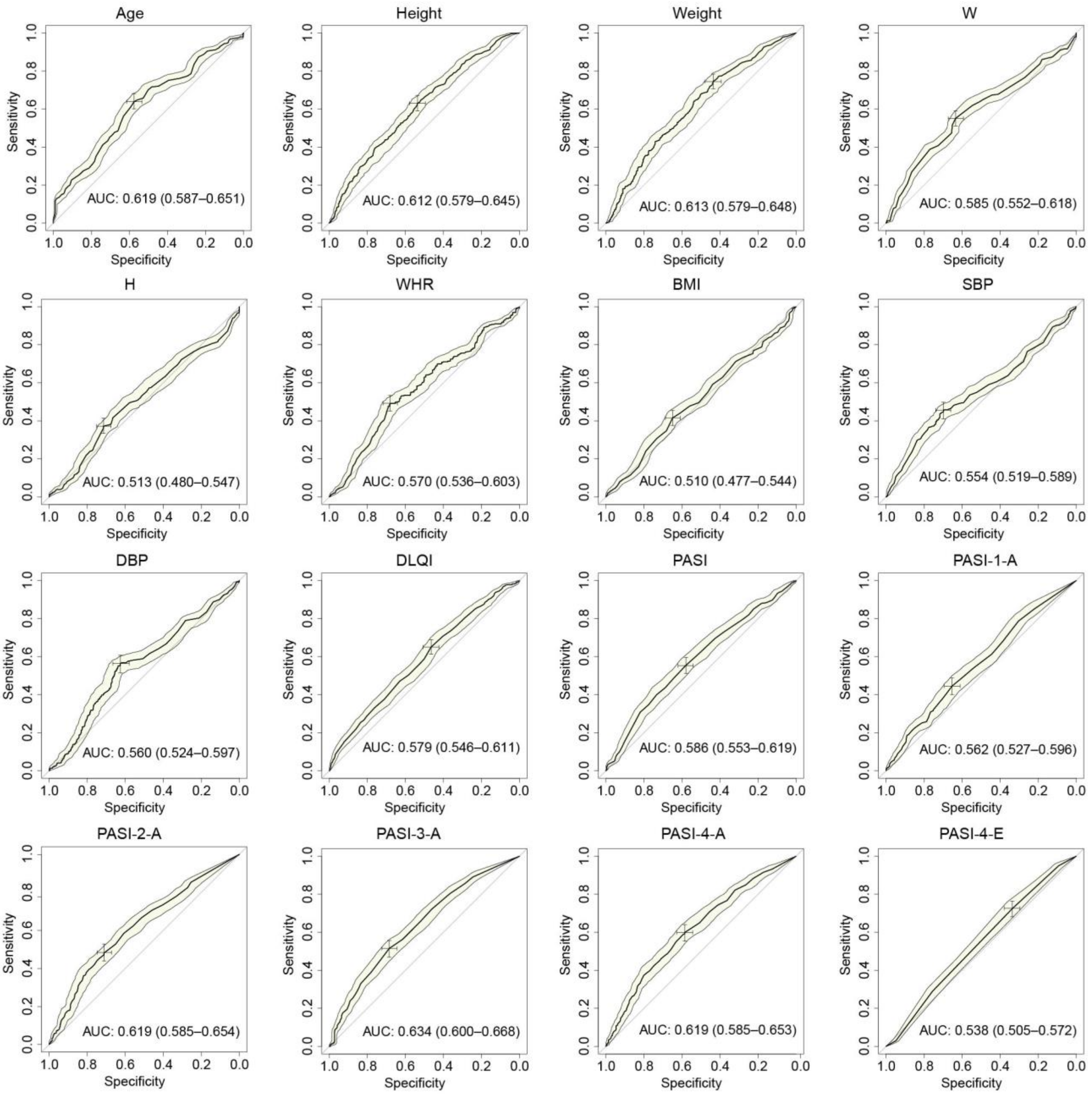

Supplementary Figure 10. The univariate ROC curves of PASI and BSA features for PsA prediction. The AUC values and CIs are presented.

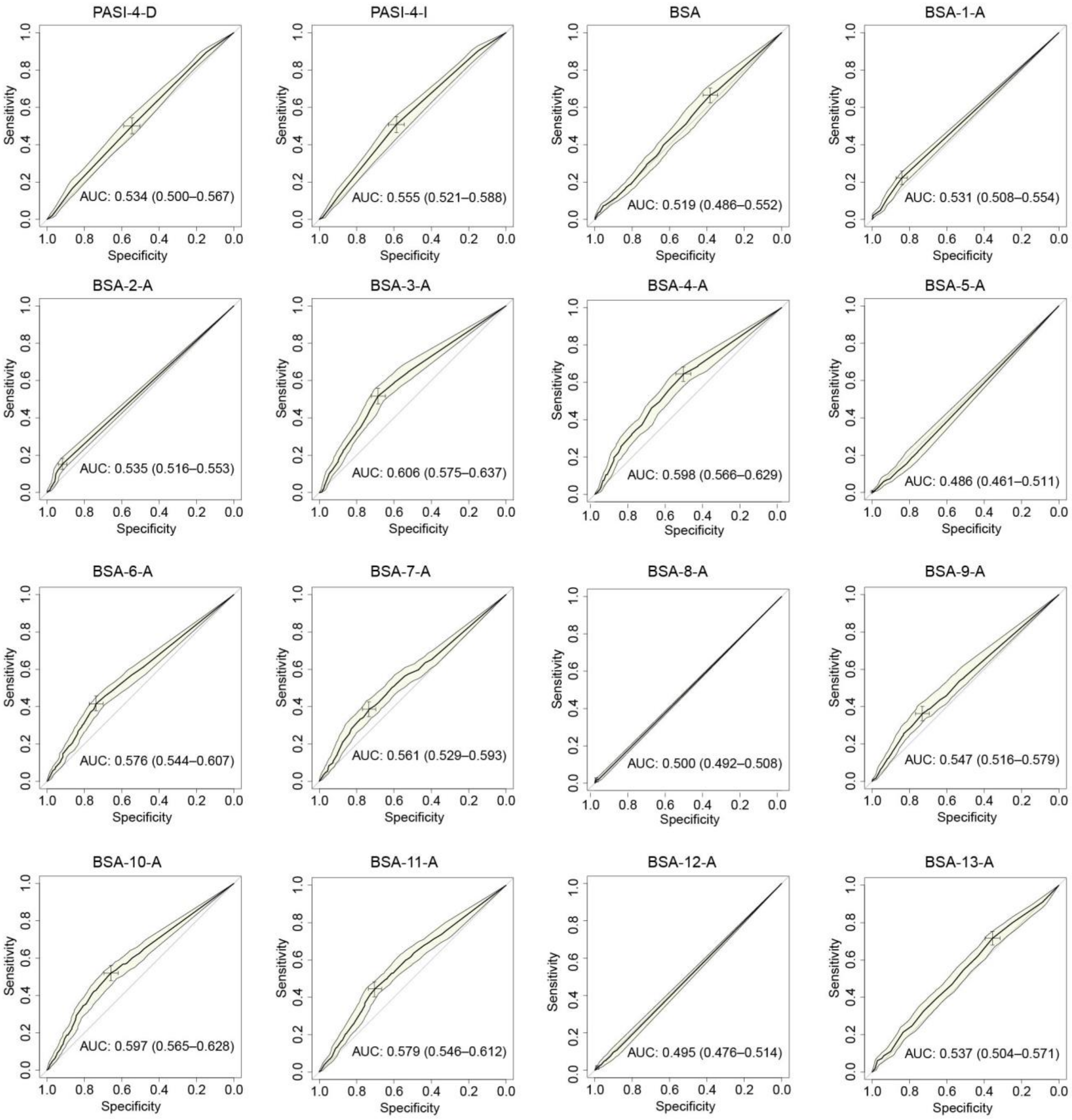

Supplementary Figure 11. Box plots of 10-fold validation test results of different machine learning methods trained on 40 random downsampling sub-training datasets utilizing Chi-Squared statistic feature selection strategy. The central line marks the median AUC value, and the bounds of the box mark the first and third quartiles.

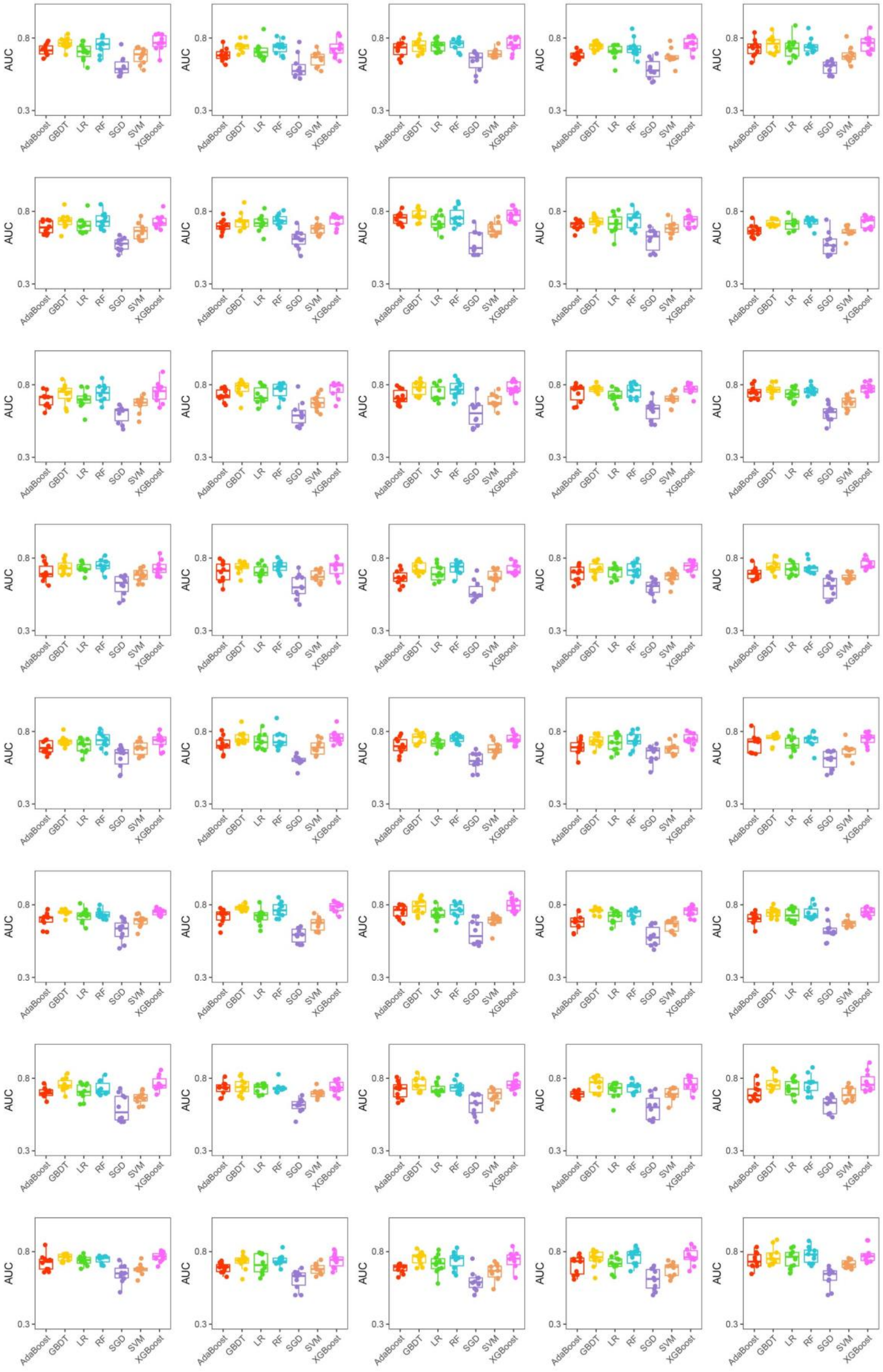

Supplementary Figure 12. Box plots of 10-fold validation test results of different machine learning methods trained on 40 random downsampling sub-training datasets utilizing the F-statistic (F) feature selection strategy. The central line marks the median AUC value, and the bounds of the box mark the first and third quartiles.

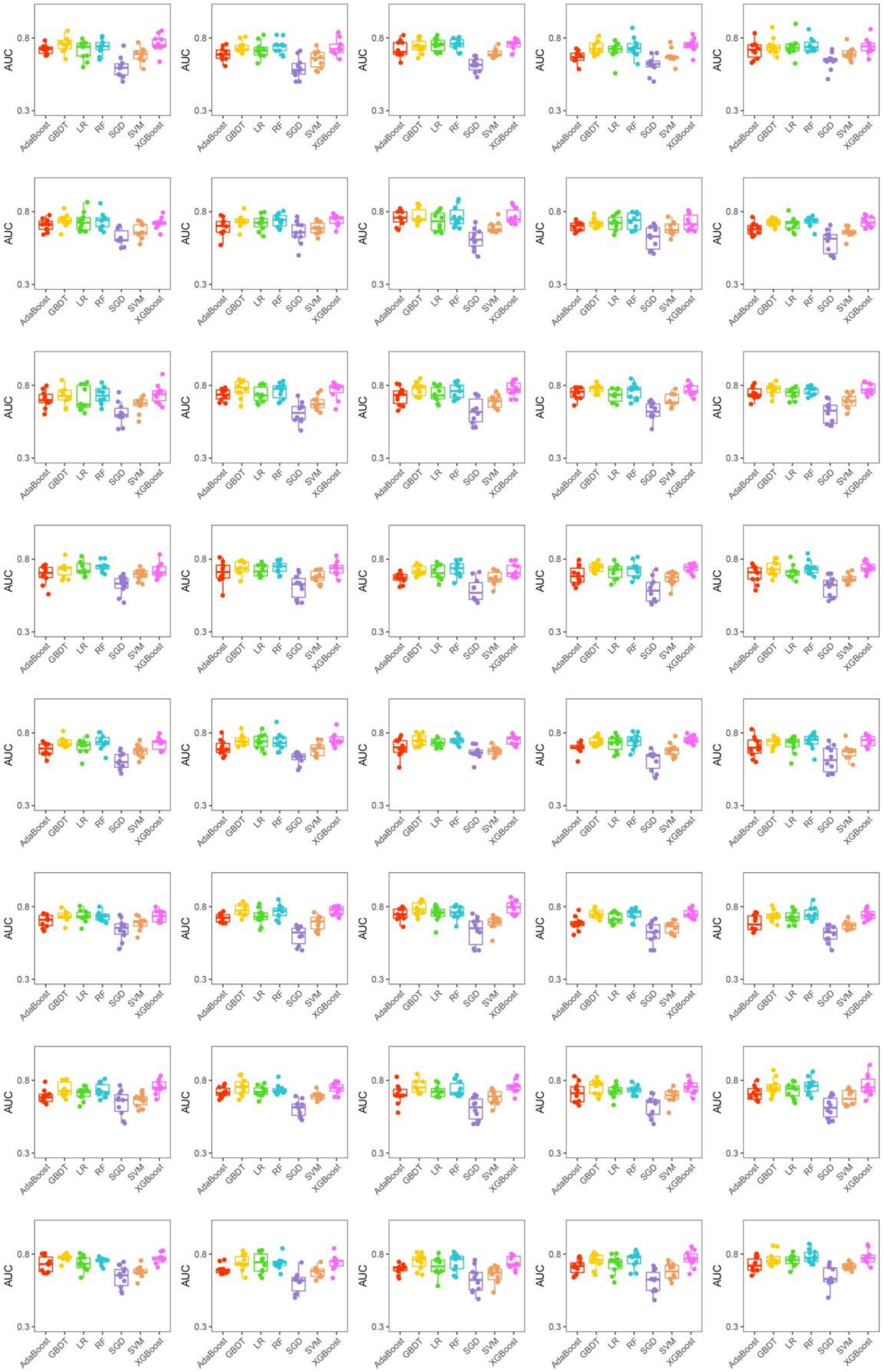

Supplementary Figure 13. Box plots of 10-fold validation test results of different machine learning methods trained on 40 random downsampling sub-training datasets utilizing mutual information (MI) feature selection strategy. The central line marks the median AUC value, and the bounds of the box mark the first and third quartiles.

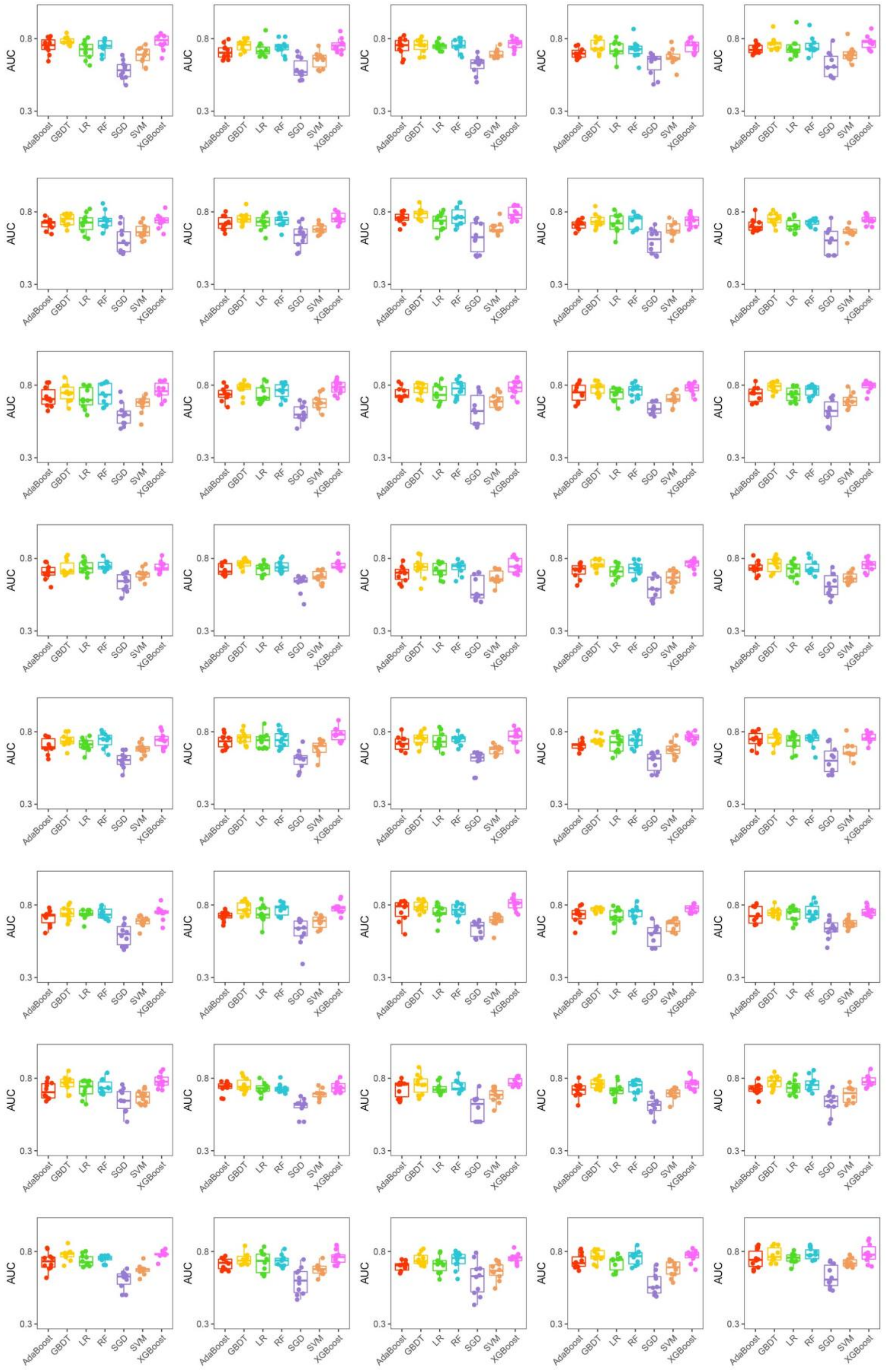

Supplementary Figure 14. Box plots of 10-fold validation test results of different machine learning methods trained on 40 random downsampling sub-training datasets utilizing max-relevance and min-redundancy (mRMR) feature selection strategy. The central line marks the median AUC value, and the bounds of the box mark the first and third quartiles.

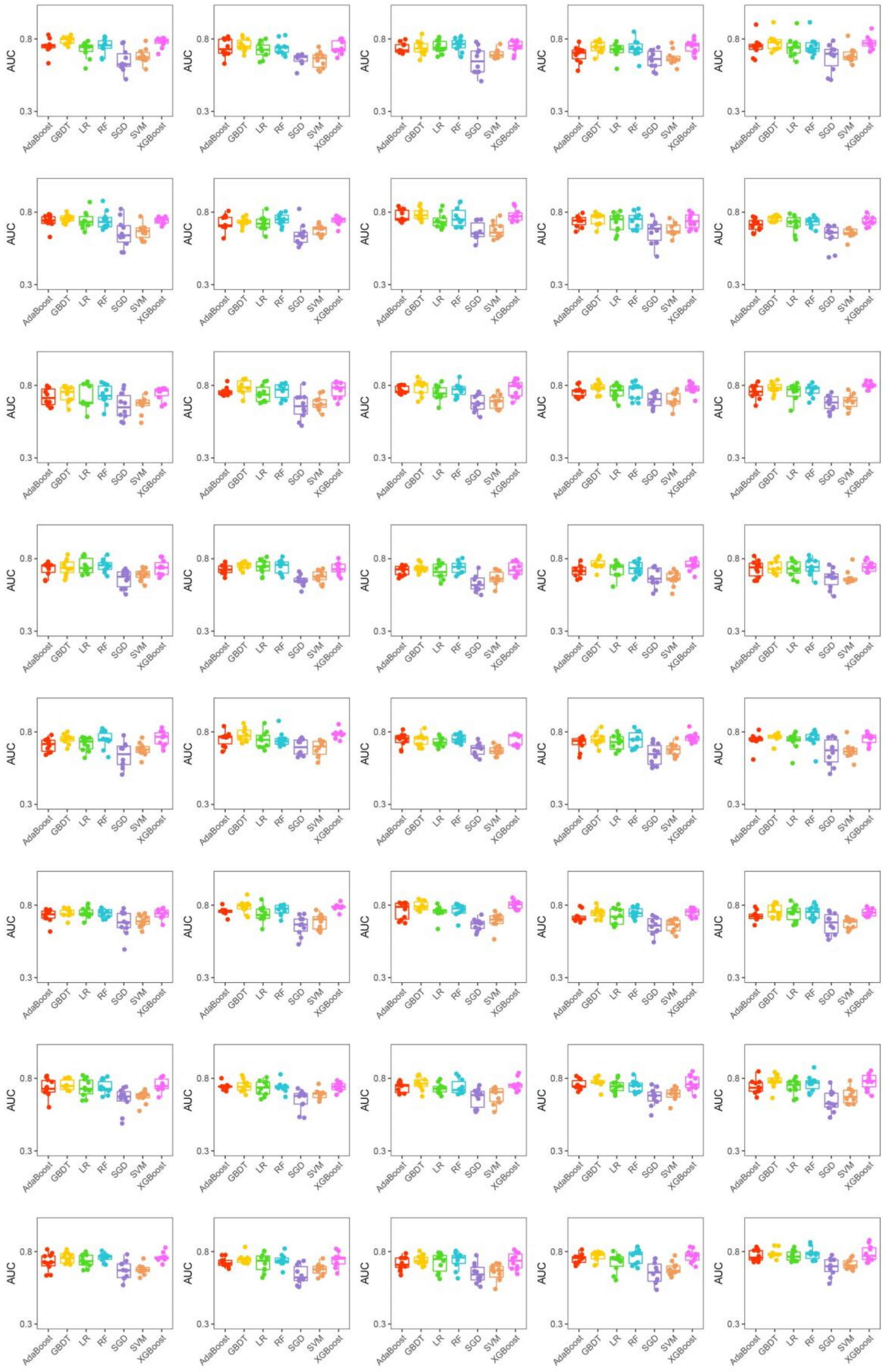

Supplementary Figure 15. Box plots of 10-fold validation test results of different machine learning methods trained on 40 random downsampling sub-training datasets utilizing recursive feature elimination (RFE) feature selection strategy. The central line marks the median AUC value, and the bounds of the box mark the first and third quartiles.

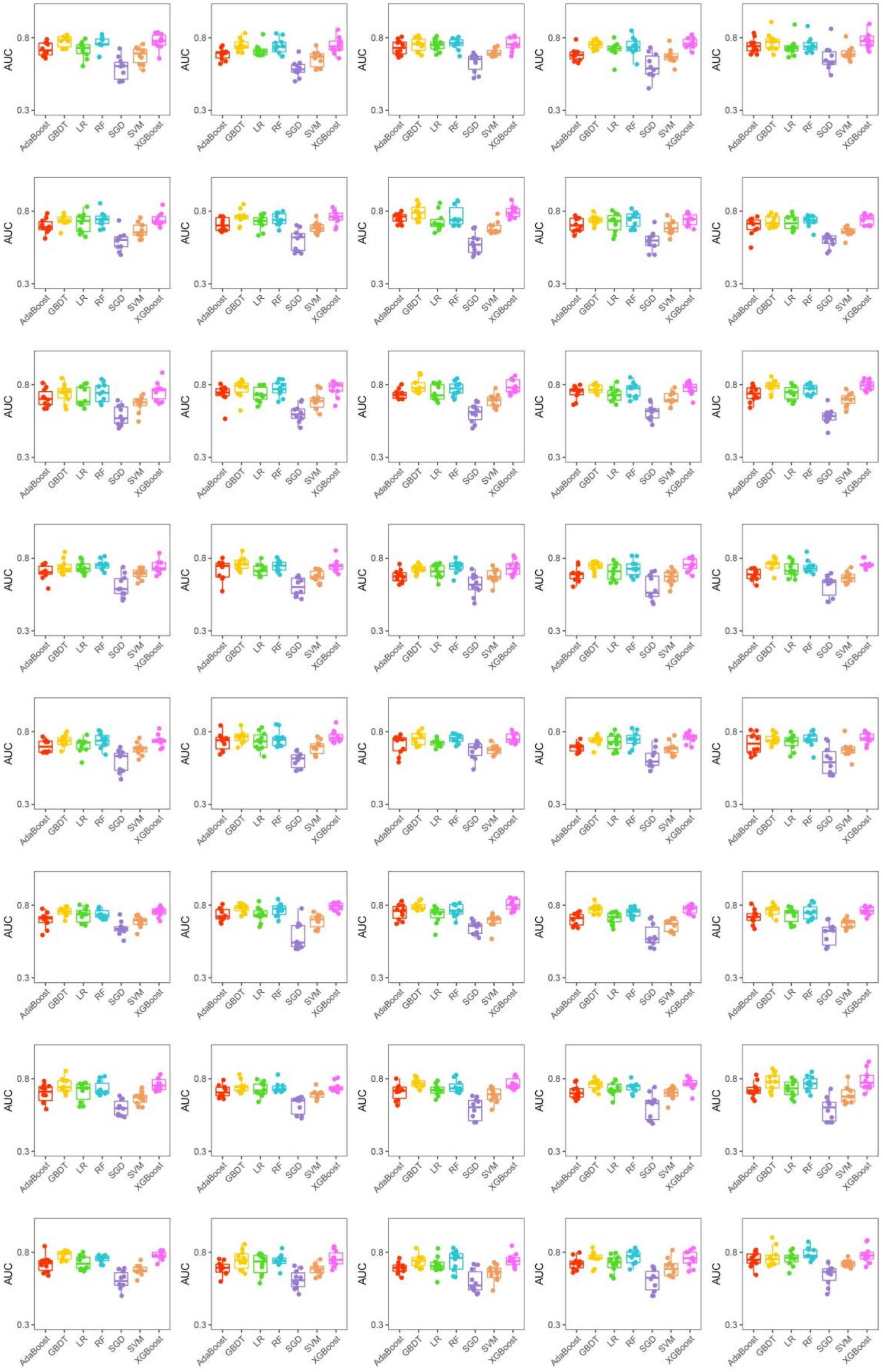

Supplementary Figure 16. ROC curves of test dataset of different machine learning methods trained on 40 random downsampling sub-training datasets utilizing Chi-Square feature selection strategy.

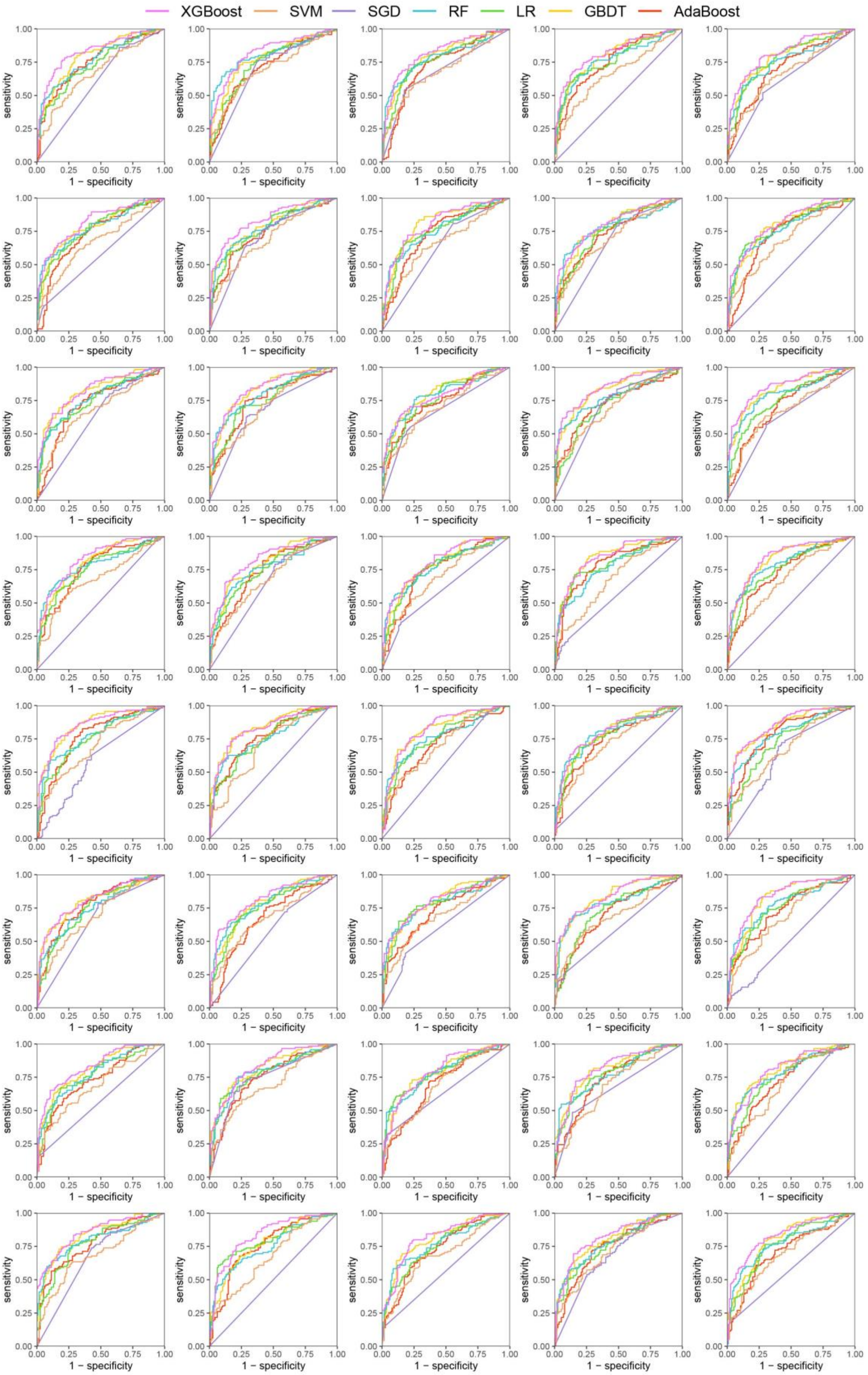

Supplementary Figure 17. ROC curves of test dataset of different machine learning methods trained on 40 random downsampling sub-training datasets utilizing the F-statistic (F) feature selection strategy.

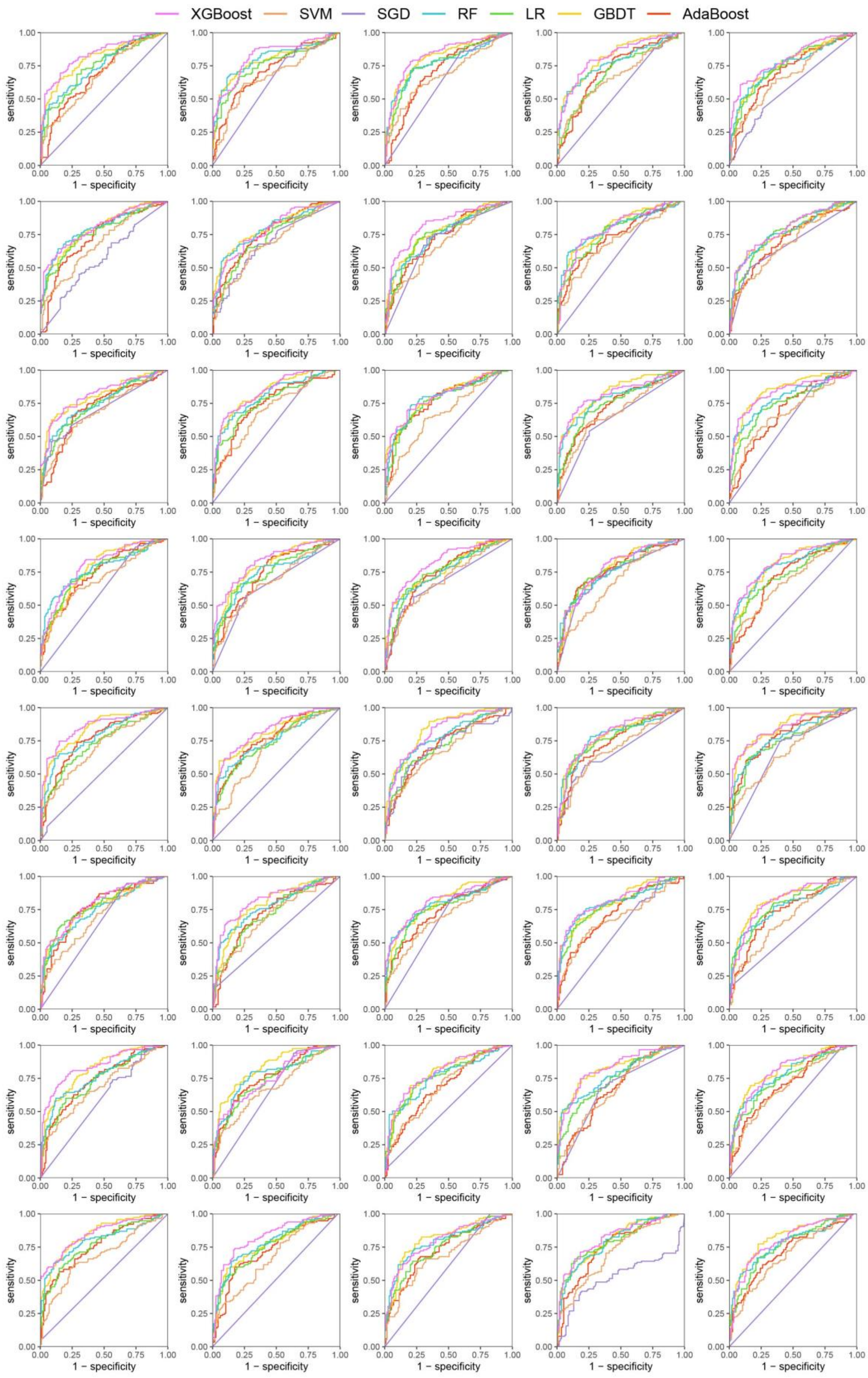

Supplementary Figure 18. ROC curves of test dataset of different machine learning methods trained 40 random downsampling sub-training datasets utilizing mutual information (MI) feature selection strategy.

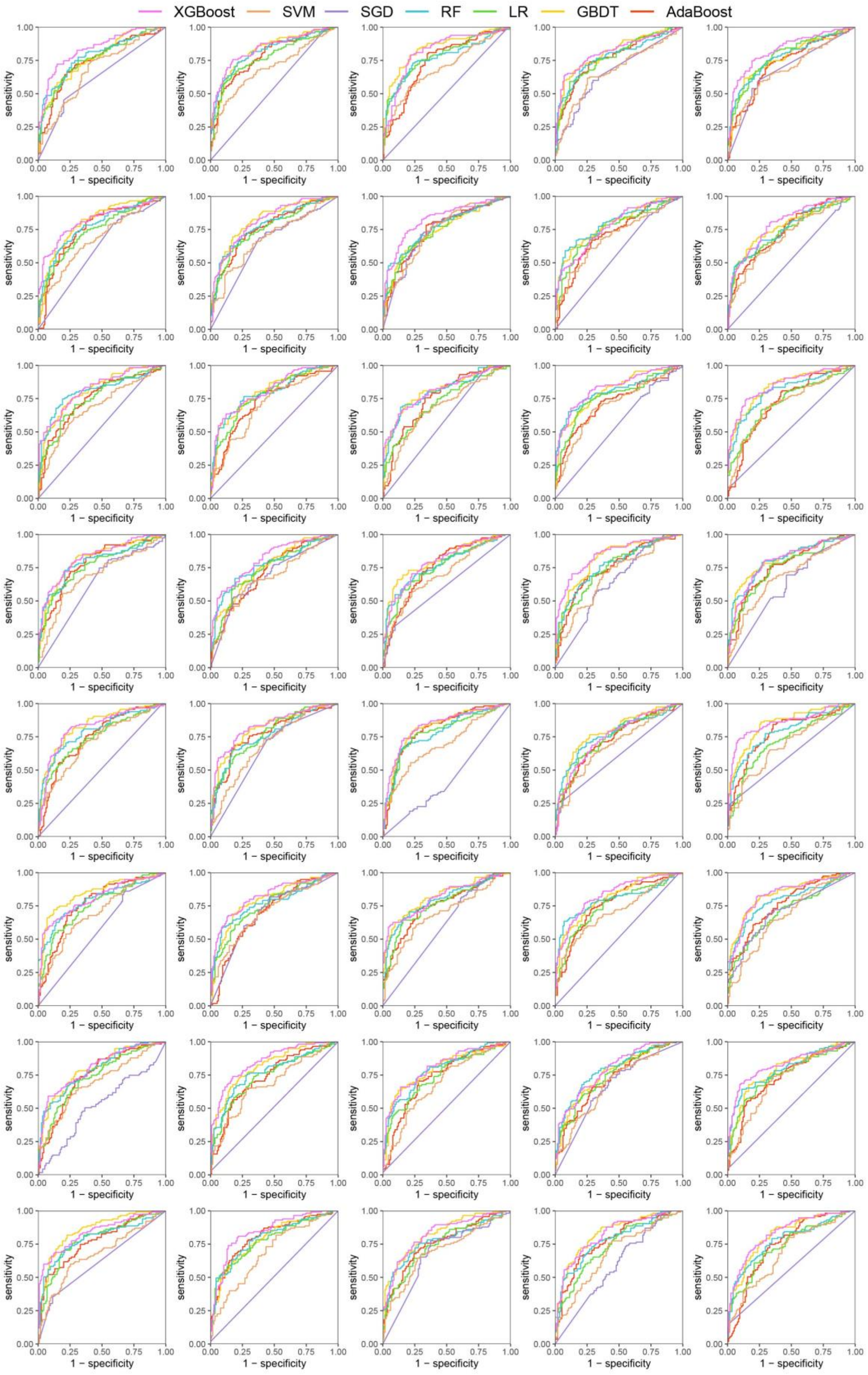

Supplementary Figure 19. ROC curves of test dataset of different machine learning methods trained on 40 random downsampling sub-training datasets utilizing max-relevance and min-redundancy (mRMR) feature selection strategy.

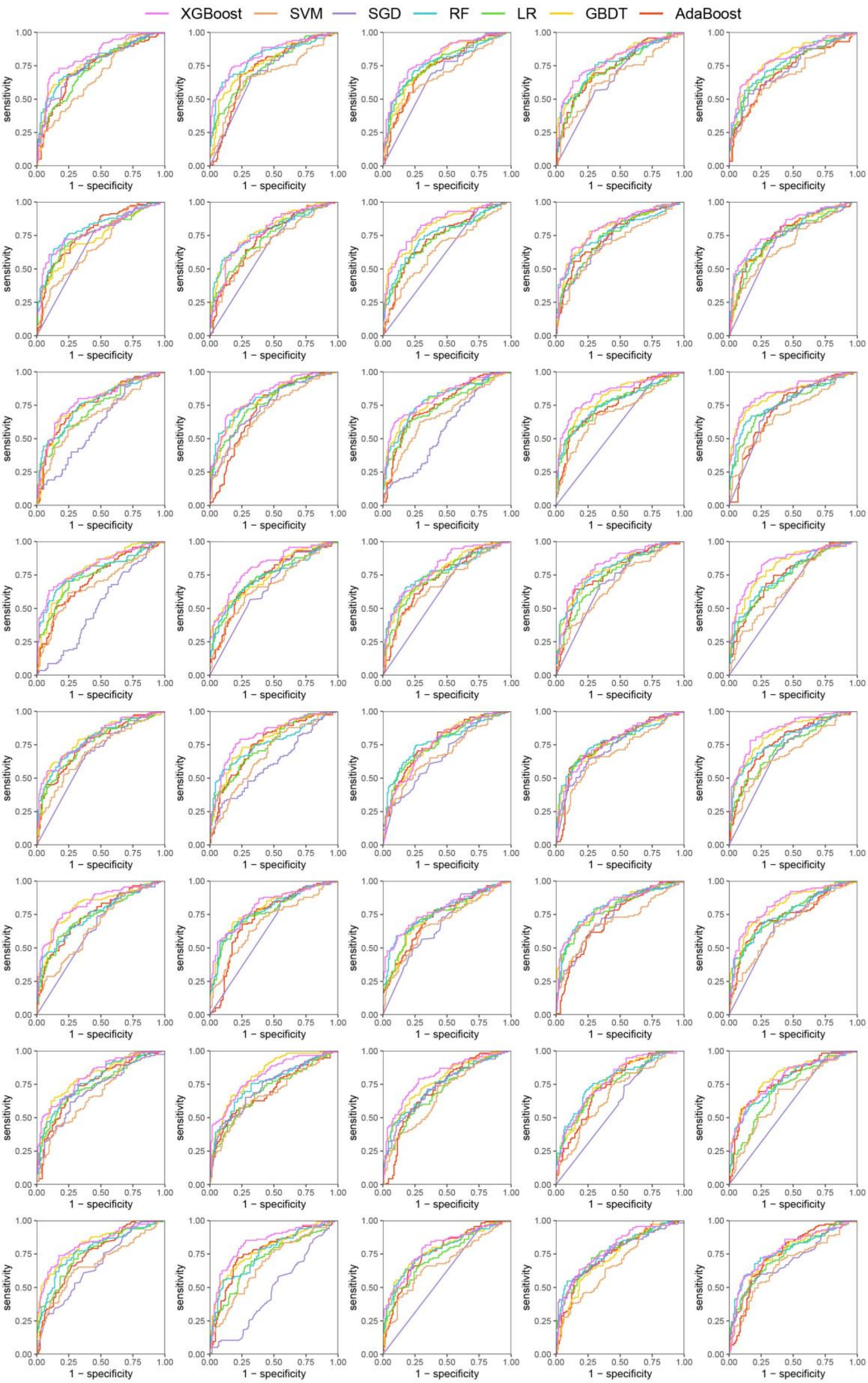

Supplementary Figure 20. ROC curves of test dataset of different machine learning methods trained on 40 random downsampling sub-training datasets utilizing recursive feature elimination (RFE) feature selection strategy.

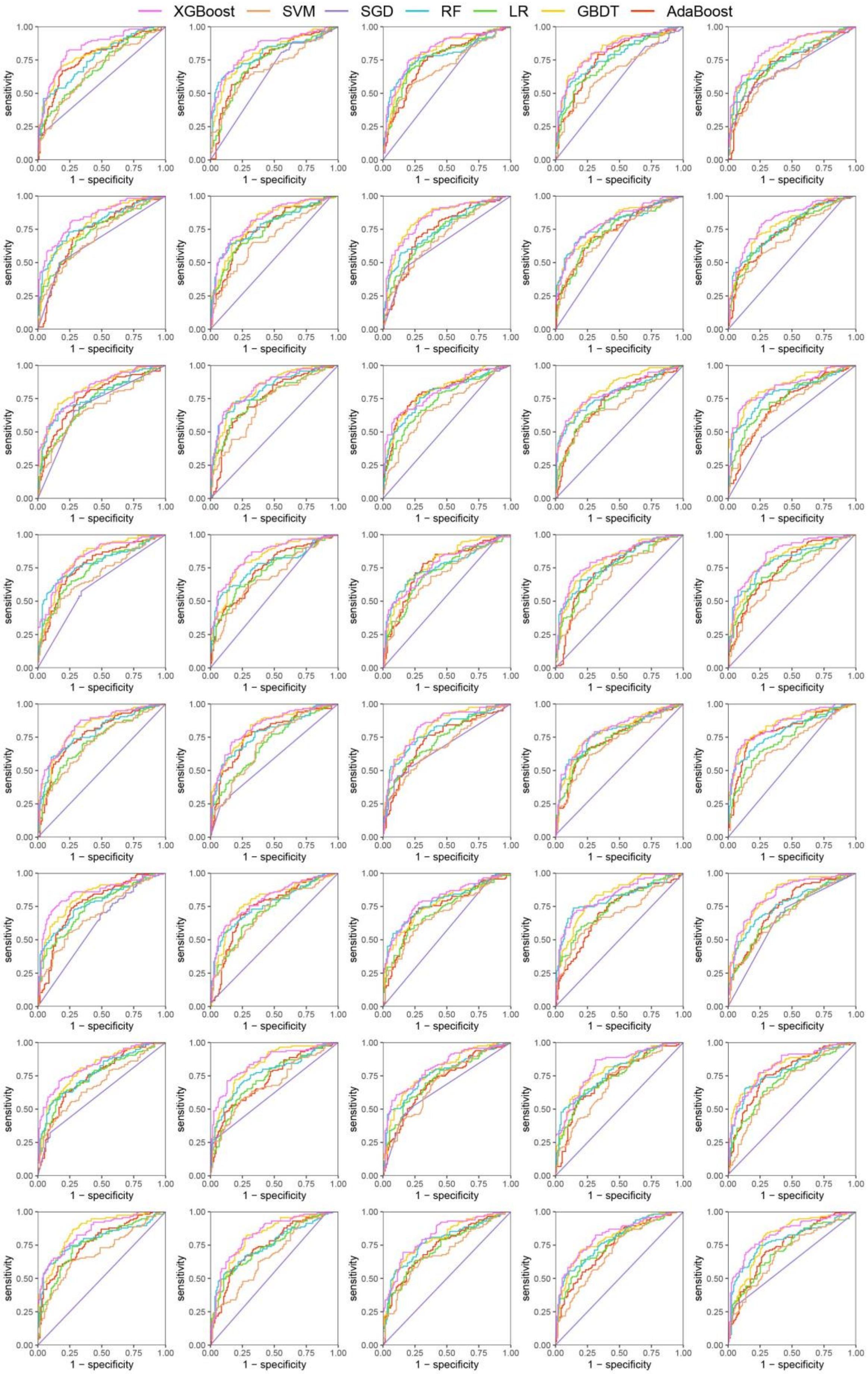

Supplementary Figure 21. Permutation feature importance of the top 5 machine learning model and feature selection method combinations based on AUC values using 40 random downsampling datasets (Part 1). Each bar indicates the average feature importance value, and the black line presents the standard deviation of each feature importance. Each method is shown in the format: {downsampling trial number}_{feature selection name}_{classifier}_{number of selected features}. These methods are (a) 1_MI_GBDT_20, (b) 1_MI_XGBoost_100, (c) 1_mRMR_XGBoost_45, (d) 1_mRMR_GBDT_80, (e) 1_RFE_GBDT_20, (f) 2_MI_GBDT_10, (g) 2_MI_XGBoost_100, (h) 2_mRMR_GBDT_70, (i) 2_RFE_XGBoost_70, (j) 2_RFE_GBDT_80, (k) 3_F_RF_35, (l) 3_MI_XGBoost_70, (m) 3_mRMR_RF_60, (n) 3_RFE_XGBoost_20, (o) 3_RFE_RF_50, (p) 4_Chi2_XGBoost_95, (q) 4_F_XGBoost_100, (r) 4_MI_XGBoost_50, (s) 4_RFE_XGBoost_45, (t) 4_RFE_GBDT_45, (u) 5_MI_XGBoost_85, (v) 5_mRMR_XGBoost_85, (w) 5_mRMR_GBDT_95, (x) 5_RFE_XGBoost_30, and (y) 5_RFE_GBDT_85. WHR=waist-hip ratio. SBP=systolic blood pressure. DBP=diastolic blood pressure. DLQI=dermatology life quality index. ESR=erythrocyte sedimentation rate. F=F-statistic.

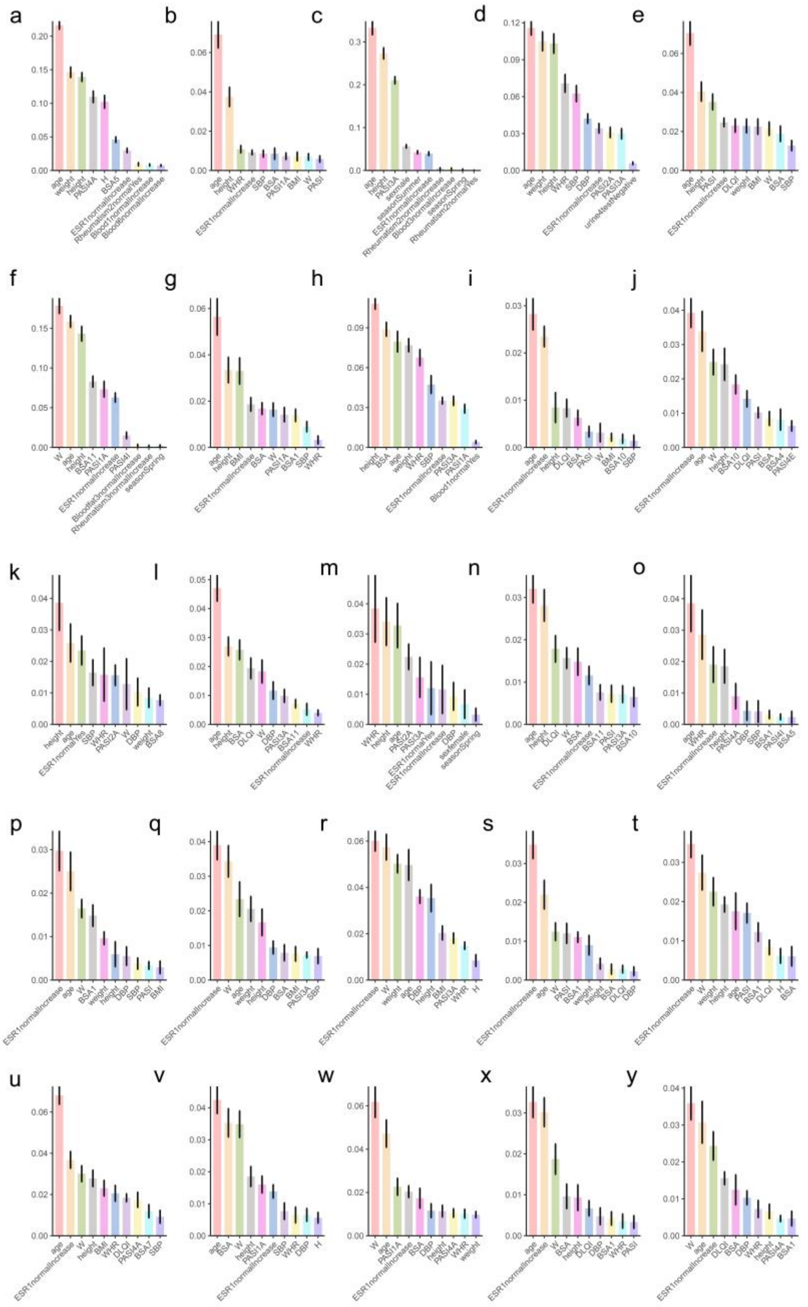

Supplementary Figure 22. Permutation feature importance of the top 5 machine learning model and feature selection method combinations based on AUC values using 40 random downsampling datasets (Part 2). Each bar indicates the average feature importance value, and the black line presents the standard deviation of each feature importance. Each method is shown in the format: {downsampling trial number}_{feature selection name}_{classifier}_{number of selected features}. These methods are (a) 6_MI_GBDT_35, (b) 6_mRMR_LR_40, (c) 6_mRMR_RF_50, (d) 6_mRMR_GBDT_75, (e) 6_RFE_XGBoost_90, (f) 7_MI_XGBoost_70, (g) 7_MI_GBDT_70, (h) 7_mRMR_RF_85, (i) 7_RFE_GBDT_25, (j) 7_RFE_XGBoost_25, (k) 8_MI_GBDT_15, (l) 8_MI_XGBoost_55, (m) 8_mRMR_GBDT_100, (n) 8_RFE_XGBoost_30, (o) 8_RFE_GBDT_75, (p) 9_MI_XGBoost_75, (q) 9_MI_GBDT_100, (r) 9_mRMR_RF_65, (s) 9_mRMR_GBDT_95, (t) 9_RFE_RF_50, (u) 10_MI_GBDT_75, (v) 10_MI_XGBoost_75, (w) 10_mRMR_GBDT_60, (x) 10_mRMR_XGBoost_75, and (y) 10_RFE_XGBoost_75. W=waistline. H=hipline. WHR=waist-hip ratio. SBP=systolic blood pressure. DBP=diastolic blood pressure. DLQI=dermatology life quality index. ESR=erythrocyte sedimentation rate. F=F-statistic.

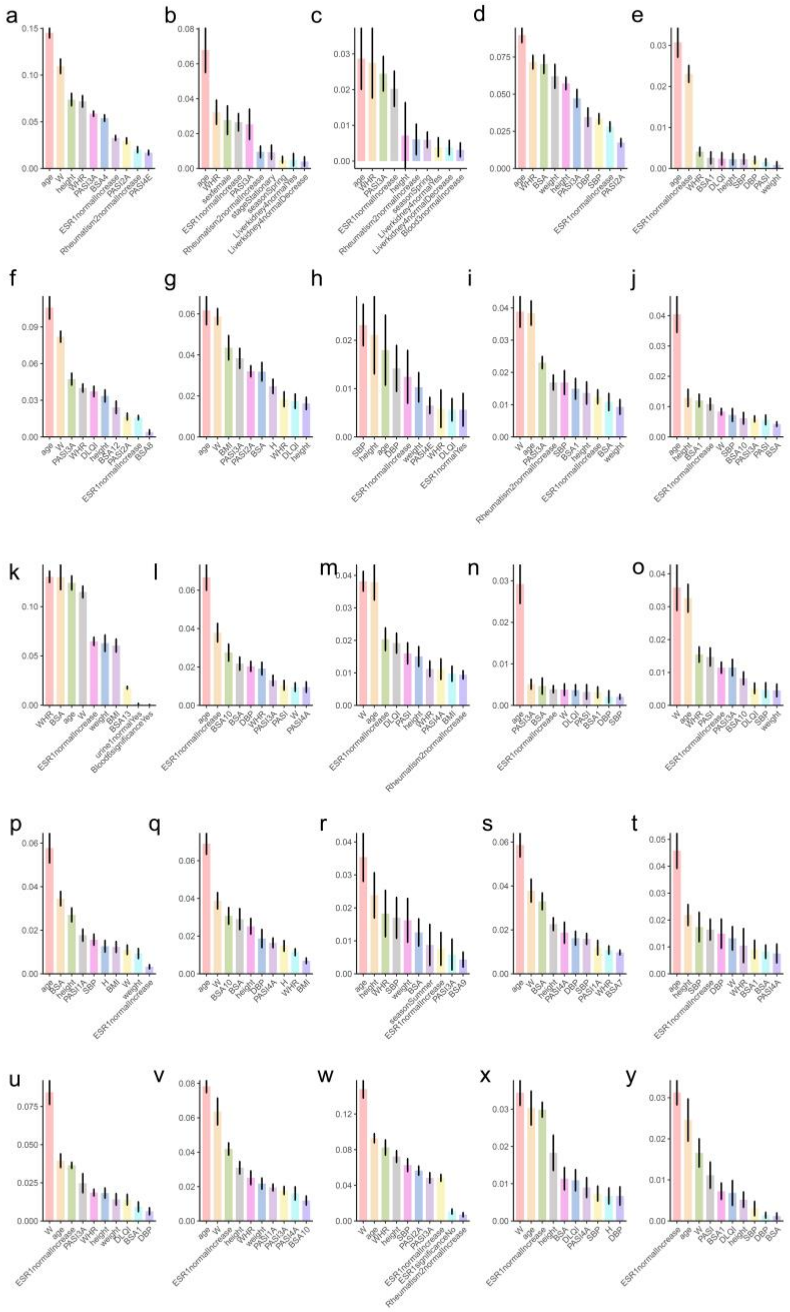

Supplementary Figure 23. Permutation feature importance of the top 5 machine learning model and feature selection method combinations based on AUC values using 40 random downsampling datasets (Part 3). Each bar indicates the average feature importance value, and the black line presents the standard deviation of each feature importance. Each method is shown in the format: {downsampling trial number}_{feature selection name}_{classifier}_{number of selected features}. These methods are (a) 11_Chi2_XGBoost_45, (b) 11_MI_XGBoost_55, (c) 11_MI_GBDT_95, (d) 11_RFE_RF_10, (e) 11_RFE_XGBoost_30, (f) 12_Chi2_GBDT_60, (g) 12_F_GBDT_70, (h) 12_MI_XGBoost_40, (i) 12_MI_GBDT_40, (j) 12_mRMR_GBDT_90, (k) 13_MI_XGBoost_20, (l) 13_mRMR_GBDT_80, (m) 13_mRMR_XGBoost_85, (n) 13_RFE_XGBoost_25, (o) 13_RFE_GBDT_35, (p) 14_F_GBDT_80, (q) 14_MI_GBDT_60, (r) 14_MI_XGBoost_80, (s) 14_mRMR_GBDT_100, (t) 14_mRMR_XGBoost_100, (u) 15_MI_GBDT_50, (v) 15_MI_XGBoost_55, (w) 15_mRMR_XGBoost_85, (x) 15_RFE_XGBoost_15, and (y) 15_RFE_GBDT_20. W=waistline. H=hipline. WHR=waist-hip ratio. SBP=systolic blood pressure. DBP=diastolic blood pressure. DLQI=dermatology life quality index. ESR=erythrocyte sedimentation rate. F=F-statistic.

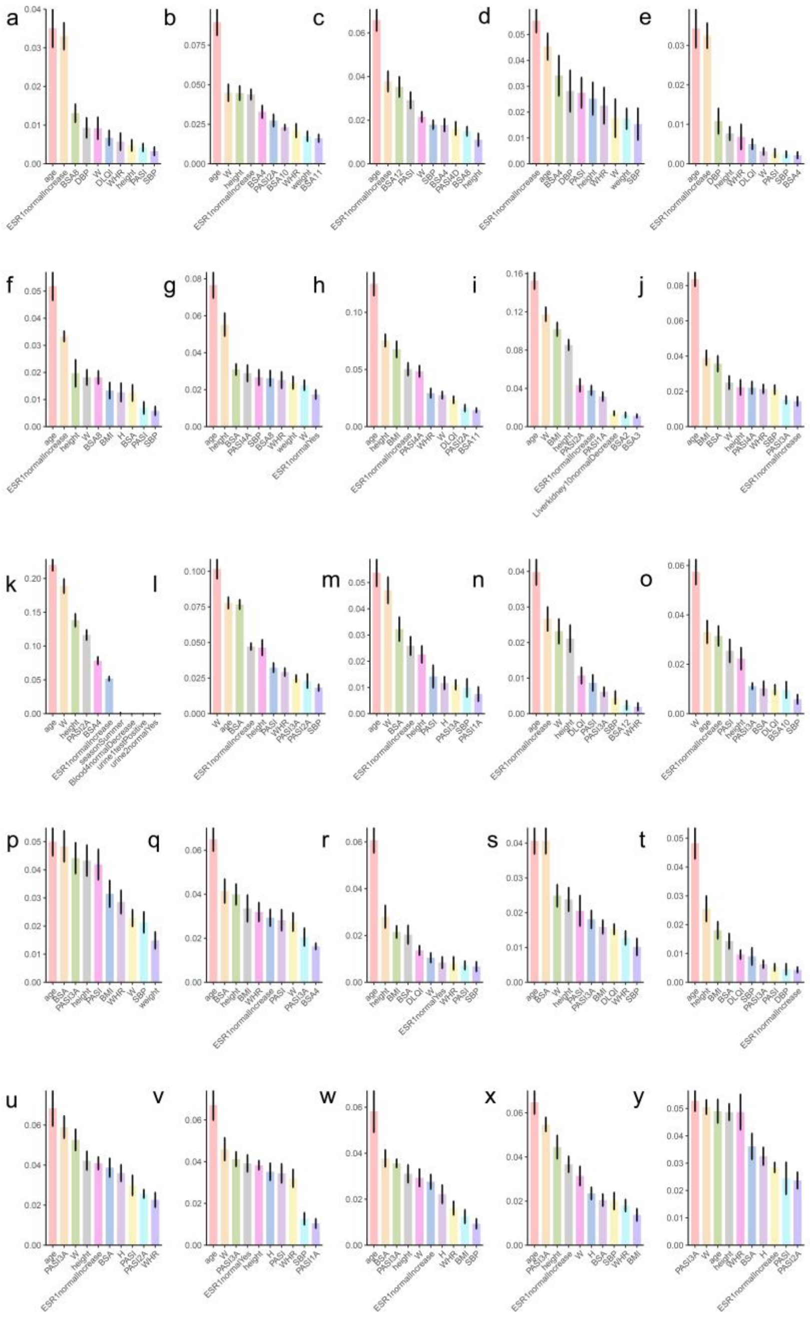

Supplementary Figure 24. Permutation feature importance of the top 5 machine learning model and feature selection method combinations based on AUC values using 40 random downsampling datasets (Part 4). Each bar indicates the average feature importance value, and the black line presents the standard deviation of each feature importance. Each method is shown in the format: {downsampling trial number}_{feature selection name}_{classifier}_{number of selected features}. These methods are (a) 16_F_RF_15, (b) 16_MI_RF_15, (c) 16_mRMR_LR_45, (d) 16_mRMR_RF_85, (e) 16_RFE_RF_25, (f) 17_MI_GBDT_65, (g) 17_MI_XGBoost_65, (h) 17_mRMR_GBDT_90, (i) 17_RFE_GBDT_70, (j) 17_RFE_XGBoost_85, (k) 18_MI_XGBoost_30, (l) 18_MI_GBDT_45, (m) 18_mRMR_RF_75, (n) 18_mRMR_GBDT_85, (o) 18_RFE_RF_15, (p) 19_MI_XGBoost_50, (q) 19_MI_GBDT_50, (r) 19_mRMR_GBDT_80, (s) 19_mRMR_XGBoost_80, (t) 19_RFE_XGBoost_10, (u) 20_Chi2_XGBoost_95, (v) 20_MI_GBDT_20, (w) 20_MI_XGBoost_35, (x) 20_RFE_GBDT_30, and (y) 20_RFE_XGBoost_90. W=waistline. H=hipline. WHR=waist-hip ratio. SBP=systolic blood pressure. DBP=diastolic blood pressure. DLQI=dermatology life quality index. ESR=erythrocyte sedimentation rate. F=F-statistic.

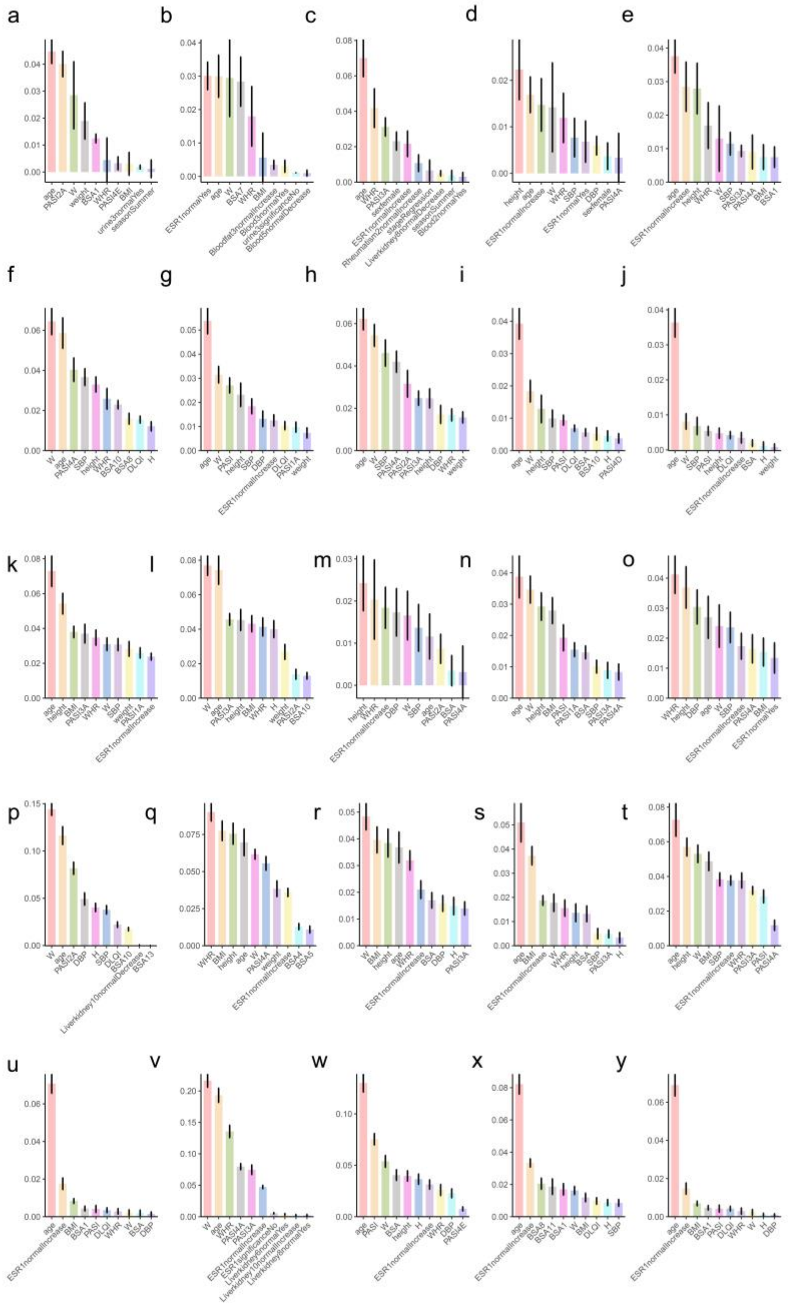

Supplementary Figure 25. Permutation feature importance of the top 5 machine learning model and feature selection method combinations based on AUC values using 40 random downsampling datasets (Part 5). Each bar indicates the average feature importance value, and the black line presents the standard deviation of each feature importance. Each method is shown in the format: {downsampling trial number}_{feature selection name}_{classifier}_{number of selected features}. These methods are (a) 21_MI_XGBoost_60, (b) 21_MI_RF_70, (c) 21_mRMR_RF_60, (d) 21_mRMR_GBDT_70, (e) 21_mRMR_XGBoost_75, (f) 22_MI_XGBoost_70, (g) 22_mRMR_GBDT_55, (h) 22_mRMR_XGBoost_70, (i) 22_RFE_GBDT_30, (j) 22_RFE_XGBoost_35, (k) 23_Chi2_GBDT_45, (l) 23_MI_XGBoost_90, (m) 23_RFE_GBDT_45, (n) 23_RFE_XGBoost_70, (o) 23_RFE_RF_95, (p) 24_F_XGBoost_35, (q) 24_MI_XGBoost_30, (r) 24_mRMR_GBDT_55, (s) 24_mRMR_XGBoost_60, (t) 24_RFE_XGBoost_30, (u) 25_Chi2_GBDT_60, (v) 25_MI_XGBoost_60, (w) 25_mRMR_GBDT_80, (x) 25_mRMR_XGBoost_100, and (y) 25_RFE_XGBoost_25. W=waistline. H=hipline. WHR=waist-hip ratio. SBP=systolic blood pressure. DBP=diastolic blood pressure. DLQI=dermatology life quality index. ESR=erythrocyte sedimentation rate. F=F-statistic.

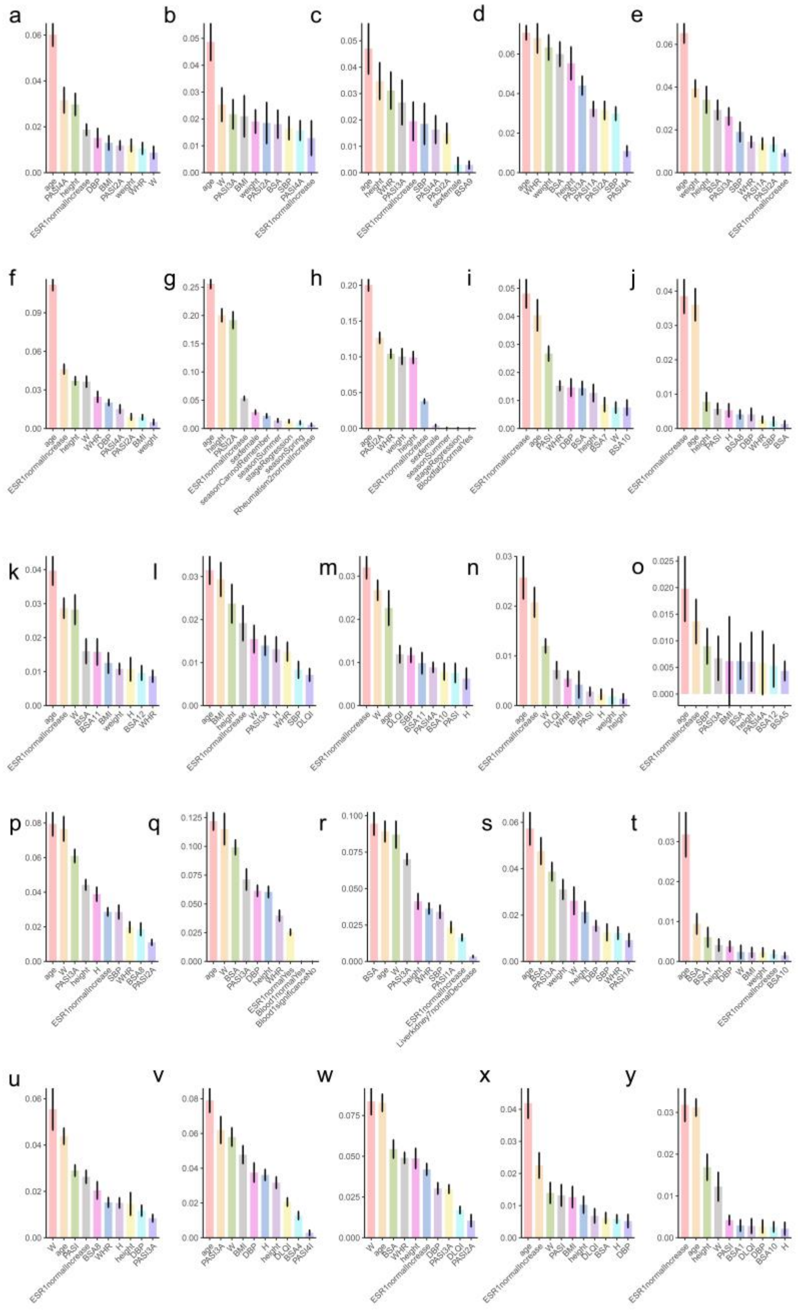

Supplementary Figure 26. Permutation feature importance of the top 5 machine learning model and feature selection method combinations based on AUC values using 40 random downsampling datasets (Part 6). Each bar indicates the average feature importance value, and the black line presents the standard deviation of each feature importance. Each method is shown in the format: {downsampling trial number}_{feature selection name}_{classifier}_{number of selected features}. These methods are (a) 26_Chi2_XGBoost_50, (b) 26_Chi2_GBDT_50, (c) 26_mRMR_LR_60, (d) 26_RFE_GBDT_30, (e) 26_RFE_XGBoost_50, (f) 27_Chi2_XGBoost_90, (g) 27_MI_XGBoost_60, (h) 27_mRMR_GBDT_50, (i) 27_mRMR_XGBoost_55, (j) 27_RFE_XGBoost_25, (k) 28_Chi2_XGBoost_55, (l) 28_F_XGBoost_85, (m) 28_MI_XGBoost_50, (n) 28_mRMR_XGBoost_85, (o) 28_RFE_XGBoost_35, (p) 29_Chi2_GBDT_75, (q) 29_MI_XGBoost_75, (r) 29_MI_GBDT_90, (s) 29_RFE_XGBoost_25, (t) 29_RFE_GBDT_35, (u) 30_MI_RF_30, (v) 30_MI_XGBoost_100, (w) 30_mRMR_GBDT_90, (x) 30_RFE_XGBoost_25, and (y) 30_RFE_GBDT_55. W=waistline. H=hipline. WHR=waist-hip ratio. SBP=systolic blood pressure. DBP=diastolic blood pressure. DLQI=dermatology life quality index. ESR=erythrocyte sedimentation rate. F=F-statistic.

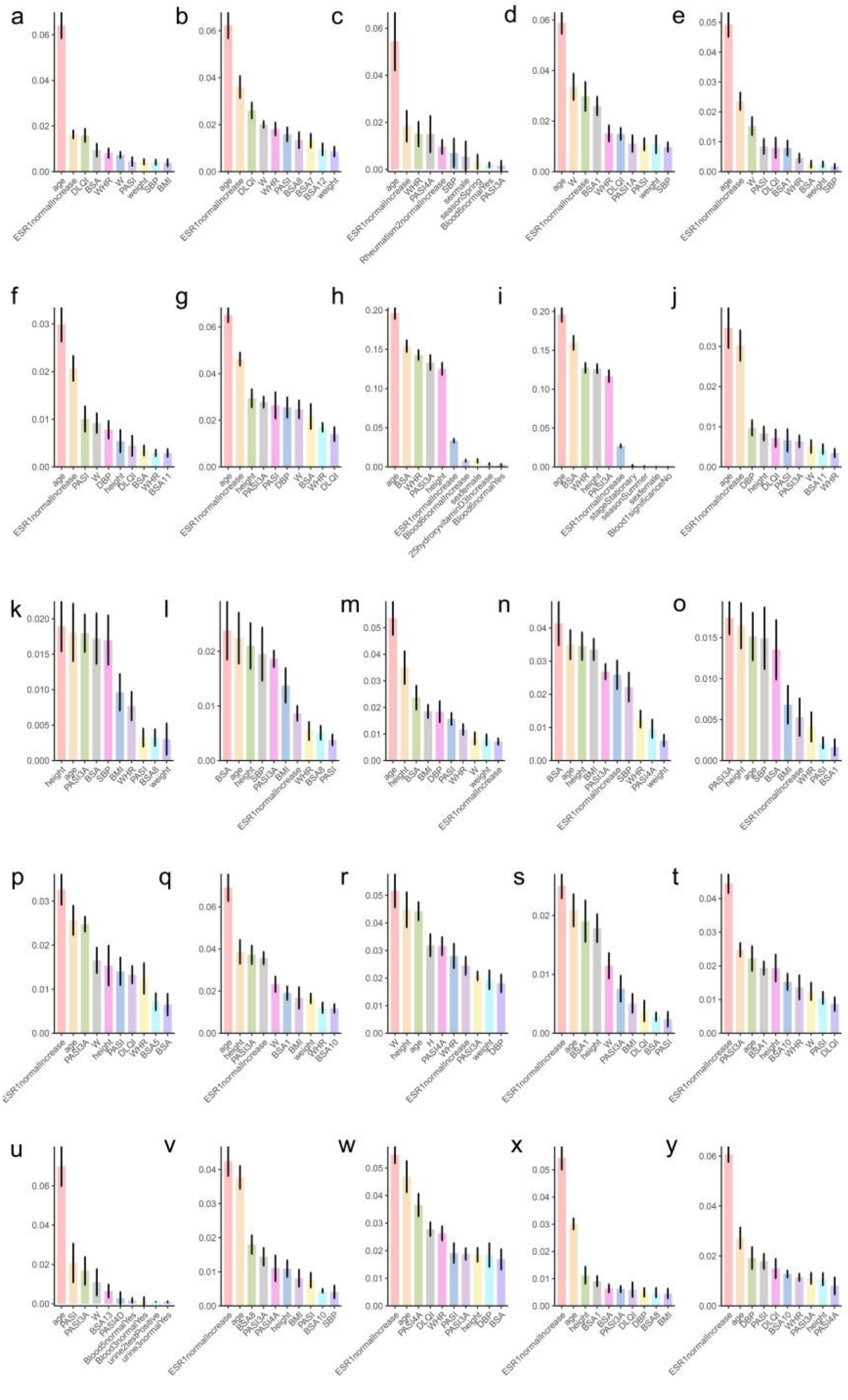

Supplementary Figure 27. Permutation feature importance of the top 5 machine learning model and feature selection method combinations based on AUC values using 40 random downsampling datasets (Part 7). Each bar indicates the average feature importance value, and the black line presents the standard deviation of each feature importance. Each method is shown in the format: {downsampling trial number}_{feature selection name}_{classifier}_{number of selected features}. These methods are (a) 31_Chi2_XGBoost_65, (b) 31_F_XGBoost_65, (c) 31_MI_XGBoost_70, (d) 31_MI_GBDT_70, (e) 31_RFE_XGBoost_90, (f) 32_F_GBDT_90, (g) 32_MI_GBDT_85, (h) 32_mRMR_AdaBoost_60, (i) 32_mRMR_GBDT_90, (j) 32_RFE_GBDT_60, (k) 33_Chi2_XGBoost_75, (l) 33_MI_XGBoost_100, (m) 33_mRMR_GBDT_95, (n) 33_RFE_GBDT_25, (o) 33_RFE_XGBoost_45, (p) 34_MI_GBDT_60, (q) 34_MI_XGBoost_85, (r) 34_mRMR_XGBoost_75, (s) 34_mRMR_GBDT_80, (t) 34_RFE_XGBoost_80, (u) 35_MI_XGBoost_35, (v) 35_mRMR_GBDT_85, (w) 35_mRMR_XGBoost_85, (x) 35_RFE_XGBoost_35, and (y) 35_RFE_GBDT_45. W=waistline. H=hipline. WHR=waist-hip ratio. SBP=systolic blood pressure. DBP=diastolic blood pressure. DLQI=dermatology life quality index. ESR=erythrocyte sedimentation rate. F=F-statistic.

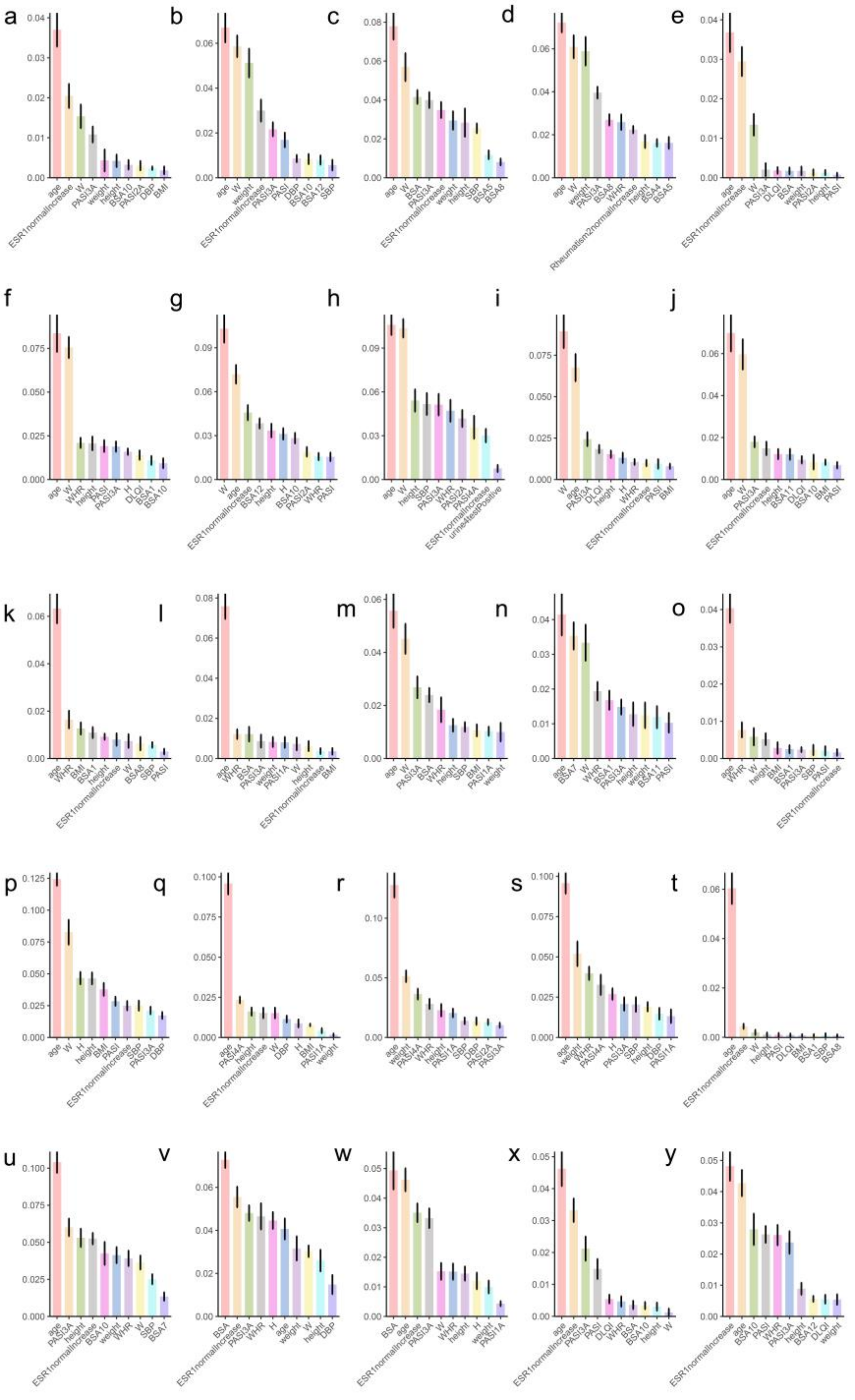

Supplementary Figure 28. Permutation feature importance of the top 5 machine learning model and feature selection method combinations based on AUC values using 40 random downsampling datasets (Part 8). Each bar indicates the average feature importance value, and the black line presents the standard deviation of each feature importance. Each method is shown in the format: {downsampling trial number}_{feature selection name}_{classifier}_{number of selected features}. These methods are (a) 36_F_GBDT_100, (b) 36_MI_XGBoost_60, (c) 36_MI_GBDT_75, (d) 36_RFE_GBDT_80, (e) 36_RFE_XGBoost_85, (f) 37_MI_GBDT_65, (g) 37_MI_XGBoost_75, (h) 37_mRMR_GBDT_100, (i) 37_RFE_XGBoost_45, (j) 37_RFE_GBDT_100, (k) 38_Chi2_GBDT_85, (l) 38_F_GBDT_100, (m) 38_MI_GBDT_30, (n) 38_MI_XGBoost_100, (o) 38_RFE_XGBoost_50, (p) 39_Chi2_XGBoost_95, (q) 39_F_XGBoost_70, (r) 39_MI_GBDT_25, (s) 39_MI_XGBoost_40, (t) 39_mRMR_XGBoost_65, (u) 40_MI_XGBoost_55, (v) 40_mRMR_GBDT_60, (w) 40_mRMR_XGBoost_75, (x) 40_mRMR_RF_75, and (y) 40_RFE_RF_10. W=waistline. H=hipline. WHR=waist-hip ratio. SBP=systolic blood pressure. DBP=diastolic blood pressure. DLQI=dermatology life quality index. ESR=erythrocyte sedimentation rate. F=F-statistic.

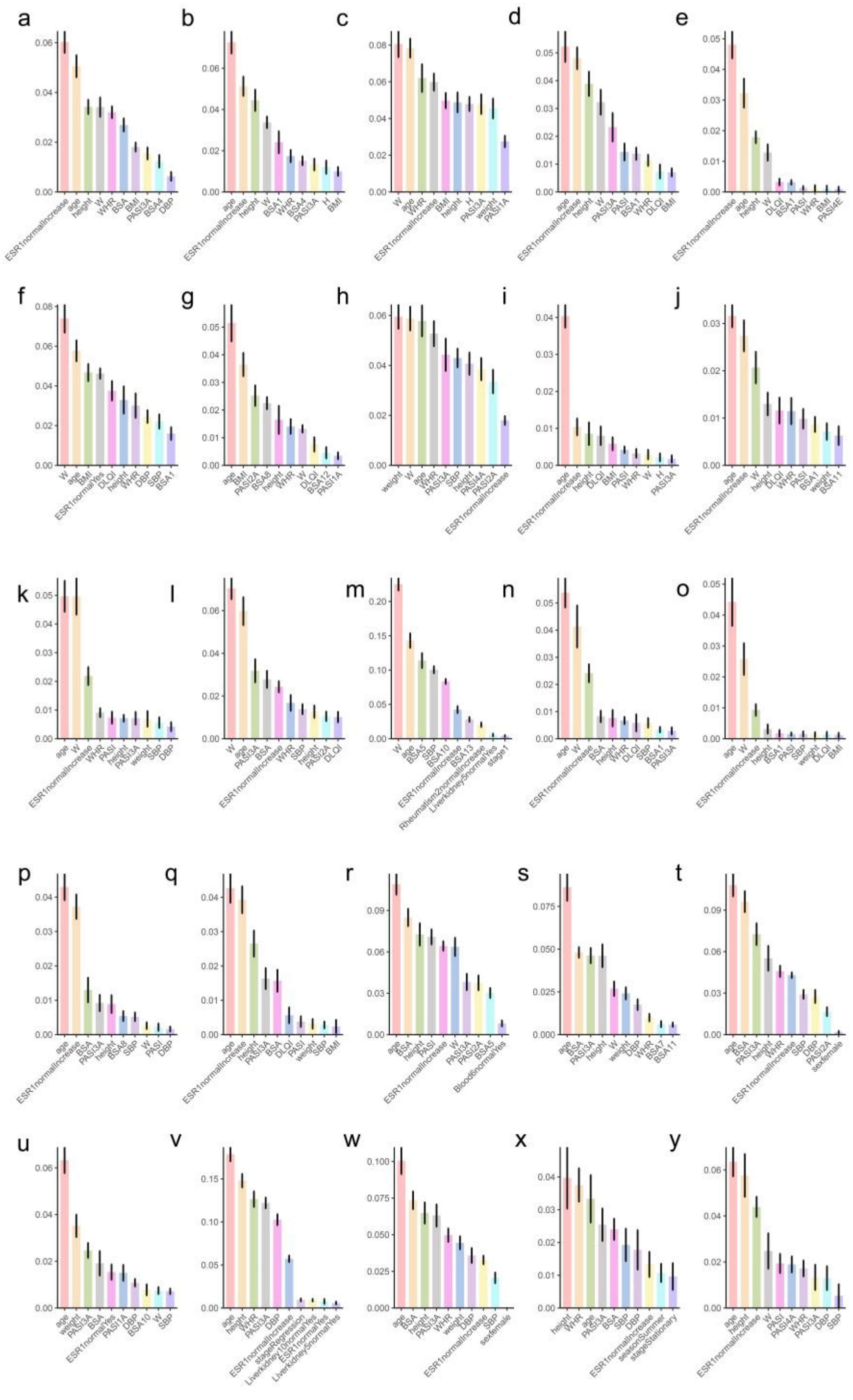

Supplementary Figure 29. A *post hoc* analysis grouped by features with *p*-value<0.01 tested by the Kruskal-Wallis H test. BloodRT=blood routine test. UrineRT=urine routine test. ESR=erythrocyte sedimentation rate.

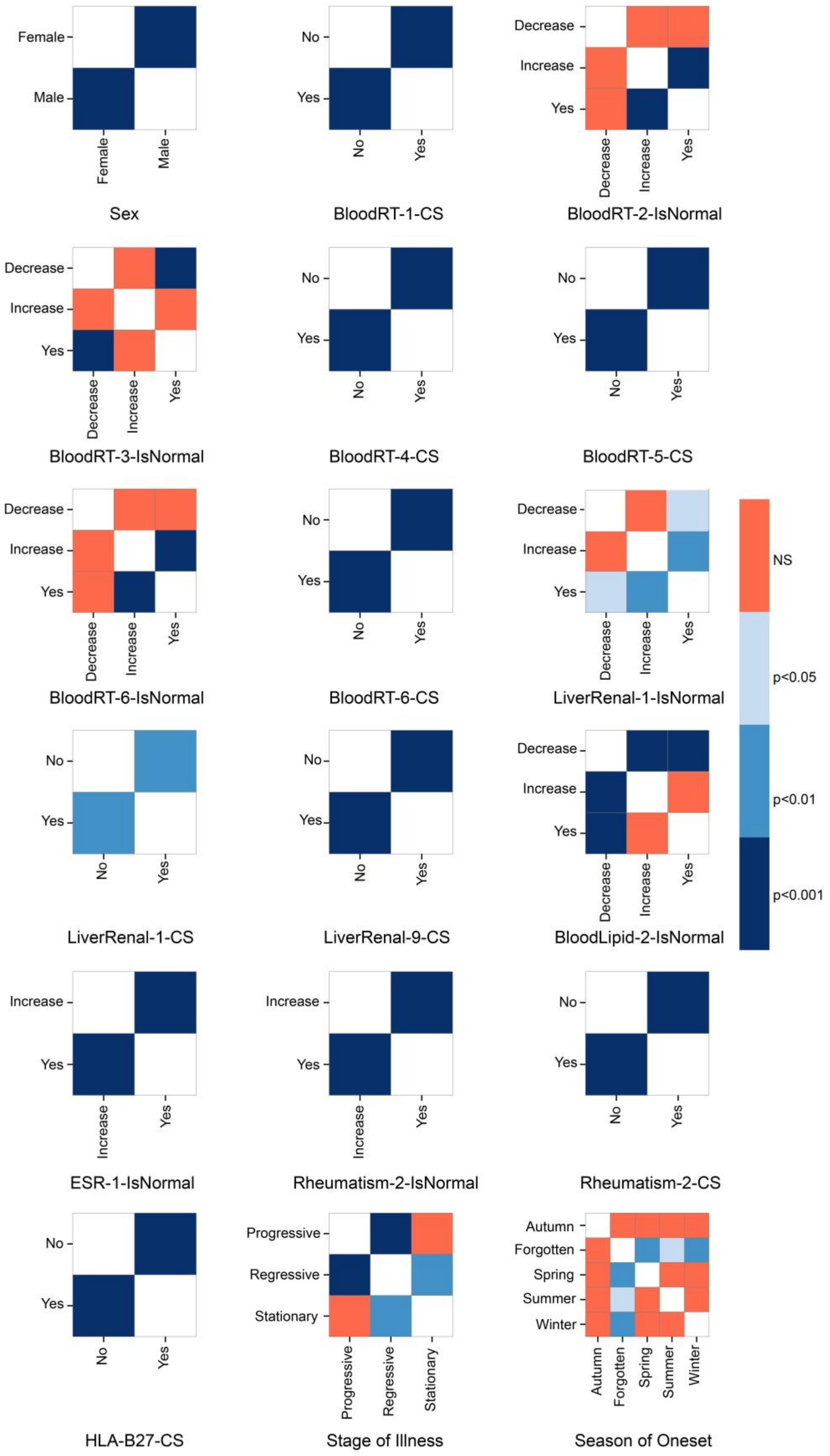

Supplementary Figure 30. A *post hoc* analysis grouped by features with 0.01≤*p*-value<0.05 tested by the Kruskal-Wallis H test. HLA-B27-1=HLA-B27 allotype.

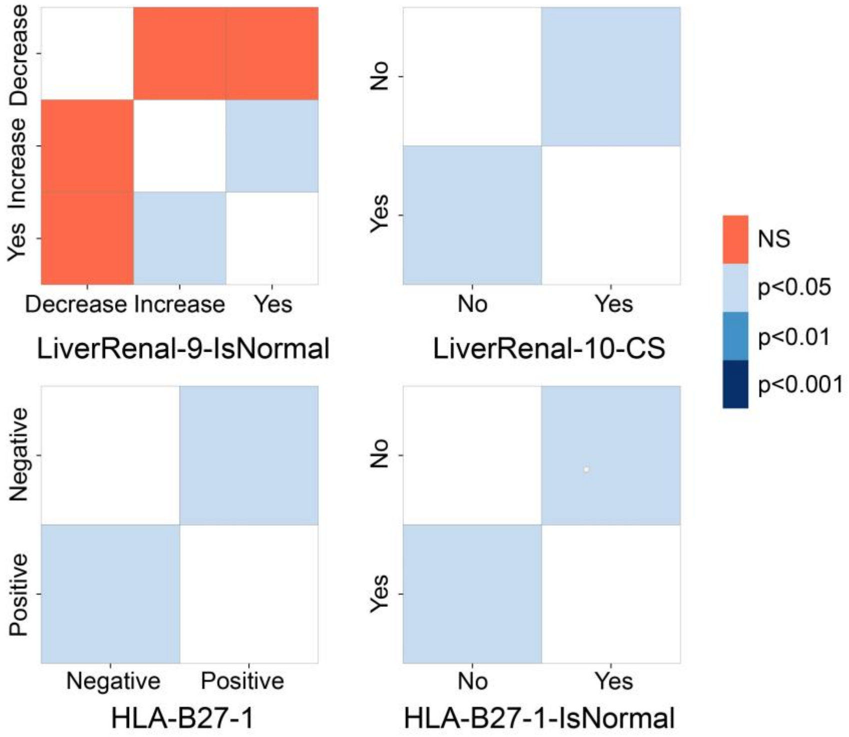

Supplementary Figure 31. A *post hoc* analysis grouped by PsA labels with *p*-value<0.01 tested by the Kruskal-Wallis H test. BloodRT=blood routine test. UrineRT=urine routine test. ESR=erythrocyte sedimentation rate. W=waistline. H=hipline. WHR=waist-hip ratio. SBP=systolic blood pressure. DBP=diastolic blood pressure. DLQI=dermatology life quality index.

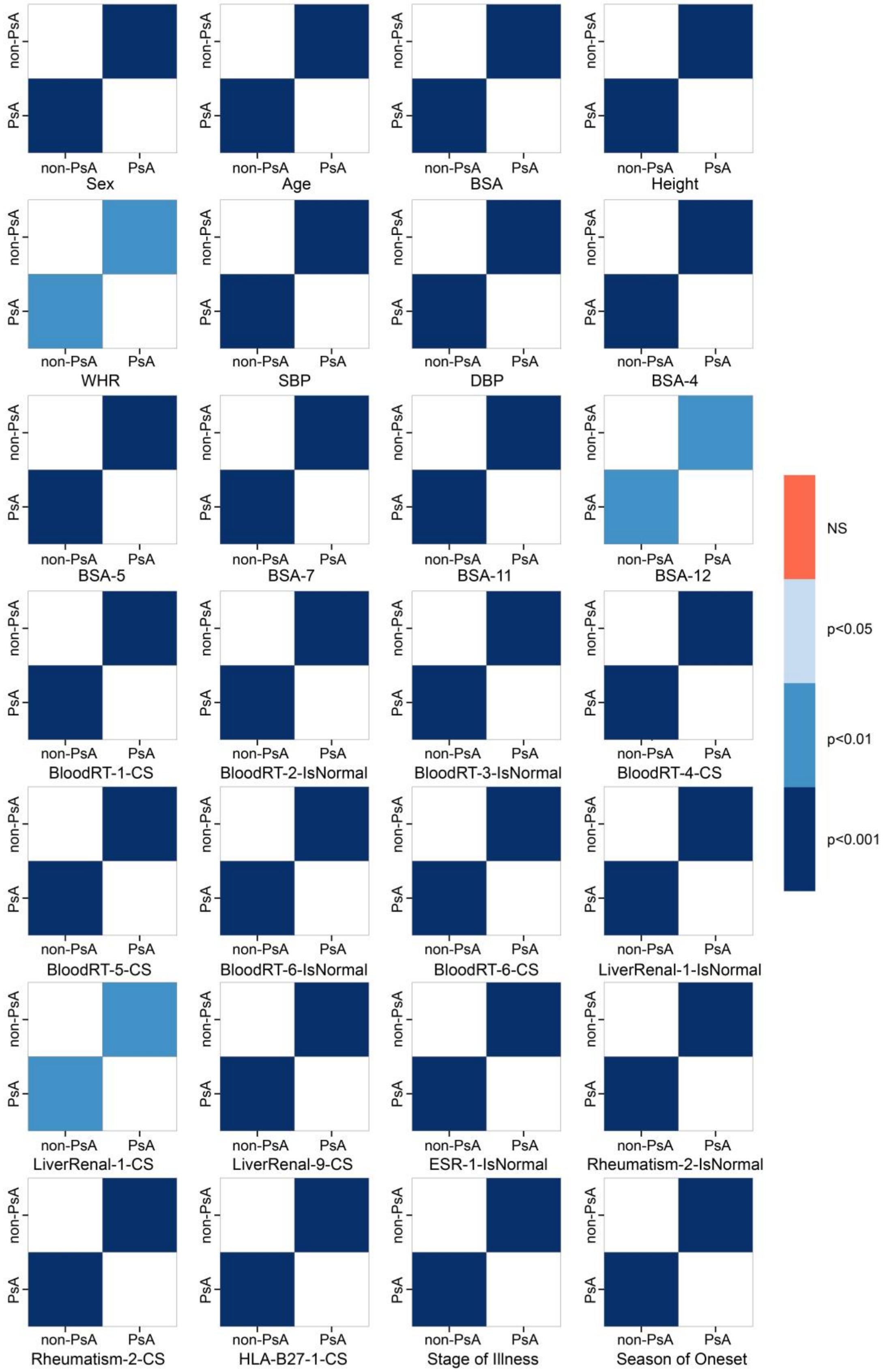

Supplementary Figure 32. A *post hoc* analysis grouped by PsA labels with 0.01≤p-value<0.01 tested by Kruskal-Wallis H test. W=waistline. HLA-B27=HLA-B27 allotype.

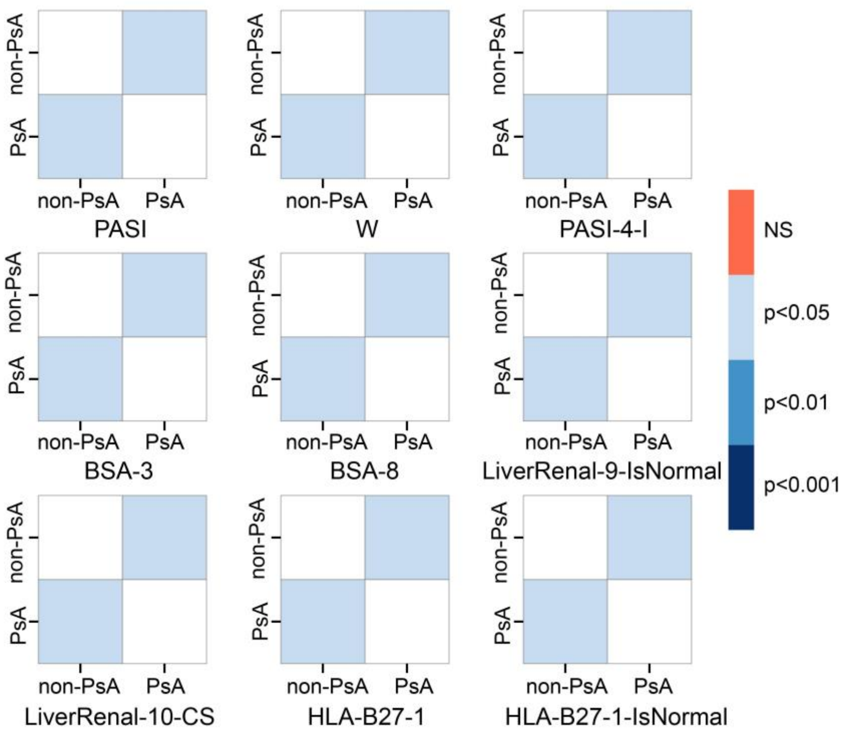

Supplementary Figure 33. The Jackknife test of different machine learning methods with different numbers of selected features for PsA risk prediction. (a) Chi-Square feature selection strategy. (b) F score feature selection strategy. (c) Mutual information feature selection strategy. (d) Max-relevance and min-redundancy feature (mRMR) selection strategy. (e) Recursive feature elimination feature selection strategy. (f) Sequential feature selection strategy (forward selection).

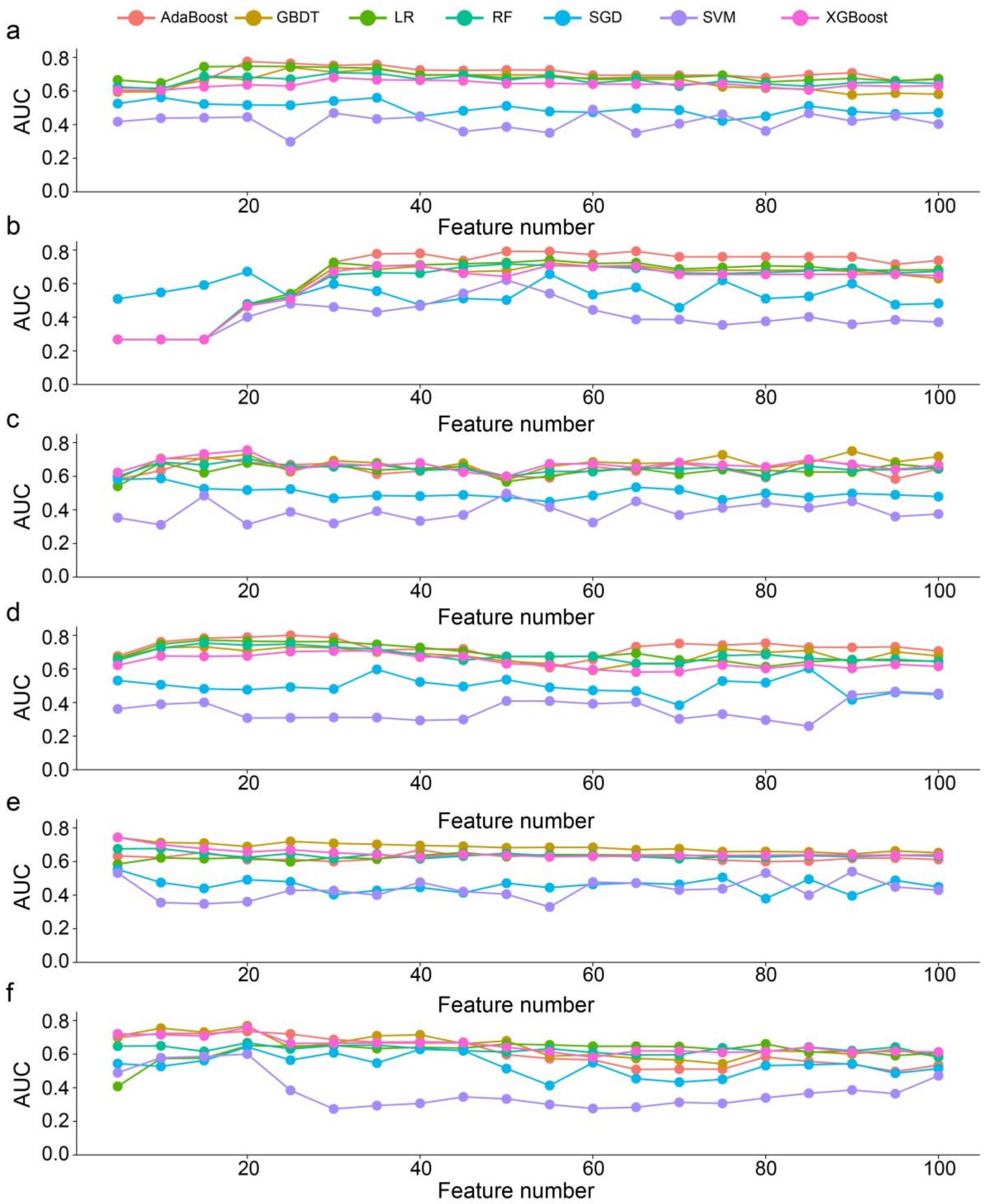

Supplementary Figure 34. The multivariate Cox regression for transformation from non-PsA to PsA based on the Lasso regression method. (a) The performance of Cox regression with the Lasso method under different feature selection numbers based on a 10-fold cross-validation test. (b) A forest plot of the selected Cox regression model based on the Lasso method. The *p*-value is obtained by the Log-Rank test. BloodRT=blood routine test. UrineRT=urine routine test. ESR=erythrocyte sedimentation rate. WHR=waist-hip ratio.

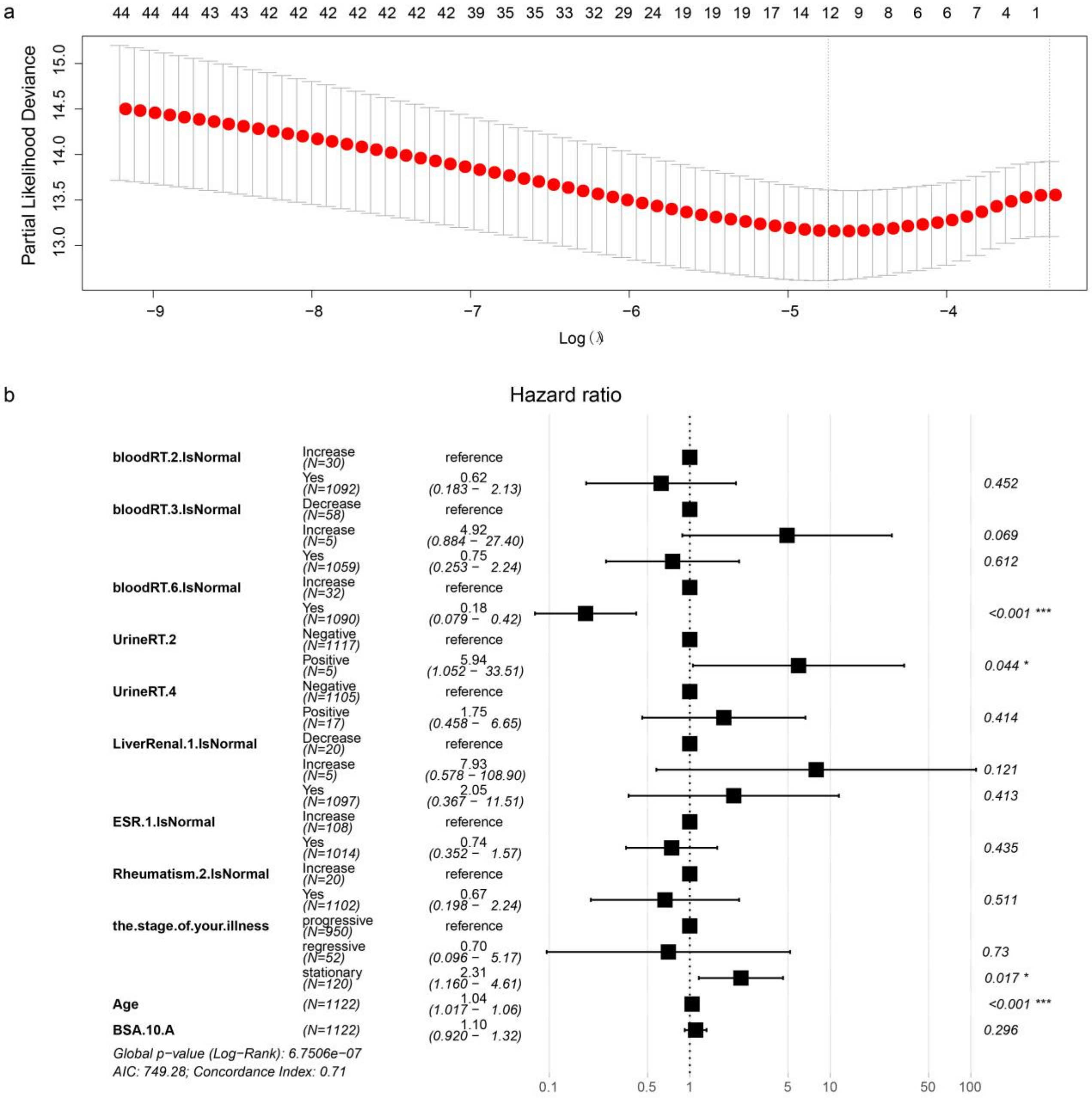

Supplementary Figure 35. The PASI changes over time presented in line plots (a) and dot plots (b). The grey lines and grey dots indicate that the patients were diagnosed with PsA at the corresponding time. TCS=topical corticosteroids. MTX=methotrexate. IL-17 inhibitors=interleukin-17 inhibitors. IL-23 inhibitors=interleukin-23 inhibitors. TNF-α inhibitors=tumor necrosis factor alpha inhibitors.

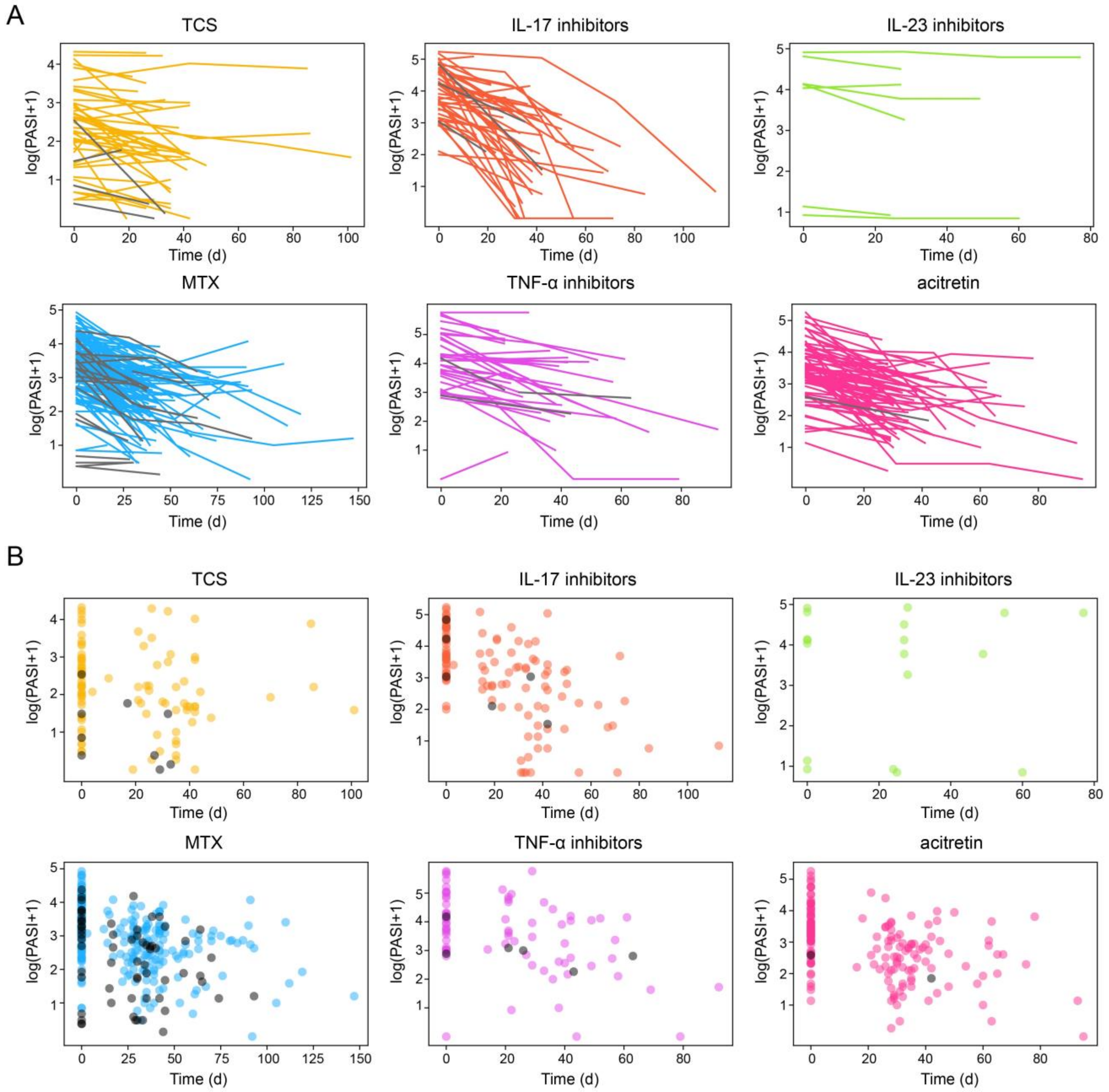

Supplementary Figure 36. The PASI changes over time of 17 newly collected patients treated with different drugs.
TCS=topical corticosteroids. MTX=methotrexate. IL-17 inhibitors=interleukin-17 inhibitors. IL-23 inhibitors=interleukin-23 inhibitors. TNF-α inhibitors=tumor necrosis factor alpha inhibitors.

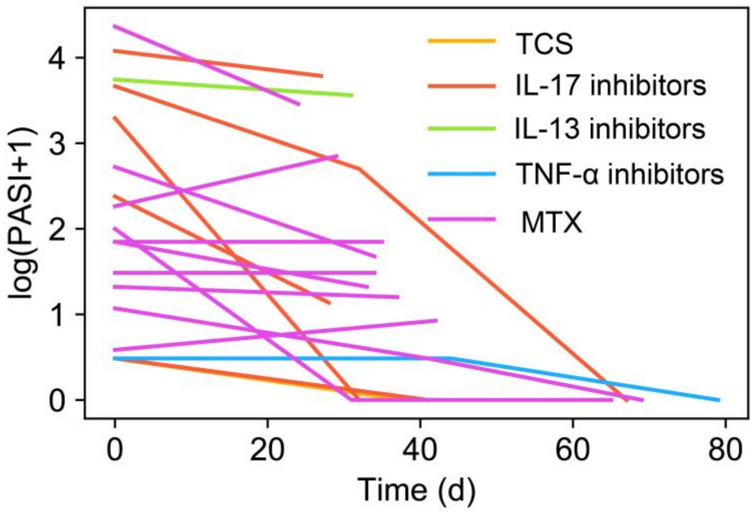

Supplement: Supplementary file 2 — Supplementary Materials [file 41746_2023_757_MOESM2_ESM.pdf]
